# Novel tricyclic small molecule inhibitors of Nicotinamide N-methyltransferase for the treatment of metabolic disorders

**DOI:** 10.1038/s41598-022-19634-2

**Published:** 2022-09-14

**Authors:** Sven Ruf, Sridharan Rajagopal, Sanjay Venkatachalapathi Kadnur, Mahanandeesha S. Hallur, Shilpa Rani, Rajendra Kristam, Srinivasan Swaminathan, Bharat Ravindra Zope, Pavan Kumar Gondrala, Indu Swamy, V. P. Rama Kishore Putta, Saravanan Kandan, Gernot Zech, Herman Schreuder, Christine Rudolph, Ralf Elvert, Joerg Czech, Swarnakumari Birudukota, M. Amir Siddiqui, Niranjan Naranapura Anand, Vishal Subhash Mane, Sreekanth Dittakavi, Juluri Suresh, Ramachandraiah Gosu, Mullangi Ramesh, Takeshi Yura, Saravanakumar Dhakshinamoorthy, Aimo Kannt

**Affiliations:** 1 Sanofi-Aventis Deutschland GmbH, R&D, Integrated Drug Discovery, Industriepark Hoechst, 65926 Frankfurt am Main, Germany; 2 Jubilant Therapeutics India Ltd, Bangalore, 560022 India; 3 Jubilant Biosys Ltd, Bangalore, 560022 India; 4 Evotec GmbH, Marie-Curie-Straße 7, 37079 Göttingen, Germany; 5 Fraunhofer-Institute for Translational Medicine and Pharmacology ITMP, Theodor-Stern-Kai 7, 60596 Frankfurt am Main, Germany; 6 Fraunhofer Leistungszentrum TheraNova, Theodor-Stern-Kai 6, 60596 Frankfurt am Main, Germany

**Keywords:** Lead optimization, Pharmacology, Drug discovery, Pharmacology

## Abstract

Nicotinamide N-methyltransferase (NNMT) is a metabolic regulator that catalyzes the methylation of nicotinamide (Nam) using the co-factor S-adenosyl-L-methionine to form 1-methyl-nicotinamide (MNA). Overexpression of NNMT and the presence of the active metabolite MNA is associated with a number of diseases including metabolic disorders. We conducted a high-throughput screening campaign that led to the identification of a tricyclic core as a potential NNMT small molecule inhibitor series. Elaborate medicinal chemistry efforts were undertaken and hundreds of analogs were synthesized to understand the structure activity relationship and structure property relationship of this tricyclic series. A lead molecule, JBSNF-000028, was identified that inhibits human and mouse NNMT activity, reduces MNA levels in mouse plasma, liver and adipose tissue, and drives insulin sensitization, glucose modulation and body weight reduction in a diet-induced obese mouse model of diabetes. The co-crystal structure showed that JBSNF-000028 binds below a hairpin structural motif at the nicotinamide pocket and stacks between Tyr-204 (from Hairpin) and Leu-164 (from central domain). JBSNF-000028 was inactive against a broad panel of targets related to metabolism and safety. Interestingly, the improvement in glucose tolerance upon treatment with JBSNF-000028 was also observed in NNMT knockout mice with diet-induced obesity, pointing towards the glucose-normalizing effect that may go beyond NNMT inhibition. JBSNF-000028 can be a potential therapeutic option for metabolic disorders and developmental studies are warranted.

## Introduction

Nicotinamide (Nam) belongs to vitamin B_3_ family and is a component of nicotinamide adenine dinucleotide (NAD^+^/NADH) and related molecules. It is metabolized by nicotinamide-N-methyltransferase (NNMT), a cytosolic enzyme that catalyzes the transfer of a methyl group from S-adenosyl-methionine (SAM) to Nam, yielding S-adenosyl-homocysteine (SAH) and 1-methylnicotinamide (MNA)^1,2^. NNMT is expressed in many tissues including liver, adipose tissue, and skeletal muscle^3^, and also in several human cancers^4^. Methylation of Nam to MNA is followed by urinary excretion or further metabolization to 1-methyl-2-pyridone-5-carboxamide (Me2PY) or 1-methyl-4-pyridone-5-carboxamide (Me4PY) in a reaction catalyzed by aldehyde oxidase^5^. Nam metabolism is exclusively regulated by NNMT, and systemic knockout of NNMT was found to fully prevent MNA formation^6,7^.

Besides regulating Nam availability as a substrate for NAD^+^ biosynthesis, NNMT activity also influences the SAM:SAH ratio, or cellular methylation potential, and regulates DNA and histone methylation^4^, thus being an important node in the control of cellular metabolism and epigenetic regulation^8^. The expression of *Nnmt*, the gene encoding for NNMT, was found to be increased in white adipose tissue (WAT) of mice with diet-induced obesity^9^ and of obese patients with type-2 diabetes^10^. MNA levels in plasma, serum and urine correlate with obesity or T2D in individuals from different geographic regions^10–12^. Furthermore, interventions known to improve metabolic health, such as exercise and bariatric surgery, were found to be associated with a decrease in adipose *Nnmt* expression and plasma MNA levels in obese individuals with insulin resistance or T2D^10^. Single-nucleotide polymorphisms of *Nnmt* have been associated with high BMI^13^, hyperhomocysteinemia^14^, non-alcoholic steatohepatitis^15^ or hyperlipidemia^16^.

Interventions to reduce *Nnmt* expression or NNMT enzymatic activity led to body fat loss and improved glucose handling in mice with diet-induced obesity and insulin resistance. This was seen with antisense oligonucleotides that reduced *Nnmt* expression in liver and WAT^7,9^ as well as with small-molecule NNMT inhibitors such as 6-methoxynicotinamide (JBSNF-000088)^6^ or 5-amino-1-methylquinoline^17^. Recently, chronic treatment with the latter was shown to accelerate weight loss and alleviate hepatic steatosis when switching from a Western diet to normal rodent chow^18^.

Additional NNMT inhibitors have recently been reported including other nicotinamide-related compounds such as 6-methylaminonicotinamide^19^, alpha-chloroacetamide-based compounds covalently binding to a cysteine residue in the SAM-binding pocket of the catalytic site^20^, macrocyclic peptides as allosteric NNMT inhibitors^21^, and bisubstrate-like inhibitors based on adenosyl scaffolds^22–26^, or 3-methyl-4-phenylpyrazole^27^ that occupy both the Nam and the SAM-binding pockets. The lack of cellular activity of these bisubstrate-like inhibitors was recently overcome by Iyamu et al.^28^ who generated SAM mimics extending into the nicotinamide binding pocket with nanomolar potencies in biochemical and micromolar potencies in cell assays. In-vivo activity of bisubstrate inhibitors still has to be explored.

Here we describe a novel series of tricyclic compounds that are potent and selective substrate-analog inhibitors of NNMT. They are orally bioavailable and reduce plasma and tissue MNA levels in vivo. Chronic administration of the lead compound, JBSNF-000028, to mice with diet-induced obesity limits weight gain and improves glucose handling. Activity in NNMT knockout animals demonstrates that the compounds provide metabolic benefit that goes beyond NNMT inhibition.

## Results

### Identification of small molecule Tricyclic Hit series

**Figure 1:**
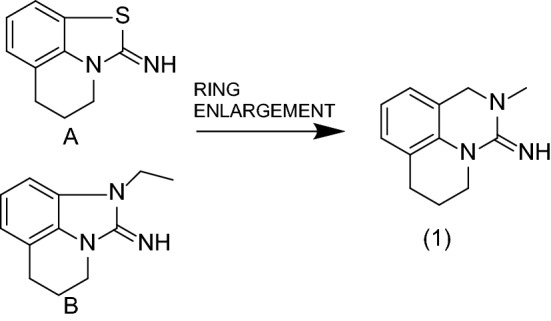
Identification of tricyclic lead (1) from screening hits (**A**) and (**B**). IC_50_ on human NNMT are 1.6 µM (**A**), 0.18 µM (**B**) and 0.13 µM (1).

The nicotinamide binding pocket of NNMT is an attractive target for small molecule inhibitors. Screening of Sanofi’s small molecule collection of NNMT inhibitors resulted in the identification of several structurally distinct hit series as reported in our earlier publication^27^. We focused our efforts on the tricyclic hit series from our HTS campaign with tricyclic structures such as hit compounds A and B shown in Fig. 1. Guided by structural biology data, we evaluated a ring enlargement strategy to improve the space filling in the nicotinamide binding pocket and synthesized derivative **(1)**. The improvement in inhibitory activity observed with **(1)** over the parent molecule prompted us to initiate a full lead optimization program on this tricyclic series.

While NNMT inhibitor **(1)** showed good activity on both human NNMT (hNNMT) and mouse NNMT (mNNMT) enzymes (IC_50_ values of 0.13 µM and 0.18 µM respectively), introduction of a fluorine atom in position 4 of the aromatic ring in **(2)** roughly retained mNNMT activity and delivered a small improvement on hNNMT potency. Substitution of **(1)** with two fluorine atoms generated the derivative **(3)** and decreased in vitro potency on both enzymes significantly (IC_50_ = 0.2 µM hNNMT and 1.1 µM mNNMT). We obtained similar results with derivative **(4)** after the replacement of the phenyl ring in **(1)** by a pyridine residue. Enlargement of the lower aliphatic ring was not tolerated and resulted in a complete loss of potency on hNNMT.

However, ring contraction of the lower aliphatic ring was tolerated and resulted in derivative **(6),** our most potent NNMT inhibitor on the human enzyme (IC_50_ = 0.034 µM) with good improvement in activity against the mouse enzyme (IC_50_ = 0.2 µM). In the next step of our optimization campaign, we checked the requirement for the amidine residue within our lead series. As can be seen from derivative **(7)**, the replacement of the amidine by urea rendered the compound inactive. Interestingly, NNMT inhibitors from our series with a 5-membered aliphatic ring system at the bottom were rather sensitive to substitution in the phenyl part of the molecule. Especially, activity against mNNMT was strongly reduced after the introduction of a single fluorine in the 4 position as demonstrated by derivative **(8)**. As observed earlier, the introduction of the second fluorine was detrimental for hNNMT potency **(9)**.

We also checked the impact of the size of the alkyl substituent on the amidine nitrogen in **(10)** and, by comparison with compound (2), confirmed N-methyl moiety as the one delivering highest in-vitro potencies on both human and mouse enzymes. The structure–activity relationship of compounds **(1)**–**(11)** is summarized in Table 1.

**Table 1:** SAR of the tricyclic compounds


CPD-ID	m	X	R_2_	R_3_	R_4_	Y	hNNMT IC_50_ (µM)	mNNMT IC_50_ (µM)
(1)	2	CH	H	Me	H	NH	0.13 ± 0.08	0.18 ± 0.01
(2)	2	CF	H	Me	H	NH	0.059 ± 0.004	0.33 ± 0.13
(3)	2	CF	F	Me	H	NH	0.19 ± 0.13	1.2 ± 0.75
(4)	2	N	H	Me	H	NH	0.36 ± 0.10	0.77 ± 0.04
(5)	3	CH	H	Me	H	NH	> 30	ND
(6)	1	CH	H	Me	H	NH	0.034 ± 0.001	0.20 ± 0.02
(7)	1	CH	H	Me	H	O	0.18 ± 0.02	0.38 ± 0.02
(8)	1	CF	H	Me	H	NH	0.039 ± 0.004	1.1 ± 0.5
(9)	1	CF	F	Me	H	NH	0.49 ± 0.04	> 3
(10)	1	CF	H	Et	H	NH	0.074 ± 0.030	> 3
(11)	1	CH	H	Me	F	NH	> 1	ND

### Heteroatom variables in the bottom ring

The six-membered aliphatic ring in **(1)** delivered a potent human and mouse NNMT inhibitor and we investigated the impact of introduction of an additional heteroatom into this region on in-vitro activity. We focussed our efforts on heteroatom introduction adjacent to the aromatic ring as shown in Table 2. Replacement of the -CH_2_- in this position by an oxygen atom **(12)** led to a slight loss of activity on hNNMT (IC_50_ = 0.18 µM) and mNNMT (IC_50_ = 0.38 µM). Choosing a sulfur atom instead of an oxygen **(13)** significantly reduced hNNMT activity (IC_50_ = 0.86 µM) without impacting activity on mNNMT. We then evaluated different nitrogen containing moieties in this position; only the N-cyano containing derivative **(14)** had activity on both enzymes in the sub-micromolar range and all other evaluated derivatives **(15)**–**(17)** remained inactive on the human enzyme. The introduction of fluorine atoms to **(12)** led to similar observations as described above for compounds **(2)**, **(3)** and **(8)**–**(10)**: With the introduction of one fluorine atom at position R_1_ in **(18)**, the activity on the mouse enzyme was compromised, and introduction of two fluorine atoms at R_1_ and R_2_ in **(19)** was not tolerated. In combination with X = NMe in **(20)**, we lost activity on the human enzyme already with only one fluorine atom. Further elongation of the substituent R_3_ resulted only in inactive derivatives **(21)** and **(22)**.

**Table 2:** Expansion of the SAR.


CPD-ID	X	R_1_	R_2_	R_3_	R_4_	hNNMT IC_50 (_µM)	mNNMT IC_50 (_µM)
(12)	O	H	H	Me	H	0.18 ± 0.02	0.38 ± 0.02
(13)	S	H	H	Me	H	0.86 ± 0.06	0.20 ± 0.02
(14)	–NCN	H	H	Me	H	0.25 ± 0.07	0.72 ± 0.07
(15)	–NAc	H	H	Me	H	> 30	ND
(16)	––NMe	H	H	Me	H	> 30	ND
(17)	–NCON(Me)_2_	H	H	Me	H	> 30	ND
(18)	O	F	H	Me	H	0.069 ± 0.036	> 3
(19)	O	F	F	Me	H	> 1	ND
(20)	––NMe	F	H	Me	H	> 3	ND
(21)	O	F	H	–CH_2_CF_3_	H	> 30	ND
(22)	O	H	H	–CH_2_CH_2_–(4F-phenyl)	H	> 30	ND

### Other modifications

**Figure 2:**
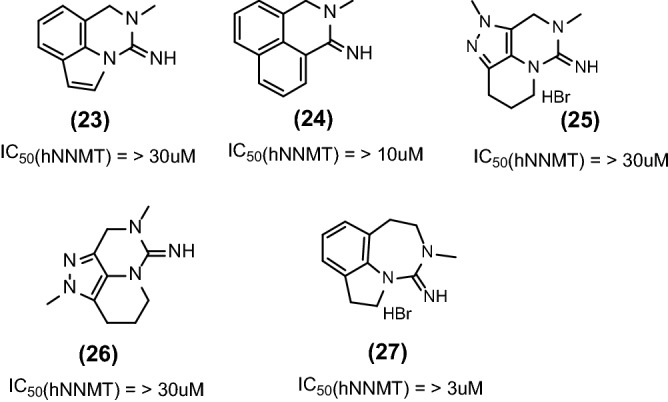
Other modifications in our lead series.

We investigated additional variations of **(1)** and **(6)** by focusing on the expansion of aromatic system as shown in Fig. 2, exemplified in **(23)** and **(24)**; replacement of the phenyl ring by 5-membered heteroaromatic rings as in **(25)** and **(26),** expansion of the amidine containing six-membered ring in **(27)**. All variations mentioned above were inactive on hNNMT enzyme and therefore we stopped these activities.

As next steps, cellular assays using the human U2OS cell line were carried out. This assay, described in^6^, measures inhibition of endogenous NNMT, without recombinant expression, by quantification of MNA via LC–MS/MS. Table 3 summarizes the results. The most active NNMT inhibitors **(1), (2)** and **(6)** in the enzymatic assay were also active in the cellular assay, with the strongest effect on MNA reduction after 1 h visible with inhibitor **(6)** that lowered endogenous MNA levels by 75%. EC_50_ values were higher by a factor of ~ 30 compared to IC_50_ values on the isolated recombinant hNNMT, probably reflecting low cell membrane permeability.

**Table 3:** Cell based NNMT inhibition and determination of MNA in cells (Target engagement).

Compound	Cell based inhibition in U2OS EC_50_ [µM]	% MNA reduction (time point [h])*
(1)	2.5 ± 0.3	30 (0.5)
(2)	2.2 ± 0.1	21 (0.5)
(3)	> 3	14 (1.0)
(6)	2.5 ± 0.3	75 (1.0)
(8)	1.1 ± 0.2	30
(9)	> 3	43 (0.25)
(13)	> 10	40 (1.0)
(18)	2.5 ± 0.2	30 (0.5)

*Time point after addition of compound at which maximal MNA reduction was observed relative to control without compound.

Based on the initial results from hNNMT, mNNMT enzymatic assays and the cell based assays, we progressed compound **(6)**, which we also label JBSNF-000028, towards a thorough assessment of in-vitro and physico-chemical properties. We also confirmed the binding mode of JBSNF-000028 in complex with hNNMT protein. Encouraged by the results obtained we moved forward into advanced in-vivo profiling and safety assessments.

### In-vitro profiling of JBSNF-000028

**Figure 3:**
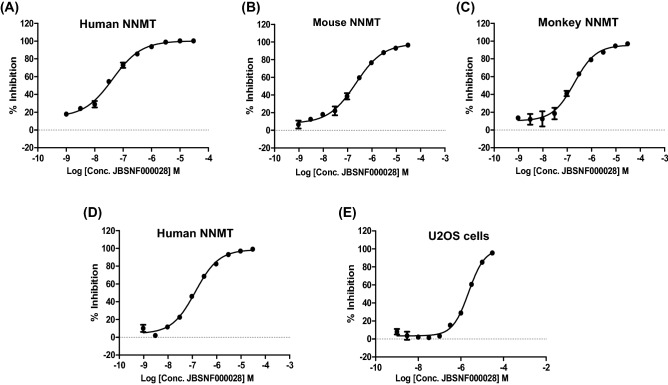
In-vitro activity of JBSNF-000028: Inhibition of recombinant (**A**) human, (**B**) mouse or (**C**) monkey NNMT as determined by fluorescence of an MNA derivative. (**D**) Inhibition of human NNMT as determined by LC/MS detection of MNA. (**E**) Inhibition of NNMT in U2OS cells as measured by LC/MS detection of MNA.

The inhibition of recombinant or intracellular NNMT by JBSNF-000028 is shown in Fig. 3. JBSNF-000028 inhibited the human NNMT (hNNMT), monkey NNMT (mkNNMT), and mouse NNMT (mNNMT) enzymatic activities with IC_50_ values of 0.033 µM, 0.19 µM and 0.21 µM, respectively (Fig. 3A–C). The activity of JBSNF-000028 against hNNMT was also confirmed by LCMS/MS detection method with an IC_50_ of 0.13 µM (Fig. 3D). Evaluation of endogenous cellular NNMT activity was undertaken in U2OS cells post 24 h treatment with JBSNF-000028 and the levels of MNA were assessed by LC–MS/MS to evaluate the inhibition levels of NNMT. The calculated EC_50_ value was 2.5 µM (Fig. 3E).

### Selectivity and safety profiling of JBSNF-000028

JBSNF-000028 was tested on a panel of diabetes/obesity targets from Cerep at 10 µM in duplicates. The panel consisted of APJ (apelin), TGR5, Bombesin receptor 3(BB3), GPR40-FFA1 (GPR40), GPR43-FFA2 (GPR43), Gpr120, Glucagon receptor, GIP receptor, GLP-1 receptor, Ghrelin receptor, Motilin, Orexin-OX1, RXR, VDR, KATP, PTH1, LXRα and GPR119. JBSNF-000028 was not active against any of these targets (Supplementary Table 1). Further, we tested JBSNF-00028 on a receptor and ion channel panel in Cerep at 10 µM in duplicates. No significant activity was found besides a 90% inhibition of monoamine oxidase A (MAO-A). JBSNF-000028 was tested for cytotoxicity using HepG2 cells. The cells were incubated with JBSNF-000028 at 10, 30 and 100 µM for 72 h and cytotoxicity was measured using the CellTiter-Glo assay kit. Cytotoxicity was not observed at any of the tested concentrations (data not shown). JBSNF-000028 was also tested on hERG assay (patch clamp), and NaV1.5 assay at 30 µM. No liability was found (< 30% inhibition @ 10 µM) (data not shown).

### Co-crystal structures of human NNMT with JBSNF-000028 and with JBSNF-000107

Thermal shift assay was performed to assess the binding of tricyclic compounds [**(6)** = JBSNF-000028, **(8)** = JBSNF-000107] to hNNMT prior to co-crystallization studies. Both the compounds were found to be tightly bound to human NNMT in presence of SAH (molar ratio of enzyme:SAH:compound ~ 1:5:10) with ~ 10 °C shift in melting temperature (Tm) whereas ~ 3 °C shift in Tm was noticed in the presence of SAM. (Supplementary Figure S1) Based on these results, crystallization was carried out with the hNNMT protein in the presence of JBSNF-000028 or JBSNF-000107 and SAH at molar ratio of enzyme:SAH:compound ~ 1:5:10. Co-crystals were obtained with JBSNF-000028 and JBSNF-000107 and X-ray crystal structures were determined in P1 space group at 2.61 Å and 2.71 Å resolution, respectively (PDBs: 7ET7, 7EU5). Data collection and refinement statistics are summarized in Supplementary Tables 2A, B.

The asymmetric unit contained four identical copies of human NNMT molecules (named A, B, C and D) and overlaid within 0.4 Å RMSD among each chain. For all further descriptions below, protein chain A is considered. Both co-crystal structures overlay within 0.2 Å RMSD. In the active site, demethylated cofactor, SAH is bound in the cradle formed by loops (141Cys-Asp-Val-Thr, 85Asp-Tyr-Ser, 63Gly-Ser-Gly, 163Thr-Leu-Cys, 69Tyr-Glu) originating from the Central domain. These loops are mentioned in the order of interaction from adenine, sugar and homocysteine. JBSNF-000028 or JBSNF-000107 is surrounded by Tyr-20, Tyr-24, Tyr-25 (from N-terminal domain), Leu-164, Asp-167, Ala-168 (from central domain), Ala-198, Ser-201, Tyr-203, Tyr-204, Ser-213, Tyr-242, Ala-247, Asn-249(from C-terminal domain) and SAH within 4.0 Å. Tricyclic compound (JBSNF-000028 (**Compound 6**) or JBSNF-000107 (**Compound 8**)) is planar in nature and bound below hairpin structural motif at the nicotinamide pocket and stacks between Tyr-204 (from Hairpin) and Leu-164 (from central domain). NH-group attached to saturated 6-membered ring interacts with side chain of Tyr-20 (from N-terminal helix), main chain carbonyl of Leu-164 and very proximal (~ 3.2 Å) to S-atom of SAH. Fluorine attached to unsaturated 6-membered ring interacts with side chain of Ser-201 (Supplementary Figs. S2A, B). The overlay with hit compounds A or B (Fig. 1) shows that compound A lacks the hydrogen bond interactions with both the sidechain of Tyr-20 and the backbone carbonyl oxygen of Leu-164 whereas compound B does not form an H-bond to Tyr-20 (Supplementary Fig. S2C, D). This could explain the lower potencies compared to JBSNF-000028 or JBSNF-000107.

### ADME, pharmacokinetic and safety profile of JBSNF-000028

The overall ADME, and safety profile of JBSNF-0000028 is summarized in Table 4. As can be expected from the chemical structure of JBSNF-0000028, the solubility in aqueous buffer is high with a value above 200 μM. The compound was tested using liver microsomes from different species and the metabolic stability of JBSNF-0000028 was found to be good across species. The compound did not inhibit any of the cytochrome P450 enzymes (CYP 3A4, 2D6, 2C19 and 2C9) at 20 µM.

**Table 4:** ADME, and safety profile of JBSNF-0000028.

Parameter	JBSNF-0000028
Solubility (μM)	> 200
Caco-2 permeability (nm/s)	1.05
Caco-2 Efflux ratio	4.90
Metabolic stability h/r/m/microsomes (% remaining)	90/95/86
CYP inhibition	> 20 µM
Ames test	Negative
Micronucleus test	Negative
Selectivity panel	Clean^a^

^a^Selectivity of JBSNF-0000028 against a panel of diverse targets is shown in Supplementary Table 1.

**Figure 4:**
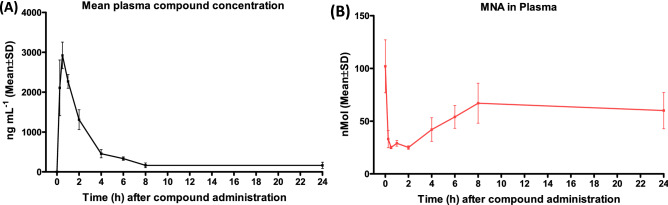
JBSNF-000028: Pharmacokinetics profile and target engagement. (**A**) Plasma concentration versus time profile of JBSNF-000028 in C57BL/6 mice. (**B**) Plasma levels of MNA confirming target engagement by JBSNF-000028 in C57BL/6 mice.

Pharmacokinetic analysis was carried out as described before^27^. The parameters of JBSNF-000028 from oral and intravenous pharmacokinetic studies are presented in Fig. 4 and Table 5. JBSNF-0000028 was quantifiable up to 8.0 and 10.0 h post intravenous and oral administration in mice respectively. Following intravenous administration of JBSNF-000028 at 1 mg/kg, the plasma concentrations decreased mono-exponentially. JBSNF-0000028 exhibited moderate clearance (CL) of 36.6 mL/min/kg, which is close to half of the hepatic blood flow (90 mL/min/kg) and high volume of distribution (Vd: 8.69 L/kg) in mice. The AUC_0-t_ (area under the plasma concentration–time curve from time zero to last time point) was found to be 446 ng × h/mL. The terminal half-life (T_½_) was found to be 1.77 h. Following oral administration of JBSNF-0000028 (10 mg/kg), maximum plasma concentration of C_max_: 452 ng/mL were attained at 1.00 h (T_max_) in all mice, suggesting that JBSNF-0000028 has a rapid absorption from the gastrointestinal tract. The T_½_ determined after oral administration was 2.36 h. AUC_0–t_ was 1369 ng × h/mL by oral route. Of note, compound exposure resulted in concomitant reduction in plasma MNA levels, demonstrating inhibition of NNMT catalytic activity (Fig. 4B).

**Table 5:** Pharmacokinetic parameters of JBSNF-0000028 in mice following intravenous (1 mg/kg) and oral administration (10 mg/kg).

PK parameters	*Intravenous*	Oral
Dose (mg/kg)	1	10
AUC_0–t_ (ng h/mL)	446	1369
C_0_/C_max_ (ng/mL)	432	452
T_max_ (h)	–	1.00
T_1/2_ (h)	1.77	2.36
T_last_ (h)	8.00	10.0
Cl (mL/min/kg)	36.6	–
Vd (L/kg)	8.69	–
F (%)	–	30

Compound JBSNF-0000028 was negative in the micronucleus test and the Ames mutagenicity test.

### JBSNF-000028 improves glucose and lipid handling in mice with diet-induced obesity (DIO)

Given the good drug like properties of JBSNF-000028 as ascertained by physicochemical, pharmacokinetics assessments, and target engagement experiments, compound JBSNF-000028 was investigated in efficacy studies using diet induced obesity (DIO), ob/ob and db/db diabetic mouse models using procedures as described before^27^.

**Figure 5:**
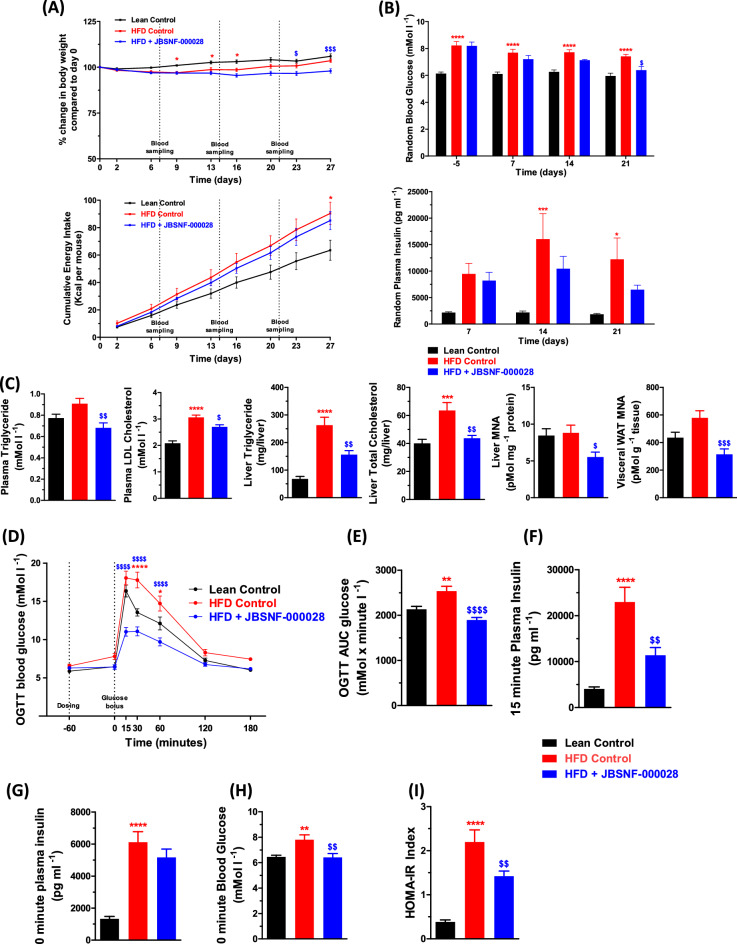
Efficacy of JBSNF-000028 in chronic DIO model. (**A**) Body weight changes (%) and cumulative energy intake in lean control animals and HFD fed animals treated with vehicle or JBSNF-000028 at 50 mg kg^−1^. (**B**) Fed blood glucose and fed plasma insulin profile of lean control animals and HFD fed animals treated with vehicle or JBSNF-000028 at 50 mg kg^−1^. (**C**) Plasma triglycerides, plasma LDL cholesterol, liver triglycerides, liver total cholesterol, MNA measurements in liver and visceral WAT samples of lean control animals and HFD fed animals treated with vehicle or JBSNF-000028 at 50 mg kg^−1^. (**D**) Oral glucose tolerance test (OGTT), (**E**) Area under the curve (AUC) for the OGTT. (**F**) Plasma insulin levels at t = 15 min. (**G**) Plasma insulin and (**H**) plasma glucose concentrations at t = 0 min. (**I**) HOMA-IR index. Data are presented as mean ± s.e.m. *p < 0.05, **p < 0.01, ***p < 0.0001 and ^****^p < 0.0001 when compared with lean Control and ^$^*p* < 0.05, ^$$^*p* < 0.01, ^$$$^*p* < 0.001, ^$$$$^*p* < 0.0001 when compared with HFD Control. Two way ANOVA followed by Bonferroni’s post-hoc test (**A**, **B**, **D**); One way ANOVA followed by Bonferroni’s post-hoc test (**C**, **E**–**I**).

The compound was tested at 50 mg kg^−1^, b.i.d, via oral route of administration. In the DIO model, the animals were obese with a high-fat diet (HFD) feeding for 14 weeks before treatment with JBSNF-000028. Animals remained on HFD throughout the treatment period. At the onset of treatment, body weights of animals on HFD were significantly higher (mean body weight of 48.3 g) as compared to age-matched control animals on normal rodent chow diet (mean body weight of 34.5 g). From day 23 of the treatment period, the JBSNF-000028 group showed statistically significant reduction in body weight (%) as compared to the vehicle treated group (Fig. 5A). The cumulative energy intake was comparable between the JBSNF-000028 treatment group and the vehicle treated group (Fig. 5A).

JBSNF-000028 treatment group showed statistically significant reduction in fed blood glucose on day 21 (*p* < 0.05) compared to vehicle control (Fig. 5B). Further, the treatment group also showed a trend to a reduction in fed plasma insulin that was not statistically significant on days 14 and 21 compared to HFD control (Fig. 5B). Reduction in MNA levels was observed in visceral WAT (*p* < 0.001) and Liver (*p* < 0.05) compared to HFD control (Fig. 5C). JBSNF-000028 at 50 mg kg^−1^ b.i.d. led to a statistically significant reduction in plasma triglyceride (*p* < 0.01), Plasma LDL cholesterol (*p* < 0.05), liver triglyceride (*p* < 0.01) and liver total cholesterol (*p* < 0.01) compared to HFD control (Fig. 5C).

Twice daily oral gavage administration of JBSNF-000028 at 50 mg kg^−1^ to DIO mice led to a marked improvement in oral glucose tolerance on day 28 (Fig. 5D) with glucose tolerance on JBSNF-000028 being normalized to that of the chow control group. Significantly lower AUC blood glucose (*p* < 0.0001) was observed in the compound treated group as compared to HFD control (Fig. 5E). Significantly lower blood glucose at 0 min during OGTT (*p* < 0.01) was observed in the compound treated group as compared to HFD control (Fig. 5H). JBSNF-000028 at 50 mg kg^-1^ showed statistically significant lower insulin (*p* < 0.01) at 15 min of OGTT compared to HFD control (Fig. 5F). JBSNF-000028 at 50 mg kg^−1^ showed no statistically significant difference in plasma insulin at 0 min of OGTT compared to HFD control (Fig. 5G). JBSNF-000028 at 50 mg kg^−1^ showed statistically significant improvement in HOMA-IR index (*p* < 0.01) compared to HFD control (Fig. 5I).

### Efficacy of JBSNF-000028 in a genetic model of diabetes (db/db mice)

**Figure 6:**
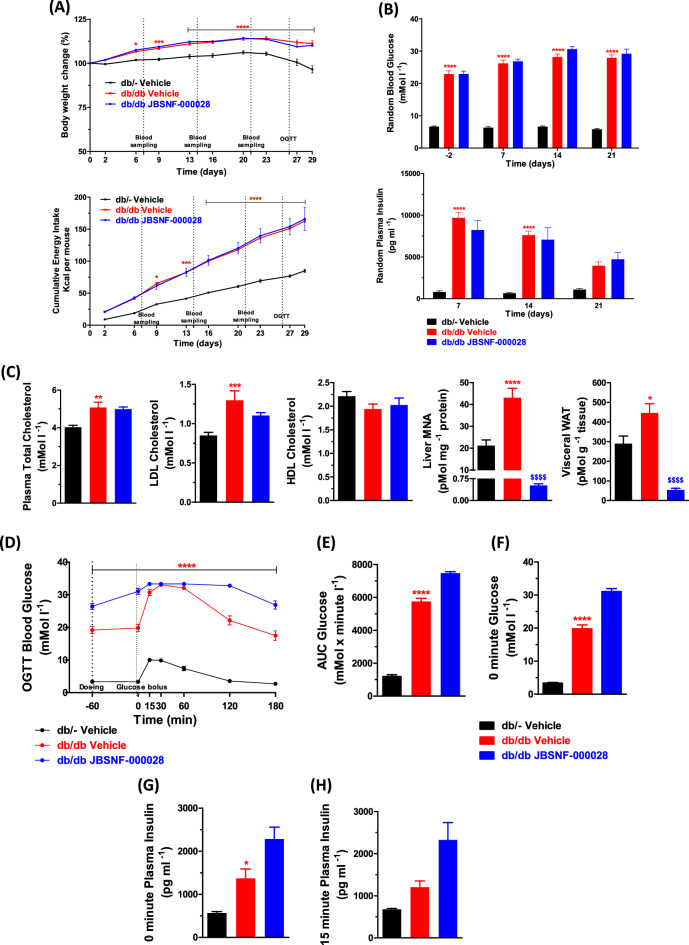
Efficacy of JBSNF-000028 in genetic model of type-2 diabetes (db/db mice). (**A**) Body weight changes (%) and cumulative energy intake in db/– control animals and db/db animals treated with vehicle or JBSNF-000028 at 50 mg kg^−1^. (**B**) Fed blood glucose and fed plasma insulin profile of db/– control animals and db/db animals treated with vehicle or JBSNF-000028 at 50 mg kg^−1^. **(C)** Plasma total cholesterol, plasma LDL cholesterol, plasma HDL cholesterol, MNA measurements in liver and visceral WAT samples of db/– control animals and db/db animals treated with vehicle or JBSNF-000028 at 50 mg kg^−1^. **(D)** OGTT profile, (**E**) Area under the curve (AUC), (**F**) plasma glucose and (**G**) plasma insulin levels at the time of the glucose bolus. (**H**) Plasma insulin levels at t = 15 min after the glucose bolus in the OGTT. Data are presented as mean ± s.e.m. *p < 0.05, **p < 0.01, ***p < 0.001 and ****p < 0.0001 when compared with db/– control and ^$$$$^*p* < 0.0001 when compared with db/db vehicle control. Two way ANOVA followed by Bonferroni’s post-hoc test (**A**, **B**, **D**); One way ANOVA followed by Bonferroni’s post-hoc test (**C**, **E**–**H**).

The studies were performed using the procedures described before^27^. The db/db animals were 8 weeks of age and had an average body weight of 43.35 ± 0.3 g at the onset of the four-week treatment with either vehicle or JBSNF-000028 as compared to age-matched lean db/– control animals that weighed 27.7 ± 0.3 g. Throughout the treatment period, the db/db animals in the vehicle control group and the JBSNF-000028 treatment group gained comparable weight and it was more than that gained by the db/– animals (Fig. 6A). The cumulative energy intake was comparable between the JBSNF-000028 treatment group and the vehicle treated group (Fig. 6A).

The db/db animals were overtly diabetic with fed glucose levels exceeding 20 mmol l^−1^ at the onset of treatment and remained so throughout the treatment period. The JBSNF-000028 treatment group showed no change in fed blood glucose levels on days 7, 14 and 21 as compared to vehicle control (Fig. 6B). Further, the treatment group showed no reduction in fed plasma insulin on days 7, 14 and 21 as compared to db/db control (Fig. 6B). Statistically significant reduction in MNA levels was observed in visceral WAT (*p* < 0.0001) and Liver (*p* < 0.0001) compared to db/db control (Fig. 6C). JBSNF-000028 at 50 mg kg^−1^ b.i.d. led to no significant change in plasma triglyceride, plasma LDL cholesterol and plasma HDL cholesterol as compared to db/db control (Fig. 6C). Upon twice daily oral gavage administration of JBSNF-000028 at 50 mg kg^−1^, there was no improvement in oral glucose tolerance on day 26 as compared to db/db control (Fig. 6D). In addition, administration of JBSNF-000028 showed no reduction in plasma insulin at 0 and 15 min during OGTT, and no reduction was seen in 0 min glucose during OGTT and AUC glucose during OGTT as compared to db/db control (Fig. 6E–H).

### Efficacy of JBSNF-000028 in genetic model of insulin resistance (ob/ob)

**Figure 7:**
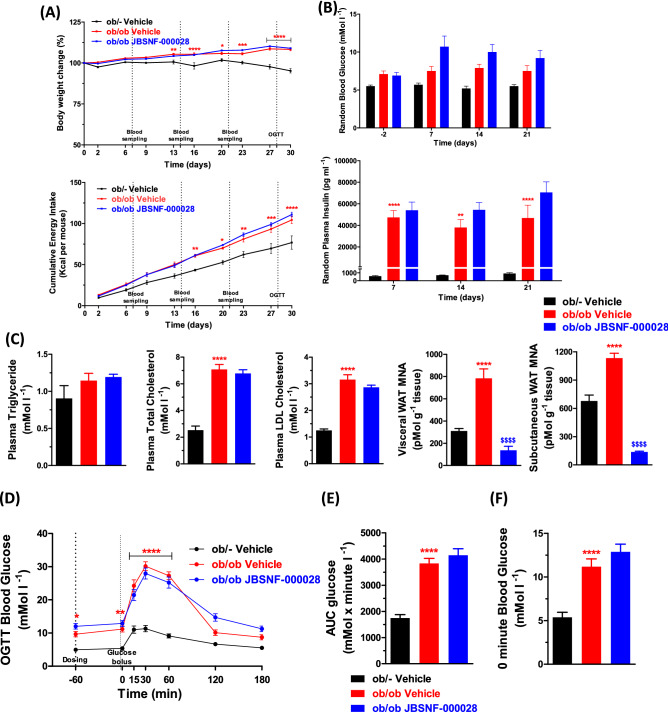
Efficacy of JBSNF-000028 in ob/ob model. (**A**) Body weight changes (%) and cumulative energy intake in ob/– control animals and ob/ob animals treated with vehicle or JBSNF-000028 at 50 mg kg^−1^. (**B**) Fed blood glucose and fed plasma insulin profile of ob/– control animals and ob/ob animals treated with vehicle or JBSNF-000028 at 50 mg kg^−1^. (**C**) Plasma triglycerides, plasma total cholesterol, plasma LDL cholesterol, MNA measurements in visceral WAT and s/c fat samples of ob/– control animals and ob/ob animals treated with vehicle or JBSNF-000028 at 50 mg kg^−1^. (**D**) OGTT profile, (**E**) Area under the curve (AUC), (**F**) plasma glucose levels at the time of the glucose bolus. Data are presented as mean ± s.e.m. *p < 0.05, **p < 0.01, ***p < 0.001 and ****p < 0.0001 when compared with ob/– control and ^$$$$^*p* < 0.0001 when compared with ob/ob vehicle control. Two way ANOVA followed by Bonferroni’s post-hoc test (**A**, **B**, **D**); One way ANOVA followed by Bonferroni’s post-hoc test (**C**, **E**, **F**).

The studies were performed using the procedures described before^27^. The ob/ob animals were 12 weeks of age at the onset of the 30-day treatment period with either vehicle or JBSNF-000028 (50 mg kg^−1^ b.i.d, p.o.) and had an average body weight of 53.7 ± 1.0 g whereas the age-matched lean ob/– control animals had an average body weight of 31.3 ± 0.7 g. Throughout the treatment period, the ob/ob animals in the vehicle control group and the JBSNF-000028 treatment group gained comparable weight and it was more than that of ob/– animals (Fig. 7A). The cumulative energy intake was comparable between the JBSNF-000028 treatment group and the vehicle treated group (Fig. 7A).

The JBSNF-000028 treatment group showed no reduction in fed blood glucose or insulin on days 7, 14 and 21 compared to vehicle control (Fig. 7B). Treatment with JBSNF-000028 for 30 days showed no improvement in plasma triglycerides, plasma total cholesterol, and plasma LDL cholesterol. However, as observed in the db/db study, treatment with JBSNF-000028 showed statistically significant reduction in MNA levels in visceral WAT and s/c fat (*p* < 0.0001), indicating target JBSNF-000028 target engagement (Fig. 7C). Twice daily oral gavage administration of JBSNF-000028 at 50 mg kg^−1^ to ob/ob mice did not show any improvement in fasting glucose levels or glucose tolerance on day 28 (Fig. 7D–F). Treatment with JBSNF-000028 for 30 days showed no improvement in plasma triglycerides, plasma total cholesterol, and plasma LDL cholesterol. However, as observed in the db/db study, treatment with JBSNF-000028 showed statistically significant reduction in MNA levels in visceral WAT and s/c fat (*p* < 0.0001) (Fig. 7C). The results from our in-vivo investigations are summarized in Table 6.

**Table 6:** Summary of in-vivo profiling in diabetic mice with JBSNF-0000028 after oral administration of 50 mg/kg b.i.d.

In-vivo results (50 mg/kg b.i.d.)	DIO mice	db/db Mice	ob/ob Mice
Reduction in plasma MNA levels	+	+	+
Reduction in body weight	+	−	−
Improvement in plasma insulin levels and glucose tolerance	+	−	−
Lower plasma and hepatic lipids	+	−	−

### Influence of JBSNF-000028 in NNMT knockout mice

**Figure 8:**
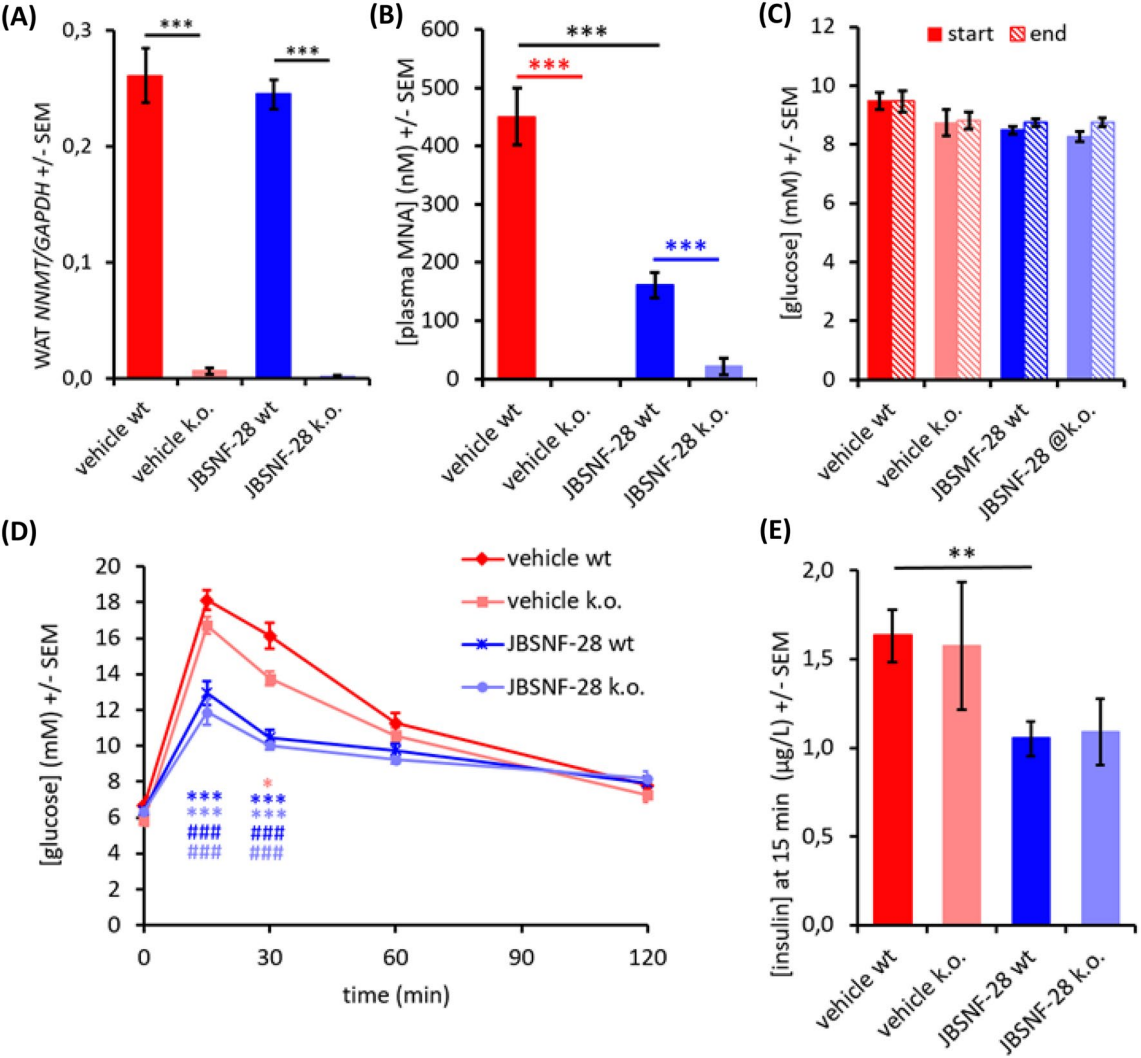
Effect of 4-w treatment with JBSNF-000028 (50 mg kg − 1 bid) in NNMT knockout animals on HFD. (**A**) *NNMT* expression in white adipose tissue of wild-type and NNMT knockout mice. (**B**) Plasma MNA concentrations in wild-type or knockout animals with or without treatment with JBSNF-000028. (**C**) Plasma glucose concentrations under fed conditions before (filled columns) and after (hatched columns) four weeks of treatment with JBSNF-000028. (**D**) Oral glucose tolerance test after four weeks of treatment with JBSNF-000028. (**E**) Plasma insulin levels 15 min after oral glucose bolus in the glucose tolerance test. *p < 0.05, **p < 0.01; ***p < 0.001 versus the other indicated column. (**D**) *p < 0.05 wild-type vs knockout (vehicle-treated), ***p < 0.001 for JBSNF-000028 treated wild-type (dark blue) or knockout (light blue) animals vs. vehicle-treated wild-type mice. ^###^*p* < 0.001 for JBSNF-000028 treated wild-type (dark blue) or knockout (light blue) animals versus vehicle-treated knockout mice.

Female wild-type and NNMT knockout animals were subjected to a high-fat for 18 weeks and then treated with either JBSNF-000028 (50 mg/kg bid by oral gavage) or vehicle for four weeks. As previously observed^6^, genetic deletion of *NNMT*, confirmed by lack of *NNMT* expression in white adipose tissue (Fig. 8A) led to loss of plasma MNA whereas treatment with JBSNF-000028 was associated with an about 70% reduction in plasma MNA (Fig. 8B). Whereas NNMT deletion or inhibition by JBSNF-00028 had no influence on fed plasma glucose (Fig. 8C), there was a marked improvement in oral glucose tolerance associated with a reduction in 15-min-insulin levels upon treatment with JBSNF-000028 (Fig. 8D,E). Of note, improvement in glucose tolerance and decrease in plasma insulin upon JBSNF-00028 was also observed in NNMT knockout animals, suggesting that JBSNF-000028 provides beneficial metabolic effects beyond NNMT inhibition.

## Discussion

Disorders of energy utilization and storage leading to metabolic syndrome are a predisposing factor for Type 2 Diabetes and liver diseases. NNMT is reported to be a regulator of adiposity and energy expenditure and is involved in modulating adipose SAM and NAD+, the metabolites for energy metabolism. NNMT is touted as an attractive target for developing small molecule inhibitors to treat obesity and type 2 diabetes. In this work, we describe a novel series of potent and selective tricyclic NNMT inhibitors that are active on the recombinant NNMT enzymes from several species and on the native, endogenous enzyme in human cells. With IC_50_ values in the double-digit nanomolar range and their low molecular weight of around 200 g/mol, these compounds have a high lipophilic ligand efficiency (LLE) indicating tight binding to the target as well as high drug-likeness^29^. The following Table 7 shows a direct comparison of LLE values between our JBSNF-000028 and NNMT inhibitors published earlier by the authors. Especially NNMT inhibitor (**29)** is only slightly above a LLE value of 2, which is commonly associated with HTS hits, whereas our JBSNF-000028 and **(28)** are in a more acceptable range for drug-like molecules. As described by Leeson and Springthorpe an ideal LLE value for an optimized drug candidate is in the area of 5–7 or higher^30^. However due to the higher in-vitro activity JBSNF-000028 is the most favorable choice for further profiling. The bioavailability of 30% found with JBSNF-000028 after oral dosing can be expected for a compound featuring a combination of high metabolic stability with rather low passive permeability as indicated by the Caco-2 value. However, due to the low molecular weight of JBSNF-000028 the compound is not limited to transcellular transport only as measured by the Caco-2 system, but can also benefit from paracellular transport ensuring sufficient plasma levels for robust pharmacodynamic effects^31^.

**Table 7:** Lipophilic Ligand Efficiencies (LLE) of published NNMT inhibitors compared to JBSNF-00028.

Compound	28^19^	29^27^	JBSNF-000028
Structure			
IC_50_ [µM]	0.59	0.26	0.033
clogP	− 0.46	3.34	1.06
LLE	6.7	3.2	6.4

In summary these compounds show a significant improvement over our earlier published NNMT inhibitors series^19,27^. We also learned that the structure–activity relationship around our NNMT inhibitors is rather narrow. Especially the need to maintain potency on human and mouse enzyme simultaneously turned out to be a limiting factor in compound selection. Based on its high activity on human, mouse and monkey NNMT enzyme and the good performance in our cellular experiments we identified JBSNF-000028 as the most promising candidate for in-depth in-vivo profiling and further pre-clinical assessments. JBSNF-000028 showed suitable pharmacokinetics for twice-daily oral administration and led to pronounced reduction in plasma and tissue 1-methylnicotinamide, indicating engagement of its target in vivo. The improvement in body weight, glucose metabolism and hepatic and plasma lipids in mice with diet-induced obesity confirmed previous findings by us and others^6,17,18^. Likewise, as previously shown for a structurally distinct series of NNMT inhibitors, JBSNF-000028 had little influence on body weight, glucose and lipids in ob/ob or db/db mice, two models of severe obesity driven by lack of leptin signaling that NNMT inhibition may not be strong enough to override^6^.

The improvement in glucose metabolism recapitulated similar effects seen in DIO-mice treated with an NNMT antisense oligonucleotide and in NNMT knockout mice on a high-fat diet^7,9^. In the latter, a markedly improved insulin sensitivity was observed in a hyperinsulinemic-euglycemic clamp experiment. DIO-mice treated with a NNMT antisense oligonucleotides showed less weight gain, reduced fed insulin levels and improved glucose tolerance, similar to our observations with JBSNF-000028 described here.

Surprisingly, the pronounced improvement in glucose tolerance upon treatment with JBSNF-00028 was observed in both wild-type and NNMT k.o mice with diet-induced obesity, pointing towards an additional glucose-normalizing effect of the compound beyond NNMT inhibition. While JBSNF-000028 did not show an activity against a broad panel of targets related to metabolism or safety, it was found to be an inhibitor of monoamine oxidase A (MAO-A) (Supplementary Table 2). Early studies from the 1960’s reported hypoglycemic activity of MAO inhibitors in patients with diabetes^32,33^, it is tempting to speculate that inhibition of MAO by JBSNF-00028 may, at least in part, be responsible for the improvement in glucose tolerance observed in both wild-type and NNMT knockout mice.

As for the mechanisms by which NNMT may influence glucose and energy metabolism are complex and incompletely understood. Several hypotheses have been put forward, such as the generation of reactive oxygen species^34^, modulation of cellular NAD+ levels or sirtuin activity^9,35^, reduced polyamine flux^9^, or a decrease in cellular methylation potential (e.g. via lowering the SAM/SAH ratio) and a resulting reduction in histone methylation^3,36^. Another role of NNMT that has been discussed in the setting of diet-induced obesity is lipotoxicity. Recently, Griffiths et al.^37^ demonstrated that NNMT upregulation via the mTORC1/ATF4 axis contributes to palmitate-induced cell death in hepatocytes that can be ameliorated by NNMT knockdown or inhibition. Similarly, in the setting of alcohol-induced fatty liver development, NNMT inhibition was observed to protect against hepatic steatosis and to be associated with activation of AMPK and suppression of hepatic de-novo lipogenesis^38^. To further complicate the situation, sex-specific differences have been observed for the response of NNMT knockout animals to dietary challenges, and phenotypes were dependent on the detailed diet composition (Western diet vs. High-fat diet^7^). Therefore, while protection from diet-induced obesity and impaired glucose metabolism has been consistently observed upon NNMT deletion, knockdown or inhibition, the molecular details and pathways mediating these effects remain to be further elucidated. This includes, for example, the role of NNMT in different tissues and cell types in determining the metabolic phenotype and in regulating the mechanisms described above. Studies analysing the effects of tissue-specific NNMT knockout are currently underway.

## Methods

### Compound synthesis

Compound syntheses and characterization are described in Supplementary Methods. NMR and high-resolution MS analytical data for compounds JBSNF-000028 and JBSNF-000107 are provided as Supplementary Figs. S3 and S4. Corresponding data for all other compounds described in this paper can be provided upon request to the authors.

### Cloning, expression and purification of human NNMT protein

Full length human nicotinamide N-methyl transferase (NNMT) (Uniprot ID–P40261) encompassing residues 1–264 was cloned into pET28a, in between NdeI and XhoI restriction sites, introducing an amino terminal His tag (HHHHHH) with a thrombin cleavage site (LVPRGS). The recombinant protein was expressed in E.coli BL21 DE3 cells. Following an induction with 0.5 mM IPTG when O.D600 reached 0.6, the cells were grown overnight at 18 °C and then harvested.

The harvested cells were resuspended in lysis buffer (20 mM HEPES pH 7.5, 500 mM NaCl, 10 mM imidazole, 5% glycerol, 1 mM TCEP, 1 mM PMSF, protease inhibitor and 0.1% lysozyme) and subjected to Ni–NTA chromatography using an imidazole gradient. The eluted fractions were pooled and applied to a superdex200 column pre-equilibrated with 20 mM HEPES pH 7.5, 50 mM NaCl, 5% glycerol, 0.5 mM Benzamidine HCl and 1 mM TCEP. Eluted fractions showing a single protein were combined and concentrated to 12 mg/mL in presence of SAH @ 1:5 protein-SAH molar ratio.

### Thermal shift assay

Thermal shift assay experiments helped in finalizing co-factor for crystallization experiments. All experiments planned as per the previously established protocols1. 10uM JBSFN-107 in presence of 5uM SAH recorded Tm shift up to 11 °C, whereas in presence of 5uM SAM it is measured up to 3 °C.

### Crystallization and data collection

Protein purified with SAH incubated with JBSNF-107 at 1:10 protein-compound molar ratio based on thermal shift assay results. Protein—compound incubation done for 3 h at 4 °C. Crystals obtained in sitting drop vapor diffusion method with the reservoir containing 0.2 M Sodium chloride, 0.1 M BIS–TRIS pH 5.2, 24%(w/v) PEG 3350. Crystals grew better in less than 2 weeks and data collected upto 2.7 Å at Australian Synchrotron beamLines. 18% Glycerol used as cryo solution.

### Structure determination and refinement

Structure determination and refinement were carried out using procedures described before (6). Structure solutions were obtained for both using MOLREP program of CCP4 Suite version 5.0.2^39^ with 3ROD as the start model. Model correction and inspection of electron density map were done using COOT (v 0.5.2)^40^, refinement with REFMAC5 program^41^ and Ligand fitted using AFITT (v1.3.2)^42^ program. R-factors for human NNMT complexes converged to Rwork(%)/Rfree(%) 22.4/28.6 and 20.7/23.7 respectively. Ramachandran plot calculated using PROCHECK^43^ confirmed good stereochemistry. The atomic coordinates and structure factors for human NNMT—JBSNF-000028 and human NNMT—JBSNF-107 complexes have been deposited in the Protein Data Bank with the accession numbers 7ET7 and 7EU5 respectively. All the crystal structure figures were generated using PyMOL^44^.

### Fluorescence based NNMT enzymatic assay

NNMT activity was measured by a fluorescence based assay as previously described^6^. Briefly, Inhibitors were screened using human, mouse and monkey NNMT enzymes at different concentrations. The final assay reaction mixture contained a buffer of 100 mM Tris Hcl pH 7.5, 0.04% BSA, 2 mM Dithiothreitol and 1% DMSO. A fluorescent product 2, 7- naphthyridine was measured in a Tecan reader with excitation at 375 nm and emission at 430 nm. The IC_50_ values were determined by fitting the inhibition curves (percent inhibition versus inhibitor concentration) using a four parametric sigmoidal dose response in graph pad prism.

### LC–MS/MS based detection of human NNMT enzymatic assay

MNA formed in the human NNMT reaction was measured using LC–MS/MS method as described before^6^. Briefly, different concentrations of the inhibitors were pre-incubated along with 5 ng/well human NNMT enzyme and the reaction was initiated by addition of SAM and nicotinamide mixture at 7 µM, 20 µM respectively. The final assay reaction mixture contained a buffer of 100 mM Tris HCl pH 7.5, 0.04% BSA, 2 mM dithiothreitol and 1% DMSO and the analysis was carried out using LC–MS/MS. The IC_50_ values were determined by fitting the inhibition curves (percent inhibition versus inhibitor concentration) using a four parametric sigmoidal dose response graph pad prism.

### Cell based U2OS assay

The cell based assay with Human bone osteosarcoma (U2OS) cell line was performed using procedures described before (6). Briefly, the cells were seeded into a 96 well cell culture plate followed by incubation for 24 h at 37 °C, 5% CO2, and 95% humidity. Cell culture media was replaced with 100 µl of medium/ inhibitor mixture at different concentrations and incubated for another 24 h. The cells were processed and 150 µL of supernatant was transferred into 96 well plate (Costar 3364) and analyzed by LC–MS/MS. The IC_50_ values were determined by fitting the inhibition curves (percent inhibition versus inhibitor concentration) using a four parametric sigmoidal dose response graph pad prism.

### Cytotoxicity assay

The cytotoxicity assay was carried out using procedures described before (6). Briefly, Human liver cancer (HepG2) cells cultured in DMEM Glut Max growth media containing 10% HI FBS and 1% Pen-Strep (filter sterilized). Cells were seeded into a 96 well opaque-walled multi well plate followed by Incubation overnight at 37 °C, 5% CO_2_, 95% humidity. Cell culture media was replaced with 100 µL of medium/ inhibitor mixture (Containing 0.5% DMSO) along with controls and incubated for 48 or 72 h at 37 °C, 5% CO_2_, 95% humidity. Cell Titer-Glo^®^ Reagent (Promega) was added to all the wells containing media in 1:1 ratio and the plates were processed for luminescence measurement using Victor or Top Count Luminescence counter. The cell viability over DMSO control was calculated.

### Animal studies

All the animal experiments were approved by Institutional Animal Ethical Committee (IAEC) of Jubilant Biosys nominated by Committee for the Purpose of Control and Supervision of Experiments on Animals (CPCSEA) [Registration No: 1026/PO/RcBi/S/07/CPCSEA]. IAEC number for mouse IV/ PO PK is IAEC/JDC/2015/72. IAEC number for DIO, ob/ob and db/db mouse studies is IAEC/JDC/2015/61. Animal studies were conducted out according to the ARRIVE guidelines^45^.

#### Pharmacokinetic studies

The pharmacokinetic studies were carried out using procedures described before (6). Briefly, the animals were divided into two groups (n = 12/group). Group A animals (25–30 g) received JBSNF-0000028 orally as a suspension formulation [prepared using 0.5% Tween-80 and 0.5% ethyl cellulose in water] at 10 mg/kg (strength: 1.0 mg/mL; dose volume: 10 mL/kg), whereas Group B animals (27–32 g) received JBSNF-0000028 intravenously formulation [prepared using 5% DMSO and 95% of 20% Captisol in Milli-Q water]; strength: 0.1 mg/mL; dose volume: 10 mL/kg) at 1 mg/kg dose. Post-dosing serial blood samples (50 μL) were collected through tail vein into polypropylene tubes containing K_2_EDTA solution as an anti-coagulant at 0.25, 0.5, 1, 2, 4, 8, 10, 12, and 24 h (for oral route) and 0.12, 0.25, 0.5, 1, 2, 4, 8, and 24 h (for intravenous route). Plasma was harvested by centrifuging the blood using Biofuge (Hereaus, Germany) at 1760 g for 5 min and stored frozen at − 80 ± 10 °C until further analysis. Animals were allowed to access feed 2 h post-dosing.

#### Target engagement and efficacy studies

Animals: Male C57BL6/N (Vivo Bio Tech Ltd, India), ob/ob (B6.V-Lepob/OlaHsd) and its lean control (Envigo laboratories Inc, USA) and db/db (BKS.Cg-+Leprdb/+Leprdb/OlaHsd) and its lean control (Envigo laboratories Inc, USA) were housed in individually ventilated cages (n = 5/cage) under standard laboratory conditions. The study room environment was maintained at a relative humidity of 40–70% and temperature of 21–24 °C. The animal rooms were maintained under 12 h light and dark cycle. Animals were provided with ad libitum access to water and food (except where noted otherwise, e.g. during fasting prior for glucose tolerance tests, fasting prior to terminal sacrifice). The acclimatization process for animals were completed 1 week before initiation of studies. C57BL6/N mice were maintained on rodent chow diet (Teklad; Harlan) or HFD (60% Kcal; Research diet) for 14 weeks before study initiation and throughout the study period for Diet induced obesity (DIO) mice study. Rodent chow diet (Teklad; Harlan) was provided to ob/ob and db/db mice and their lean controls^6^.

##### Formulation preparation

Formulations were prepared in mortar and pestle using Tween 80 and 0.5% HEC. The content was transferred into polypropylene tube. The final volume was made up by adding appropriate quantity of 0.5% HEC under constant stirring.

##### Target engagement study

The source of animals for these studies was Male C57BL6/N mice (Vivo Bio Tech Ltd, India). On the study day, 24 animals were dosed with JBSNF-000028, 50 mg kg^−1^, p.o. and 3 animals per time point were bled and sacrificed after 0.25, 0.5, 1, 2, 4, 6, 8 and 24 h of dosing. 3 animals were bled and sacrificed at 0 min (no treatment). Plasma was collected and stored at − 80 ± 10 °C until analysis. The Target engagement studies were carried out as described previously^6^.

##### Efficacy study

Lean control + Vehicle and HFD + Vehicle Control groups (G1 and G2) mice were administered with vehicle. Dose formulations of HFD + JBSNF-000028, 50 mg kg^−1^, po, bid were administered to G3 mice. All the doses were given at dose volume of 5 mL kg^−1^ body weight. Similar dosing regimen was followed for ob/ob mice and db/db mice studies. Most recent body weight of individual animal was taken into account for calculation of dose volume. All animals were observed for mortality/morbidity during the study period. Daily cage side observations of each animal were performed for visible clinical signs. Twice weekly during the study period individual animal body weights were recorded. Ob/ob mice, db/db mice and DIO mice were 12 weeks, 8 weeks and 20 weeks of age respectively at the start of the study. In each study, Control group comprising non-diabetic lean mice had received vehicle (0.5% w/v HEC and 0.5% v/v Tween 80) and obese or diabetic mice were randomly assigned to two groups based on body weight and nonfasted glucose which received either vehicle or JBSNF-000028, 50 mg kg^−1^, po, bid for 30 days. During the study period twice weekly individual animal body weights, water and food consumption were recorded. On day 7, 14 and day 21 post treatment insulin and fed blood glucose were measured^6^.

#### N-methylnicotinamide (MNA) bioanalytical analysis

*Sample preparation* A simple protein precipitation extraction method was followed for extraction of N-methylnicotinamide from plasma and various tissue homogenate samples. The tissue samples were homogenized with 1:3 proportions of phosphate buffer saline (PBS) and this homogenate was used for N-Methylnicotinamide determinations. To an aliquot of ~ 50 μL of plasma/liver homogenate/ adipose tissue homogenate samples ~ 300 µl of acetonitrile containing IS (d_4_-MNA) solution was added, followed by vortex for ~ 3 min and centrifuged for 10 min at 14,000 rpm. The supernatant was separated and transferred to pre-labeled HPLC vials and analyzed by LC–MS/MS system.

##### Analysis

Samples for methylated JBSNF-000028 were analyzed employing a fit-for-purpose LC–MS/MS method. PE-Sciex API-5500 triple quadrupole (PerkinElmerSciex Instruments, Boston, MA) mass spectrometer was employed for this analysis. Samples (2 µL) were injected onto Atlantis dC18 (50 × 4.6 mm, 3 µm; Waters, Milford, MA, USA) column connected to Shimadzu VP (Shimadzu, Japan) LC system. The gradient mobile phase mixture of 0.2% formic acid and acetonitrile was delivered at a flow rate of 0.9 mL min^−1^ into the mass spectrometer electrospray ionization (ESI) chamber. Quantitation was achieved by MS/MS detection in positive ion mode for methylated JBSNF-000028 and internal standard (Tolbutamide). Detection of the ions was performed in the multiple reaction monitoring (MRM) mode, monitoring the transition of the m/z 168 precursor ion to the m/z 128.8, product ion for MNA and m/z 271 precursor ion to the m/z 91 product ion for tolbutamide (internal standard). The retention times of MNA and tolbutamide were 1.45 min and 3.09 min, respectively.

#### OGTT

On day 28 of treatment, the effect of JBSNF-000028 on glucose tolerance was measured in an oral glucose tolerance test (OGTT), in DIO mice, ob/ob mice and on day 26 of treatment for db/db mice. The procedure for OGTT were carried out as described previously^6^.

#### Study termination

Animals were kept on fasting for 4 h and sacrificed by CO_2_ asphyxiation on day 30. All the sample processing at study termination were carried out as described previously^6^.

#### Biochemical analysis

Plasma insulin levels were measured using an ELISA kit (Mercodia AB, Uppsala, Sweden) as per kit insert. Plasma HDL, LDL, TG and TC were measured by colorimetric methods using commercially available Randox assay kits (Randox Lab., Ltd, UK). Whole blood glucose was measured using Glucometer (Contur, Bayer)^6^.

### Statistical analysis

Data are reported as the mean ± SEM throughout, and p value less than 0.05 was used as the threshold for statistical significance. Graphpad Prism-5 software was used for all statistical analysis. Two way ANOVA followed by Bonferroni post hoc test was used for Change in body weight, weekly fed blood glucose, cumulative energy intake, weekly fed plasma insulin and OGTT. One way ANOVA followed by Bonferroni post hoc test was used for analysis of 0 min blood glucose, AUC blood glucose in OGTT, 0 and 15 min plasma insulin, MNA in plasma, HOMA-IR index, MNA in visceral WAT, MNA in S/C WAT and MNA in liver.

### DIO studies in WT and NNMT KO animals

Whole-body NNMT knockout mice were generated by crossing NNMT fl/fl mice (C57BL/6 background) with ZP3-cre mice (C57BL/6, Jackson Laboratories) as previously described^7^. Studies were performed in accordance to the German Animal Protection Law, as well as according to international animal welfare legislation and rules. Female wild-type and NNMT knockout mice were fed a high-fat diet (Ssniff HFD adjusted TD.97366) for 18 weeks and then treated with either JBSNF-000028 (50 mg/kg bid by oral gavage) or vehicle for four weeks (n = 9–10 per group). Throughout the study period, mice were housed at 23 °C on a 12 h:12 h light dark cycle (light on at 06:00 AM). Food and water were accessible ad libitum. An oral glucose tolerance test was performed on treatment day 25: After an overnight fast, JBSNF-000028 (50 mg kg^−1^) or vehicle (0.5% HEC + 0.5% Tween80) was administered 60 min before an oral glucose bolus (2 g kg^−1^), by oral gavage. For blood glucose analysis, blood was collected from the tail of conscious mice at time points 15, 30, 60 and 120 min after the glucose bolus. At study end, they were sacrificed by terminal bleeding from Vena cava caudalis under deep isoflurane anesthesia.

## Supplementary Information

Supplementary Information 1.

Supplementary Information 2.

## Data Availability

The datasets used and analysed during this study are available from the corresponding authors upon reasonable request.
