# Supplementary material for: Novel tricyclic small molecule inhibitors of Nicotinamide N-methyltransferase for the treatment of metabolic disorders
Source: Sci Rep. 2022 Sep 14;12:15440. doi: 10.1038/s41598-022-19634-2 (PMC9474883; doi:10.1038/s41598-022-19634-2)
Supplement: Supplementary file 1 — Supplementary Information 1. [file 41598_2022_19634_MOESM1_ESM.pdf]

Supplementary Tables and Figures to

Novel tricyclic small molecule inhibitors of Nicotinamide N-methyltransferase for the treatment of metabolic disorders

Sven Ruf[1#], Sridharan Rajagopal[2#], Sanjay Venkatachalapathi Kadnur[3], Mahanandeesha S Hallur[3], Shilpa Rani[3], Rajendra Kristam[3], Srinivasan Swaminathan[3], Bharat Ravindra Zope[3], Pavan Kumar Gondrala[3], Indu Swamy[3], Rama Kishore Putta V P[3], Saravanan Kandan[3], Gernot Zech[1], Herman Schreuder[1], Christine Rudolph[1], Ralf Elvert[1,4], Joerg Czech[1], Swarnakumari Birudukota[3], Amir Siddiqui M[3], Niranjan Naranapura Anand[3], Vishal Subhash Mane[3], Sreekanth Dittakavi[3], Juluri Suresh[3], Ramachandraiah Gosu[3], Mullangi Ramesh[3], Takeshi Yura[3], Saravanakumar Dhakshinamoorthy[3*], Aimo Kannt[1,5*]

[1]*Sanofi-Aventis Deutschland GmbH, R&D, Integrated Drug Discovery, Industriepark Hoechst, 65926 Frankfurt am Main, Germany*
[2] *Jubilant Therapeutics India Ltd, Bangalore 560022*
[3]*Jubilant Biosys Ltd, Bangalore-560022, India*
[4]*Evotec GmbH, Marie-Curie-Straße 7, 37079 Göttingen, Germany*
[5]*Fraunhofer-Institute for Translational Medicine and Pharmacology ITMP, Theodor-Stern-Kai 7, 60596 Frankfurt am Main, Germany*
[#] *joint first authors*
[*] *corresponding authors*
Saravanakumar Dhakshinamoorthy (Saravanakumar.Dhakshinamoorthy@jubilantbiosys.com)
Aimo Kannt (aimo.kannt@itmp.fraunhofer.de)

Supplementary Table 1

Cerep Panel-33 Activity-JBSNF-000028

Target	% Inhibition at 10µM
glycine (strychnine-sensitive) (antagonist radioligand)	NA
N neuronal a4b2 (h) (agonist radioligand)	NA
PCP (antagonist radioligand)	NA
Ca2+ channel (L, dihydropyridine site) (antagonist radioligand)	NA
KV channel (antagonist radioligand)	NA
SKCa channel (antagonist radioligand)	NA
Cl- channel (GABA-gated) (antagonist radioligand)	NA
norepinephrine transporter (h) (antagonist radioligand)	NA
dopamine transporter (h) (antagonist radioligand)	NA
A1 (h) (agonist effect)	12
A2A (agonist effect)	NA
Thiopurine S-methyltransferase (h)	9
a1A (h) (agonist effect)	NA
a2A (h) (agonist effect)	8
M1 (h) (agonist effect)	NA
M2 (h) (agonist effect)	NA
M3 (h) (agonist effect)	NA

Target	% Inhibition at 10µM
5-HT2A (h) (agonist effect)	NA
5-HT2B (h) (agonist effect)	19
β1 (h) (agonist effect)	NA
β2 (h) (agonist effect)	NA
PDE3A (h)	5
acetylcholinesterase (h)	7
Phenylethanolamine N-methyltransferase (h)	28
MAO-A (h)	90
CB1 (h) (agonist effect)	20
CB2 (h) (agonist effect)	NA
D1 (h) (agonist effect)	NA
D2S (h) (agonist effect)	NA
H1 (h) (agonist effect)	NA
H2 (h) (agonist effect)	NA
m (MOP) (h) (agonist effect)	12
5-HT1A (h) (agonist effect)	NA

Supplementary Table 1, continued

Diabetic Panel activity JBSNF-00028

Target	% inhibition at 10uM
APJ (apelin) (agonist effect) cellular assay	NA
TGR5 (agonist effect) cellular assay	NA
Bombesin receptor 3(BB3) (agonist effect) cellular assay	NA
GPR40-FFA1 (GPR40) (agonist effect) cellular assay	NA
GPR43- FFA2 (GPR43) (agonist effect) cellular assay	NA
Gpr120- (agonist effect) cellular assay	NA
Glucagon- Glucagon GIP (agonist effect) cellular assay	NA
GLP-1 agonist effect cellular assay	NA
Ghrelin-Ghrelin (agonist effect) cellular assay	NA
Motilin agonist cellular assay	NA
Orexin- OX1 cellular agonist assay	NA
GPR119 agonist cellular assay	NA
PPARα agonist cellular assay	NA
PPARβ agonist cellular assay	NA
PPARγ agonist cellular assay	NA
PXR agonist cellular assay	NA
RXR alpha binding assay	NA
VDR agonist cellular assay	NA
KATP antagonist radioligand	NA
PTH1 agonist cellular assay	2.3
LXRα agonist cellular assay	NA

Supplementary Figure S1

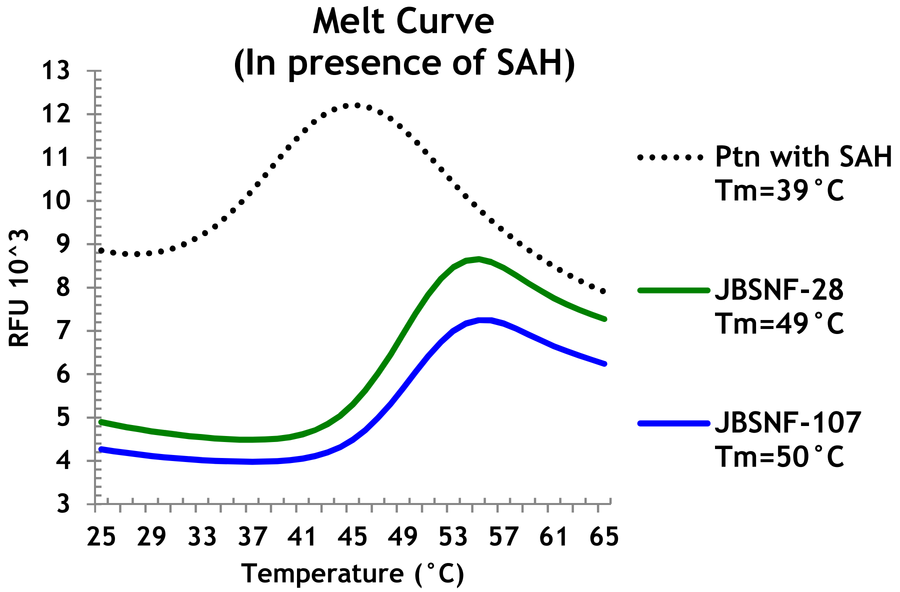

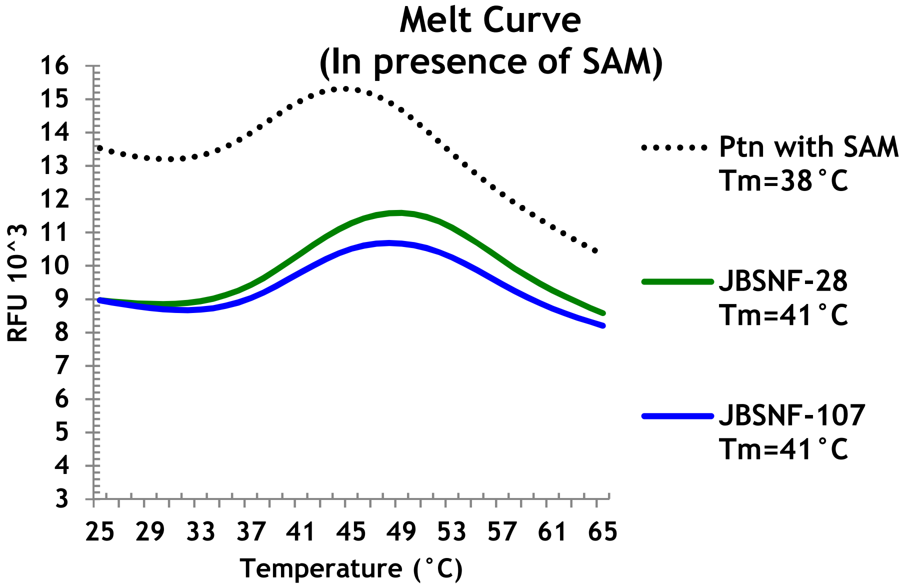

Inference:

JBSNF-28* & JBSNF-107* binds tighter to hNNMT in presence of SAH (ΔTm ~10 °C)

JBSNF-28* & JBSNF-107* binds weaker to hNNMT in presence of SAM (ΔTm ~3 °C)

*JBSNF-28 = JNSBF-000028 = (**6**); JBSNF-107 = JBSNF-000107 = (**8**)

Experiment Conditions

Data: In Triplicate

Dye: 5x SYPRO orange

Reaction Volume: 50uL

Protein: 1uM (hNNMT)

SAH/SAM: 5uM

Cmpd: 10uM

Supplementary Table 2A: Co-crystal structure with JBSNF-000028 (pdb code 7ET7)

Data Collection Statistics (Australian Synchrotron – MX2 beam lines; Data processing using Aimless program)		
Number of frames		360
Oscillation width (°)		0.5
Exposure per frame (sec)		1
Distance (mm)		400
Space Group		P1
Resolution Range (Å)		40.42–2.61 (2.73 – 2.61)
Unit Cell constants	(Å)	a= 60.93 , b= 62.86, c= 75.60
	(°)	α=108.03, β=103.54, γ=104.25
Rmerge (%)		10.6 (46.5)
Completeness (%)		97.5 (93.1)
<I>/σ(<I>)		3.3 (1.1)
Average redundancy		2.0 (1.9)

Refinement Statistics		
No of molecules in asymmetric unit		4
Start model PDB-id		JBSNF107 model
N-term amino acid		A-5(Phe), B-5(Phe), C-5(Phe), D-5(Phe)
C-term amino acid		A-260(Leu), B-260(Leu), C-260(Leu), D-261(Ser)
Final R-factor (Rfree)		0.223 (0.287)
Ramachandran Statistics (%)	Core	90.5
	Allowed	8.8
	Generous	0.3
	Disallowed	0.3
No of water molecules		31
Ligand bound status		Yes (A,B.C,D – Chains)
Number of Metal atoms		Nil
Other solvents		Nil

Supplementary Table 2B: Co-crystal structure with JBSNF-000107 (pdb code: 7EU5)

Data Collection Statistics (Australian Synchrotron – MX2 beam lines; Data processing using Aimless program)		
Number of frames		180
Oscillation width (˚)		1
Exposure per frame (sec)		1
Distance (mm)		400
Space Group		P1
Resolution Range (Å)		48.73–2.71 (2.84 – 2.71)
Unit Cell constants	(Å)	a= 60.74 , b= 62.54, c= 71.49
	(˚)	α=94.21, β=103.00, γ=103.65
Rmerge (%)		19.0 (58.5)
Completeness (%)		94.6 (74.2)
<I>/σ(<I>)		3.1 (1)
Average redundancy		1.9 (1.9)

Refinement Statistics		
No of molecules in asymmetric unit		4
Start model PDB-id		3ROD
N-term amino acid		A-5(Phe), B-6(Thr), C-5(Phe), D-5(Phe)
C-term amino acid		A-260(Leu), B-260(Leu), C-261(Ser), D-261(Ser)
Final R-factor (Rfree)		0.215 (0.285)
Ramachandran Statistics (%)	Core	91
	Allowed	9
	Generous	0
	Disallowed	0
No of water molecules		43
Ligand bound status		Yes
Number of Metal atoms		Nil
Other solvents		Nil

Supplementary figure S2A

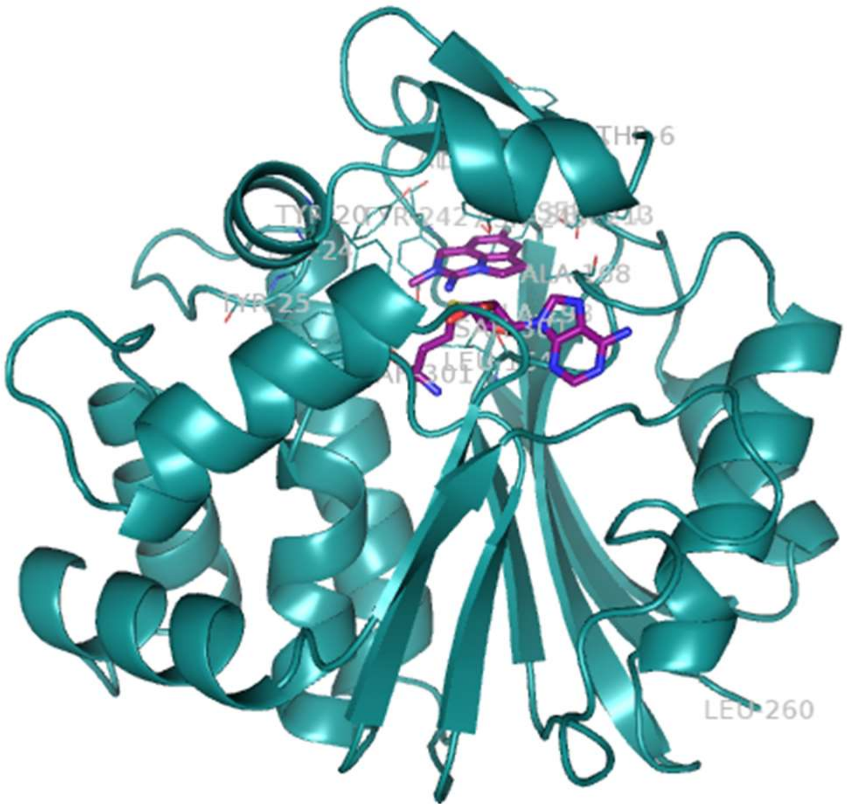

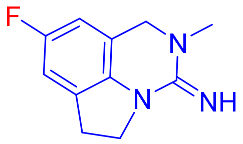

(8) (JBSNF-000107)

hNNMT IC_{50} = 0.038μM

hNNMT -JBSNF-000107
Complex Crystal Structure
(Representative Chain)

Supplementary figure S2B

hNNMT-JBSNF-000028 (in magenta; 2.61Å)
hNNMT-JBSNF-000107 (in pink; 2.71Å)

Cmpd & SAH in ball & stick model
Residues within 4Å of cmpd are shown in lines

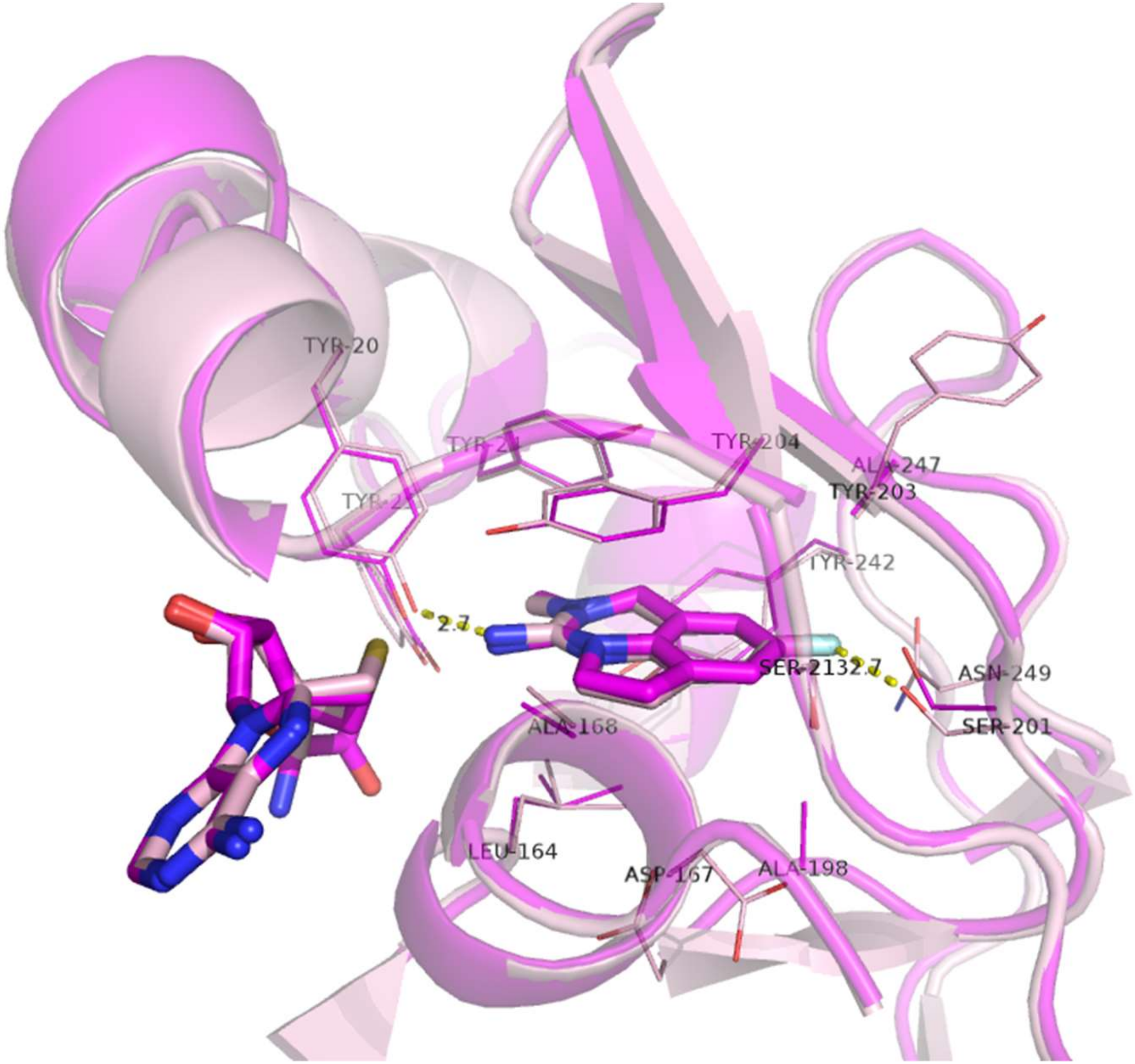

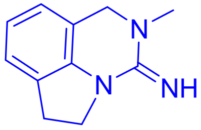

(6) (JBSNF-000028)
hNNMT IC_{50} = 0.033μM

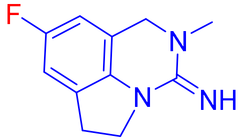

(8) (JBSNF-000107)
hNNMT IC_{50} = 0.038μM

Supplementary figure S2C

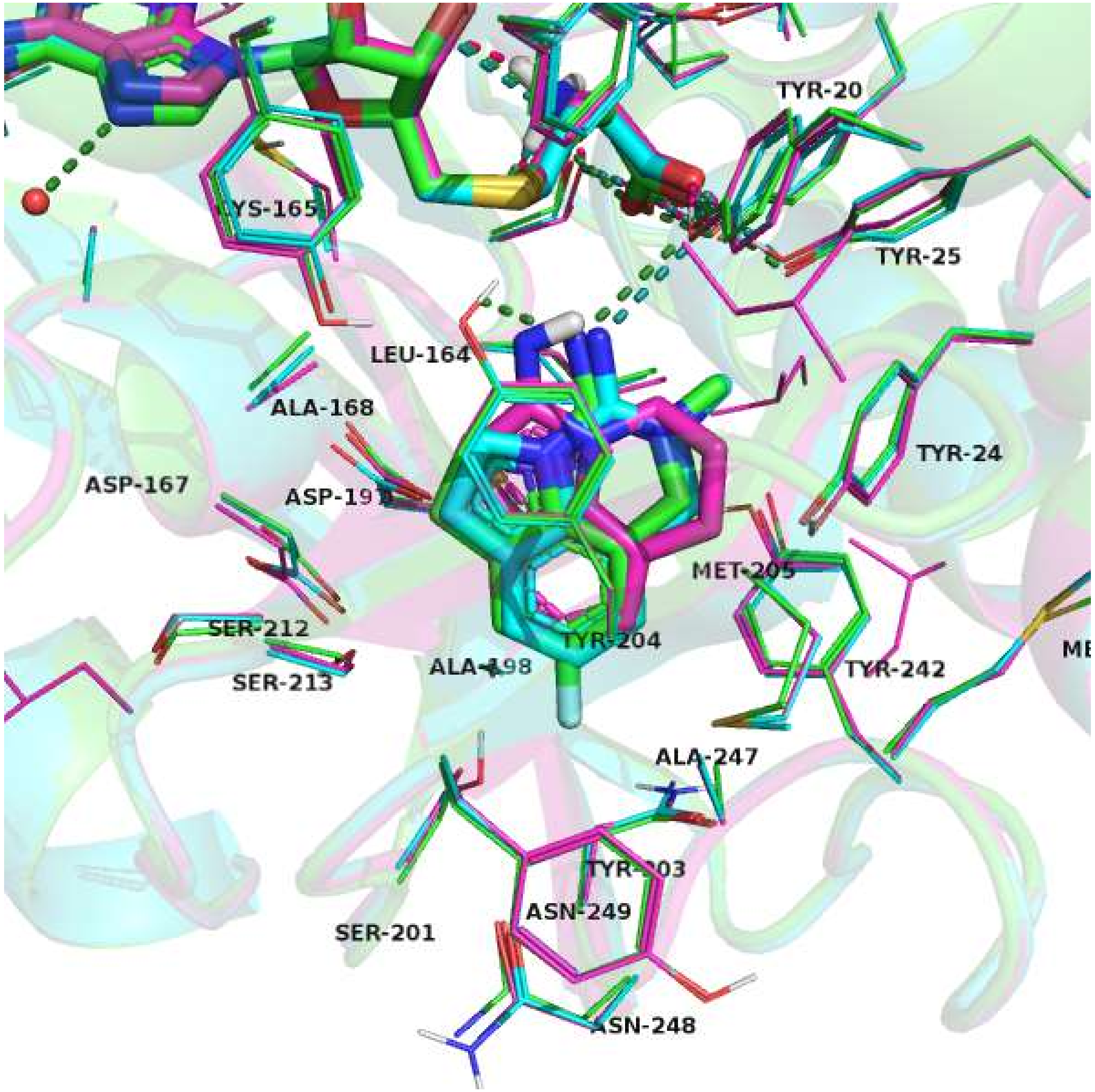

Figure S2C. Aligned compounds JBSNF-107 (in green-color), JBSNF-28 (in cyan-color) and Compound-A (in magenta-color). Compound JBSNF107 shows H-bond interactions with Leu-164 and Tyr-20, while JBSNF28 shows with Tyr-20. Compound-A does not show any H-bond interactions. Compound JBSNF107 also has a Fluoro at the lower part that can gather some hydrophobic interactions, while compound JBSNF28 has a methyl on the right-hand side that can gather some hydrophobic interactions. Residues within 5A of the ligands are shown as lines and labelled.

Supplementary figure S2D

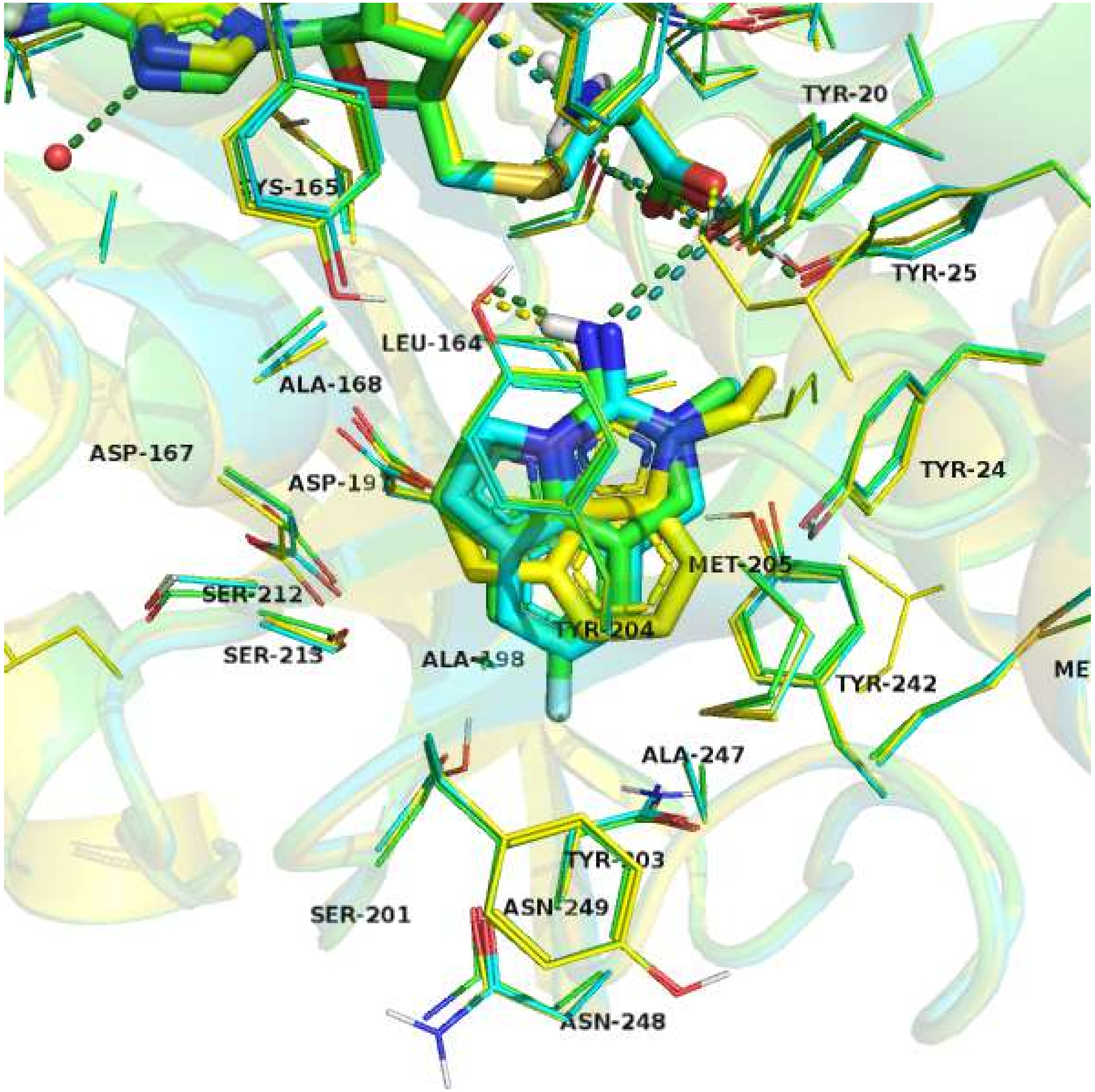

Figure S2D. Aligned compounds JBSNF-107 (in green-color), JBSNF-28 (in cyan-color) and Compound-B (in yellow-color). Compound JBSNF107 shows H-bond interactions with Leu-164 and Tyr-20, while JBSNF28 shows with Tyr-20. Compound-B shows H-bond interaction with Leu-164. Compound JBSNF107 also has a Fluoro at the lower part that can gather some hydrophobic interactions, while compound JBSNF28 has a methyl on the right-hand side that can gather some hydrophobic interactions. Compound-B has a ethyl substituent at the same place and can gather some hydrophobic interaction from surrounding residues. Residues within 5A of the ligands are shown as lines and labelled.

Supplementary figure S3A
1H-NMR of **(6)** (JBSNF-000028)

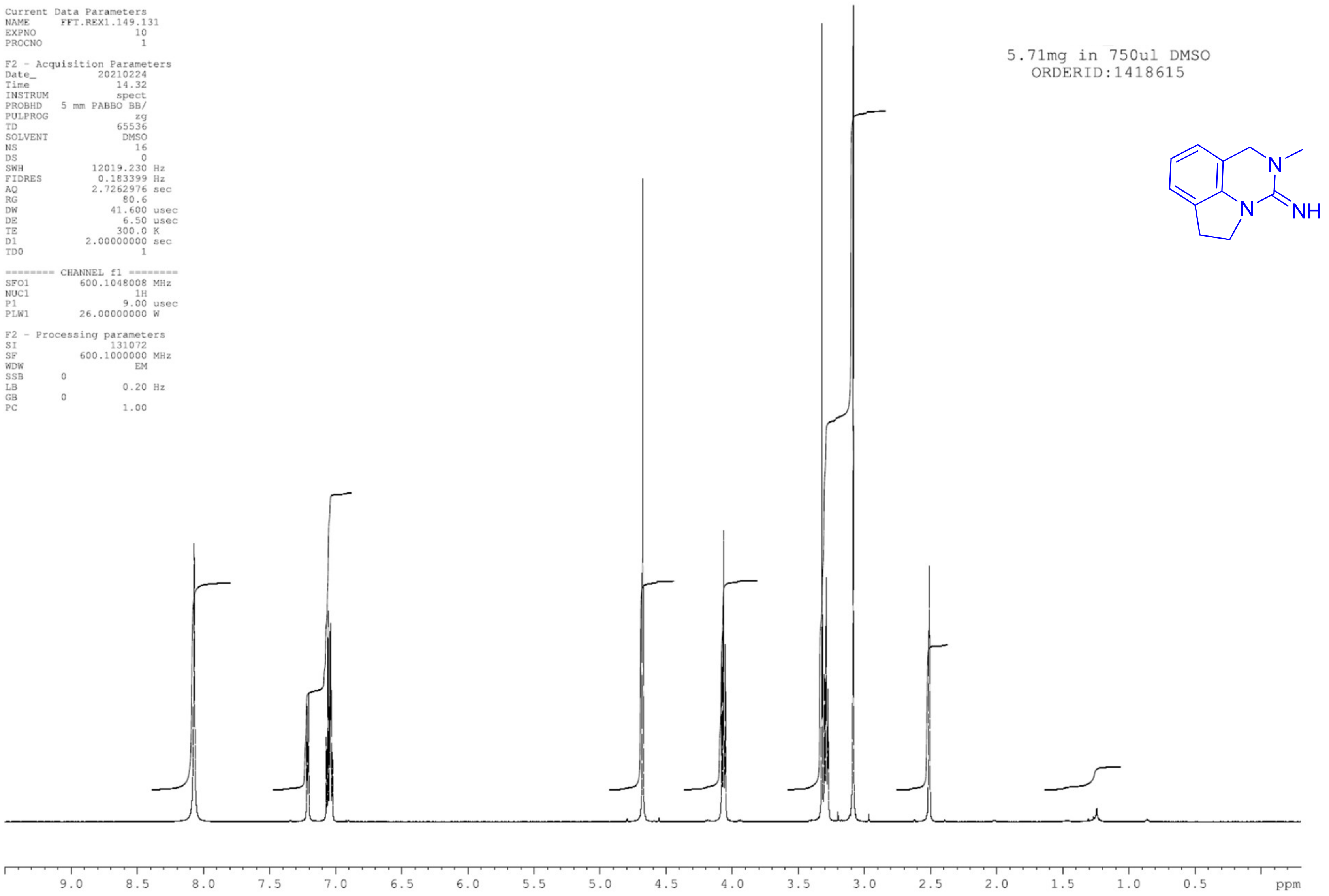

Supplementary figure S3B
13C-NMR of **(6)** (JBSNF-000028)

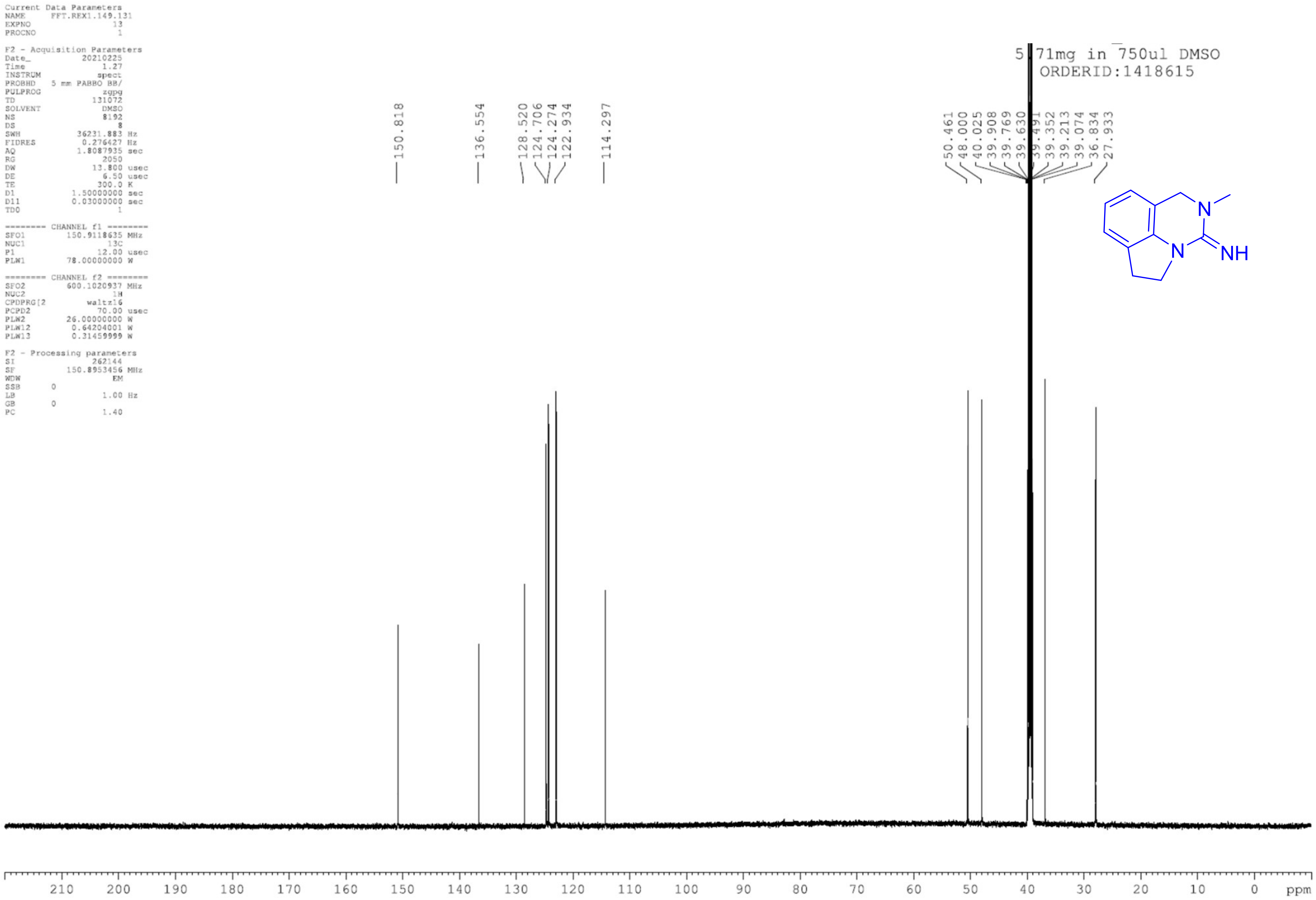

Supplementary figure S3C
COSY-NMR of **(6)** (JBSNF-000028)

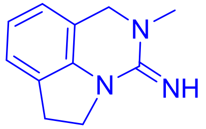

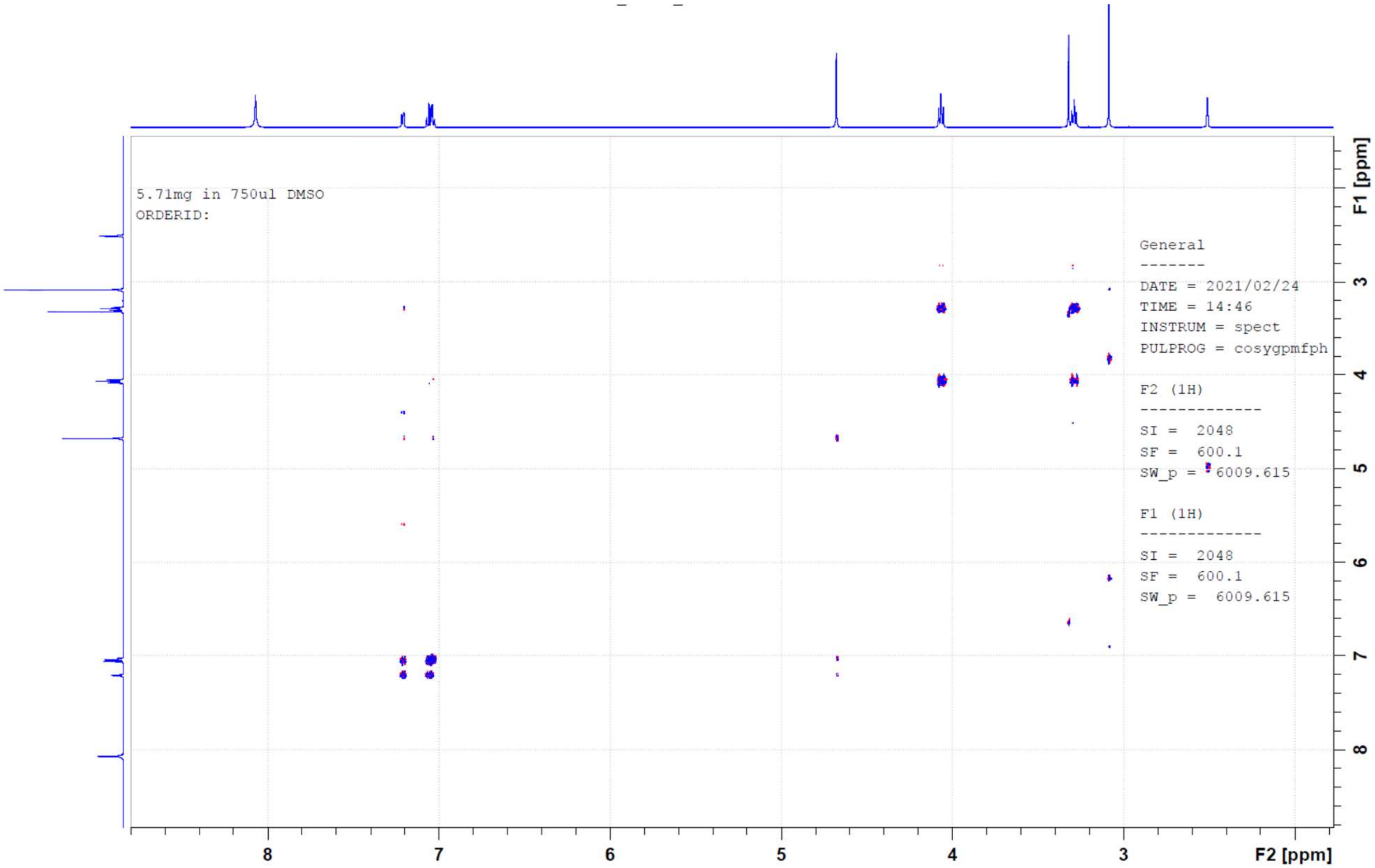

Supplementary figure S3D
HSQC-NMR of **(6)** (JBSNF-000028)

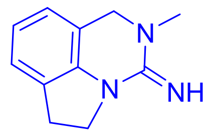

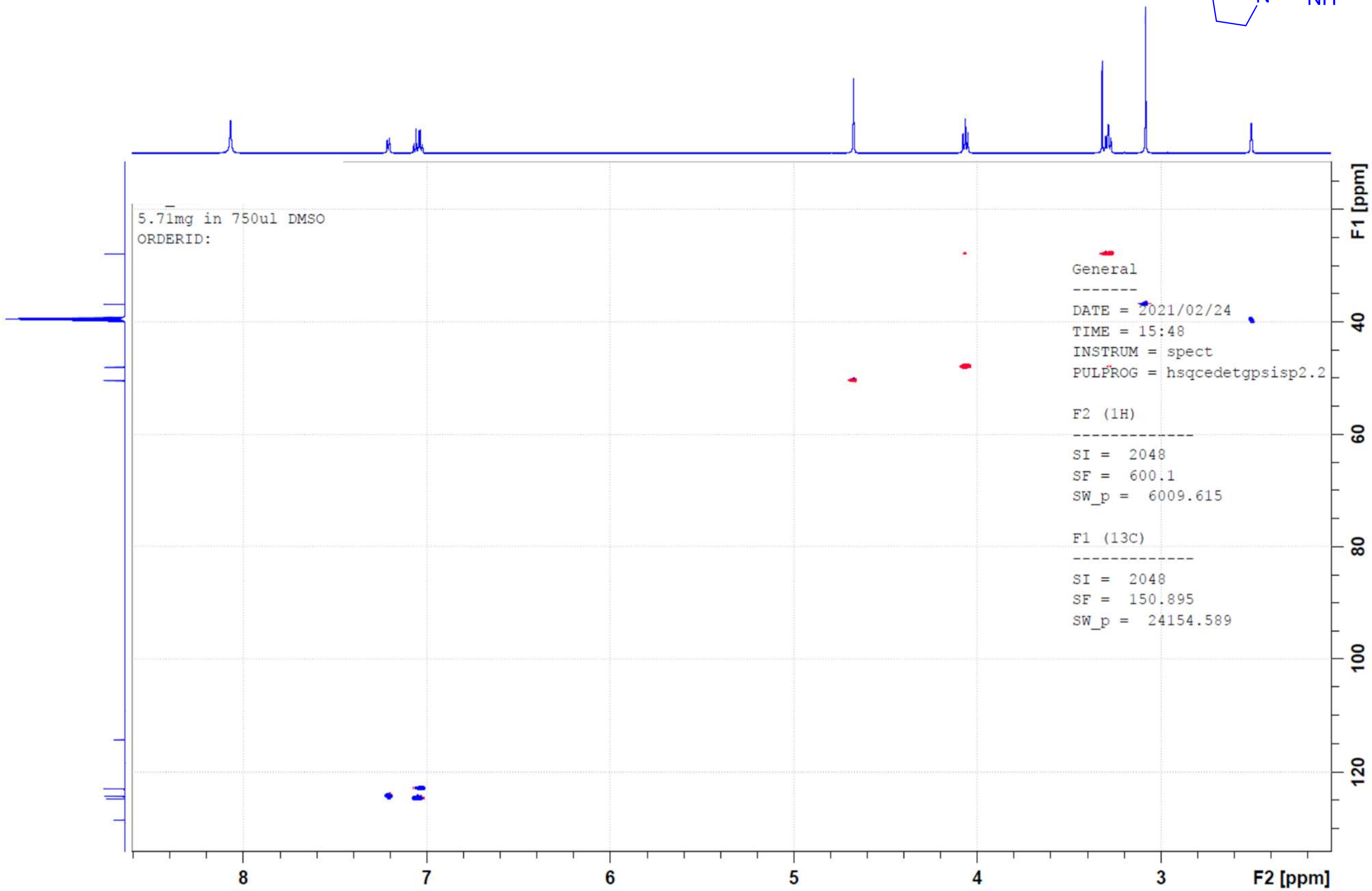

Supplementary figure S3E HRMS Report of **(6)** (JBSNF-000028)

**Data File**	210226_HR_21024-Q_TOF-HRMS-3722_005_d.d	**Sample Name**	FFT.REX1.149.131 (Ref:5)
**Sample Type**	Sample	**Position**	P1-E1
**Instrument Name**	qTOF 6550	**User Name**	Ana Villar Garea
**Acq Method**	100917_Matheus_grad_caffeine_fragm225_vcap3000_vnoz1500.m	**Acquired Time**	26.02.2021 15:46:43
**IRM Calibration Status**	Success	**DA Method**	151105_QC_based-on-V9_ruf.m
**Comment**	NMR sample diluted 1:100 in DMSO		

**Sample Group**		**Info.**	
**Formula**	C11H13N3	**Submitter**	JACOBY, NICOLA
**User**	Ana Villar Garea	**Stream Name**	LC 1
**Acquisition SW Version**	6200 series TOF/6500 series Q-TOF B.06.01 (B6157)		

Compound Table

Compound Label	RT	Mass	Abund	Formula	Tgt Mass	Diff (ppm)	MFG Formula	DB Formula
Cpd 2: C11 H13 N3	1.033	187.1117	175664	C11 H13 N3	187.1109	3.86	C11 H13 N3	C11 H13 N3
Cpd 1: C11 H13 N3	1.642	187.1116	118242	C11 H13 N3	187.1109	3.34	C11 H13 N3	C11 H13 N3

Compound Label	*m/z*	RT	Algorithm	Mass
Cpd 2: C11 H13 N3	188.1189	1.033	Find By Formula	187.1117

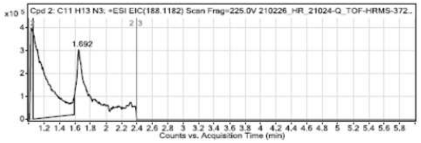

MS Spectrum

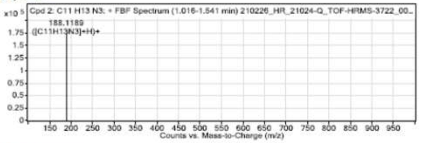

MS Zoomed Spectrum

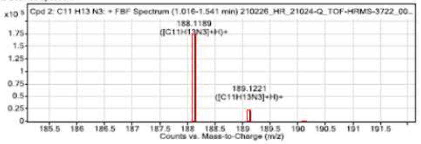

MS Spectrum Peak List

m/z	z	Abund	Formula	Ion
188.1189	1	175664.45	C11H13N3	(M+H)+
189.1221	1	21036.41	C11H13N3	(M+H)+
190.1252	1	1160.33	C11H13N3	(M+H)+

MS Spectrum

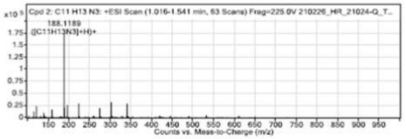

MS Zoomed Spectrum

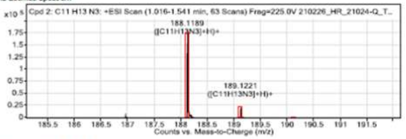

MS Spectrum Peak List

m/z	*Calc m/z*	Diff(ppm)	z	Abund	Formula	Ion
188.1189	188.1182	-3.64	1	175664.45	C11H13N3	(M+H)+
189.1221	189.1211	-5.29	1	21036.41	C11H13N3	(M+H)+
190.1252	190.1239	-7.01	1	1160.33	C11H13N3	(M+H)+

Compound Label	*m/z*	RT	Algorithm	Mass
Cpd 1: C11 H13 N3	188.1188	1.642	Find By Formula	187.1116

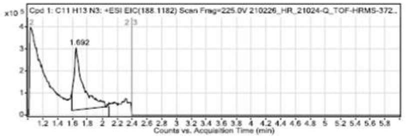

MS Spectrum

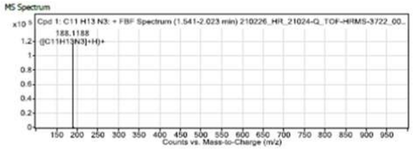

MS Zoomed Spectrum

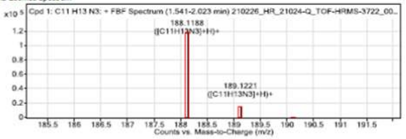

MS Spectrum Peak List

m/z	z	Abund	Formula	Ion
188.1188	1	118242.44	C11H13N3	(M+H)+
189.1221	1	14173.36	C11H13N3	(M+H)+
190.1254	1	785.78	C11H13N3	(M+H)+

MS Spectrum

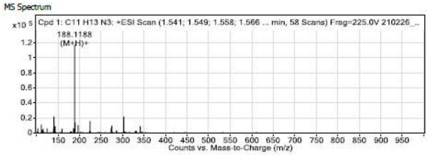

MS Zoomed Spectrum

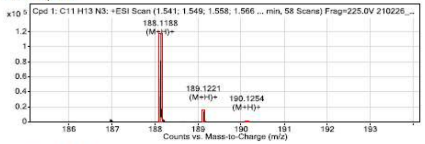

MS Spectrum Peak List

m/z	*Calc m/z*	Diff(ppm)	z	Abund	Ion
188.1188	188.1182	-3.08	1	118242.44	(M+H)+
188.1188				118242.44	
189.1221	189.1211	-5.12	1	14173.36	(M+H)+
190.1254	190.1239	-7.65	1	785.78	(M+H)+

--- End Of Report ---

Supplementary figure S3A
1H-NMR of **(8)** (JBSNF-000107)

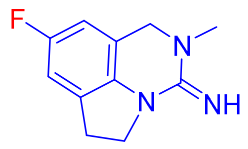

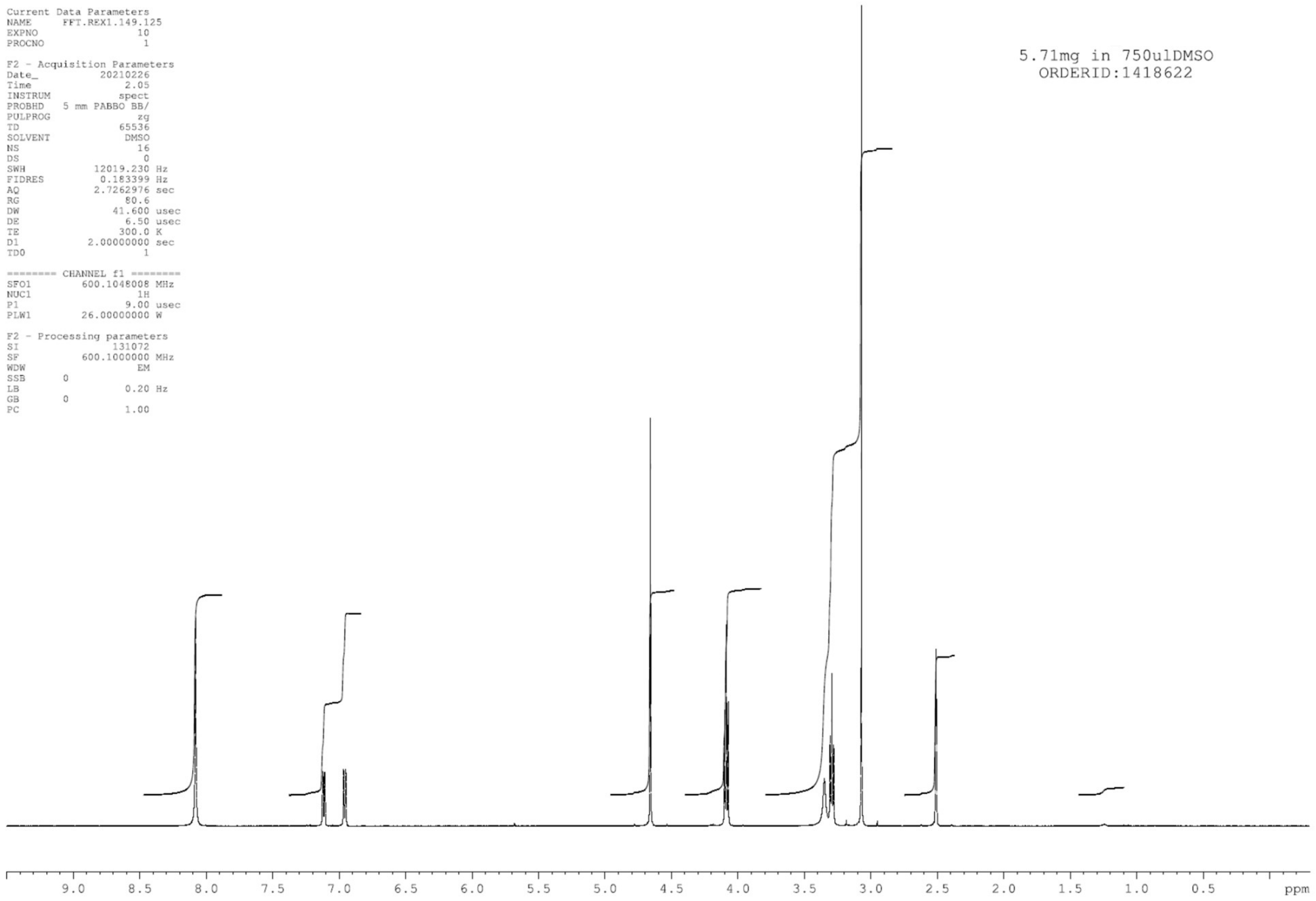

Supplementary figure S3A
13C-NMR of **(8)** (JBSNF-000107)

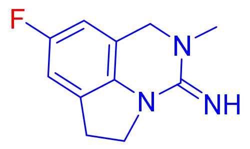

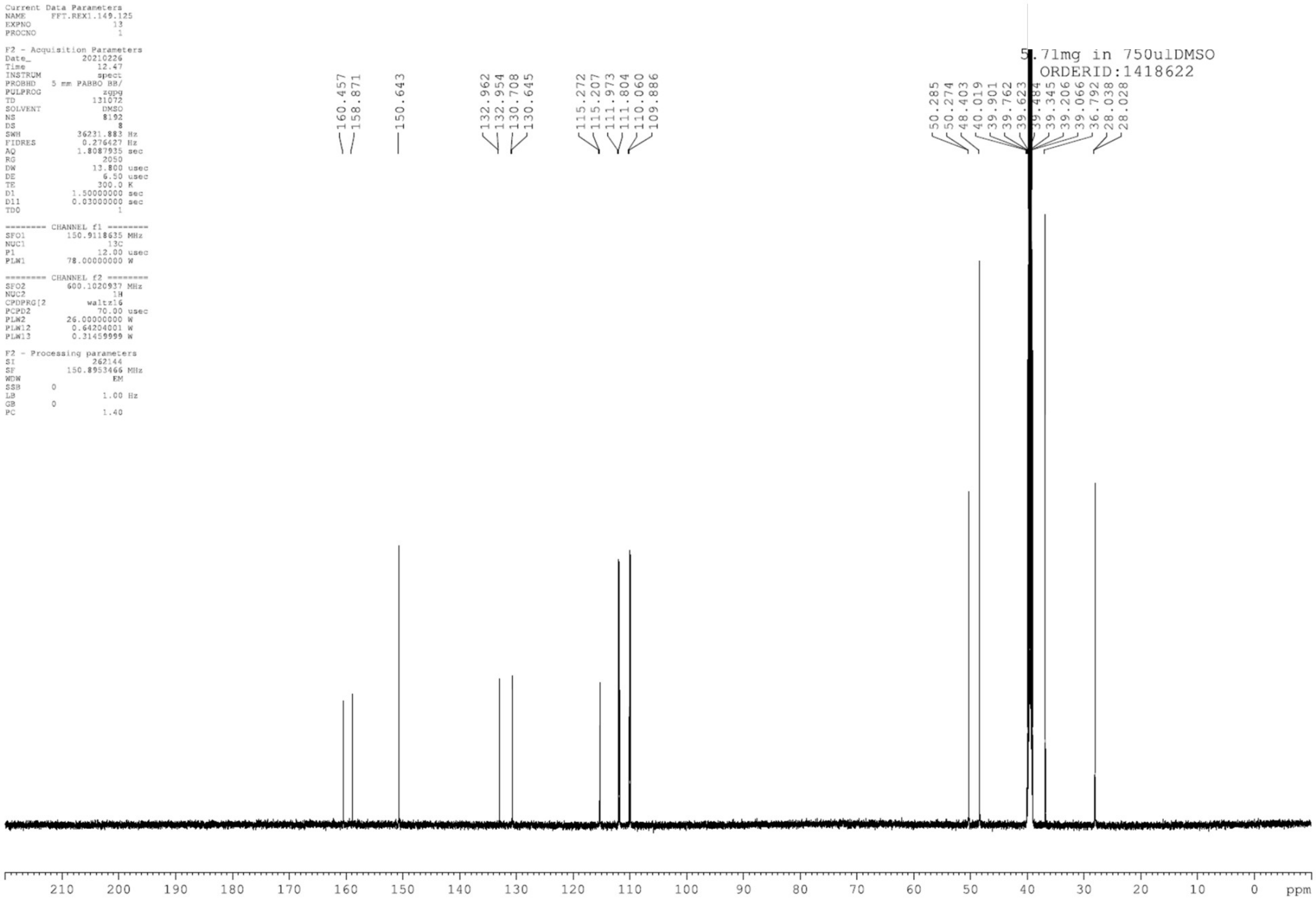

Supplementary figure S3A
COSY-NMR of **(8)** (JBSNF-000107)

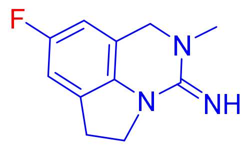

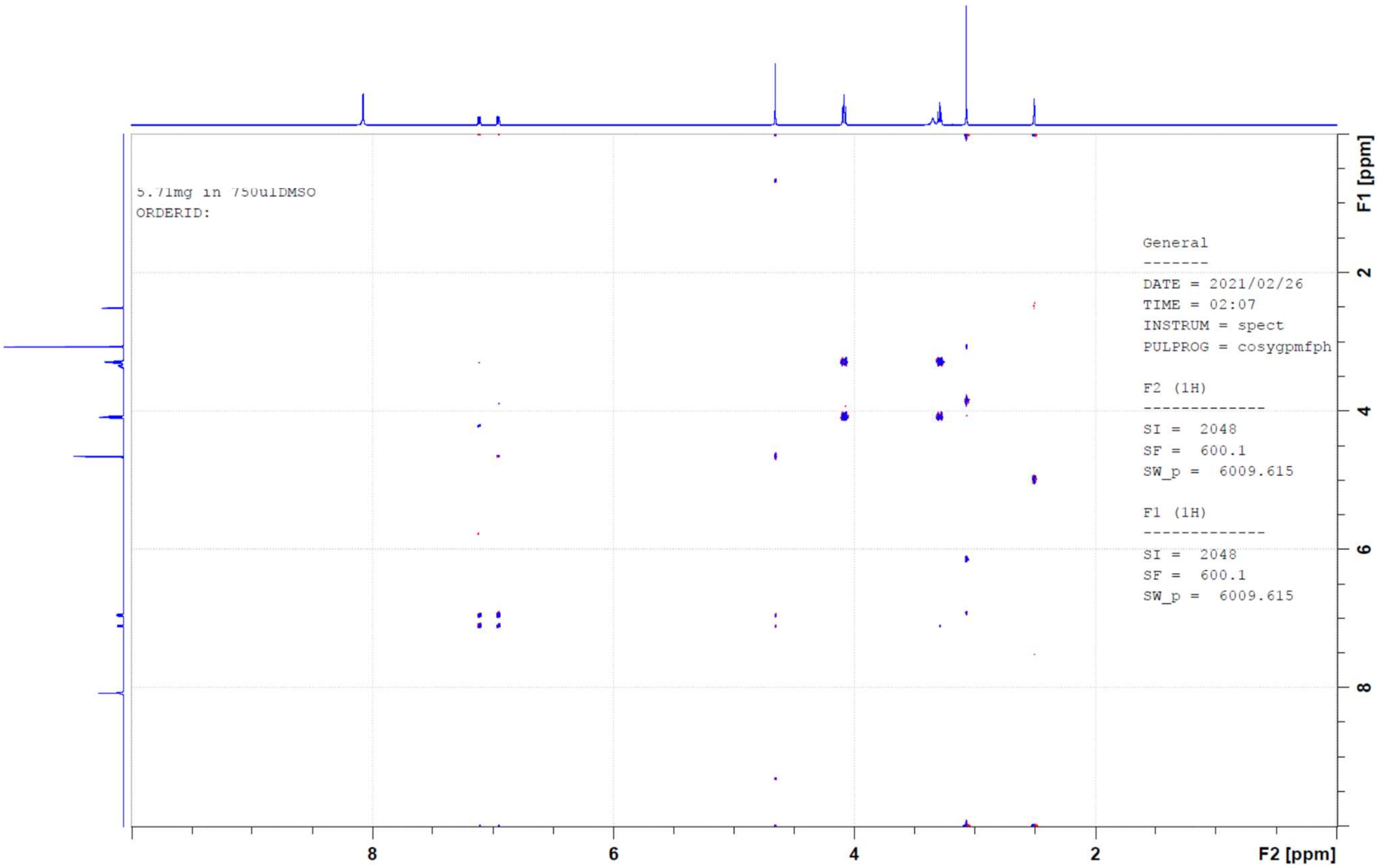

Supplementary figure S3A
HSQC-NMR of **(8)** (JBSNF-000107)

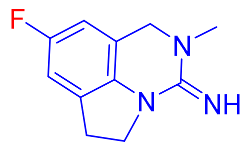

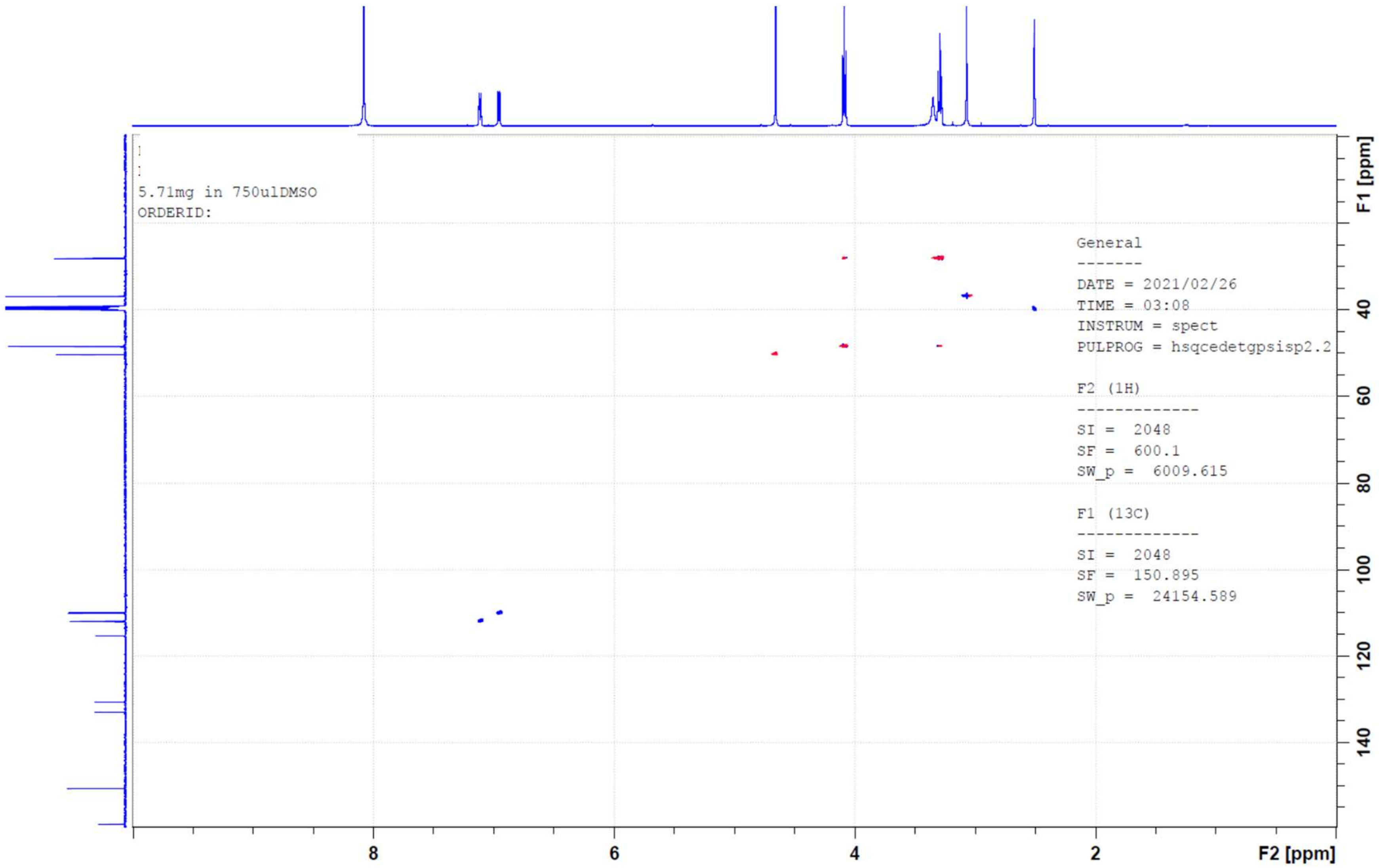

Supplementary figure S3E HRMS Report of **(8)** (JBSNF-000107)

**Data File**	210319FFT.REX1.149.125_2_1uL.d	**Sample Name**	
**Sample Type**	Sample	**Position**	
**Instrument Name**	TOF 6230	**User Name**	
**Acq Method**	200707_Quant0598_short column_1min_Vcap3000_nozzle1500_frag150_1Hz.m	**Acquired Time**	19.03.2021 22:12:41
**IRM Calibration Status**	Success	**DA Method**	151105_QC_based-on-V9_ruf.m
**Comment**			

**Sample Group**		**Info.**	
**Formula**	C11 H12 F N3	**Submitter**	
**User**		**Stream Name**	LC 1
**Acquisition SW Version**	6200 series TOF/6500 series Q-TOF B.08.00 (B8058.0)		

Compound Table

Compound Label	RT	Mass	Abund	Formula	Tgt Mass	Diff (ppm)	MFG Formula	DB Formula
Cpd 1: C11 H12 F N3	2.592	205.1012	294148	C11 H12 F N3	205.1015	-1.66	C11 H12 F N3	C11 H12 F N3

Compound Label	*m/z*	RT	Algorithm	Mass
Cpd 1: C11 H12 F N3	206.1084	2.592	Find By Formula	205.1012

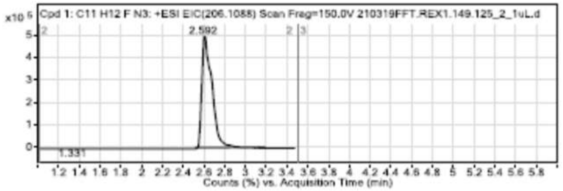

MS Spectrum

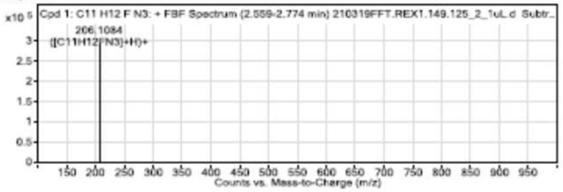

MS Zoomed Spectrum

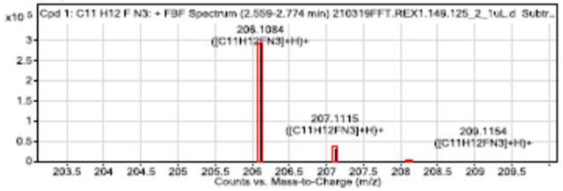

MS Spectrum Peak List

m/z	z	Abund	Formula	Ion
206.1084	1	294147.53	C11H12FN3	(M+H)+
207.1115	1	31748.82	C11H12FN3	(M+H)+
208.1149	1	1871.57	C11H12FN3	(M+H)+
209.1154	1	296.92	C11H12FN3	(M+H)+

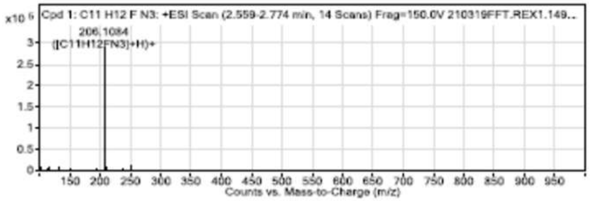

MS Zoomed Spectrum

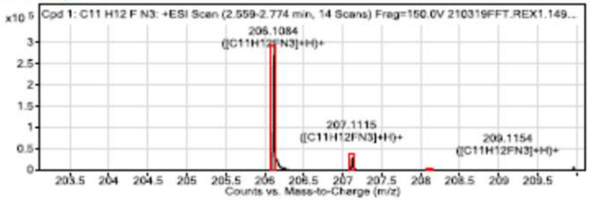

MS Spectrum Peak List

m/z	*Calc m/z*	Diff(ppm)	z	Abund	Formula	Ion
206.1084	206.1088	1.74	1	294147.53	C11H12FN3	(M+H)+
207.1115	207.1117	0.97	1	31748.82	C11H12FN3	(M+H)+
208.1149	208.1145	-1.9	1	1871.57	C11H12FN3	(M+H)+
209.1154	209.1172	8.54	1	296.92	C11H12FN3	(M+H)+
