# Supplementary material for: Novel tricyclic small molecule inhibitors of Nicotinamide N-methyltransferase for the treatment of metabolic disorders
Source: Sci Rep. 2022 Sep 14;12:15440. doi: 10.1038/s41598-022-19634-2 (PMC9474883; doi:10.1038/s41598-022-19634-2)
Supplement: Supplementary file 2 — Supplementary Information 2. [file 41598_2022_19634_MOESM2_ESM.docx]

**Supplementary Information for**

**Novel tricyclic small molecule inhibitors of Nicotinamide N*-*methyltransferase for the treatment of metabolic disorders**

Sven Ruf^1#^, Sridharan Rajagopal^2#^, Sanjay Venkatachalapathi Kadnur^3^, Mahanandeesha S Hallur^3^, Shilpa Rani^3^, Rajendra Kristam^3^, Srinivasan Swaminathan^3^, Bharat Ravindra Zope^3^, Pavan Kumar Gondrala^3^, Indu Swamy^3^, Rama Kishore Putta V P^3^, Saravanan Kandan^3^, Gernot Zech^1^, Herman Schreuder^1^, Christine Rudolph^1^, Ralf Elvert^1,4^, Joerg Czech^1^, Swarnakumari Birudukota^3^, Amir Siddiqui M^3^, Niranjan Naranapura Anand^3^, Vishal Subhash Mane^3^, Sreekanth Dittakavi^3^, Juluri Suresh^3^, Ramachandraiah Gosu^3^, Mullangi Ramesh^3^, Takeshi Yura^3^, Saravanakumar Dhakshinamoorthy^3*^, Aimo Kannt^1,5*^

*^1^Sanofi-Aventis Deutschland GmbH, R&D, Integrated Drug Discovery, Industriepark Hoechst, 65926 Frankfurt am Main, Germany*

*^2^ Jubilant Therapeutics India Ltd, Bangalore 560022*

*^3^Jubilant Biosys Ltd, Bangalore-560022, India*

*^4^Evotec GmbH, Marie-Curie-Straße 7, 37079 Göttingen, Germany*

*^5^Fraunhofer-Institute for Translational Medicine and Pharmacology ITMP, Theodor-Stern-Kai 7, 60596 Frankfurt am Main, Germany*

*^#^ joint first authors*

*^*^ corresponding authors*

Saravanakumar Dhakshinamoorthy ([Saravanakumar.Dhakshinamoorthy@jubilantbiosys.com](mailto:Saravanakumar.Dhakshinamoorthy@jubilantbiosys.com))

Aimo Kannt ([Aimo.Kannt@itmp.fraunhofer.de](mailto:Aimo.Kannt@itmp.fraunhofer.de))

**Supplementary Methods**

**Compound synthesis and characterization**

**Compound (1)**

**Synthesis of 9-fluoro-2-methyl-1, 2, 6, 7-tetrahydropyrido [3, 2, 1-ij] quinazolin-3(5H)-imine-hydrobromide (1)-** was synthesized using the procedure described for **Compound (2)** using Quinoline-8-carboxylic acid-^1^H NMR (DMSO-d_6_, 400 MHz) δ 1.99 – 1.94 (m, 2H), 2.72 (t, *J* = 6.4 Hz, 2H), 3.12 (s, 3 H), 3.71 (t, *J* = 5.6 Hz, 2H), 4.46 (s, 2H), 7.09 - 7.01 (m, 2H), 7.11 (d, *J* = 7.2 Hz, 1H), 8.02 (s, 2H); HPLC Purity @ 280 nm, 99.87%. MS (ESI) m/z 202.3 (M+H)+ -HBr.

**Compound (2)**

**Synthesis of 6-fluoro-N-methylquinoline-8-carboxamide (2)-**To a solution of 6-fluoroquinoline-8-carboxylic acid (**1**, 5.0 g, 26.17 mmol) in dichloromethane (60 mL) was added triethylamine (7.92 mL, 78.51mmol) at 0 °C followed by 2M methylamine solution in THF (15.70 mL, 31.41 mmol) and 1-propane phosphoric acid cyclic anhydride (24.96 mL, 78.51 mmol), and stirred for 12 h at room temperature, reaction mixture was concentrated under reduced pressure, and added water (10 mL), then extracted in to EtOAc, Organic layer was washed with saturated NaHCO_3_ solution and brine, dried over sodium sulphate, filtered and concentrated under reduced pressure. The obtained crude was purified by combi-flash purifier with 20% ethyl acetate in hexane as eluent to afford the desired product **(2)** as a sandal color solid (4.4 g, 83 %). ^1^H NMR (DMSO-d_6_, 400 MHz) δ 2.97 (d, *J* = 4.0 Hz, 3H), 7.70 (dd, *J* = 4.0 Hz, *J* = 8.0 Hz, 1H), 8.01 (dd, *J* = 2.4 Hz, *J* = 6.0 Hz, 1H), 8.28 (dd, *J* = 7.2 Hz, *J* = 7.6 Hz, 1H), 8.52 (d, *J* = 8.4 Hz, 1H), 9.00-9.01 (m, 1H), 10.56 (s, 1H); MS (ESI) *m/z* 205.4 (M+H)^+^.

**Synthesis of 6-fluoro-N-methyl-1, 2, 3, 4-tetrahydroquinoline-8-carboxamide (3)**-To a solution of 6-fluoro-N-methylquinoline-8-carboxamide (**2**, 1.0 g, 4.90 mmol) in methanol (10.0 mL) was added PtO_2_ (0.3 g), then the mixture was stirred for 1.5 h under H_2_ atmosphere. After completion of the reaction [monitored by TLC, ethyl acetate: hexane (3:7)]. The reaction mixture was filtered through diatomaceous earth; Organic layer concentrated under reduced pressure to afford the desired product **(3)** as a yellow solid (0.8 g, 80 %); MS (ESI) *m/z* 209.1 (M+H)^+^.

**Synthesis of 1-(6-fluoro-1, 2, 3, 4-tetrahydroquinolin-8-yl)-N-methylmethanamine (4)-** To a solution of 6-fluoro-N-methyl-1,2,3,4-tetrahydroquinoline-8-carboxamide (**3**, 0.8 g, 3.84 mmol) in tetrahydrofuran (10.0 mL) was added 2M LiAlH_4_ Solution in THF (2.97 mL, 5.76 mmol) at 0 °C, and the reaction mixture was stirred at 65 °C for 48h. After refluxing 48 h the reaction mixture was quenched with brine solution (1.0 mL) at 0 °C and diluted with ethyl acetate (50.0 mL), then it was filtered through diatomaceous earth and concentrated under reduced pressure to get crude compound. The obtained crude was purified by combi-flash purifier with 7% methanol in dichloromethane as eluent to afford the desired product **(4)** as a viscous oil (0.25 g, 33 %).

^1^H NMR (DMSO-*d*_6,_ 400 MHz) δ 1.75 (t, *J* = 6.0 Hz, 2H), 2.10 (bs, 1H), 2.23 (s, 3H), 2.60 (t, *J* = 5.6 Hz, 2H), 3.18 (t, *J* = 5.2 Hz, 2H), 3.46 (s, 2H), 5.59 (bs, 1H), 6.60 (dd, *J* = 2.4 Hz, *J* = 5.2 Hz, 1H), 6.67 (dd, *J* = 2.4 Hz, *J* = 6.4 Hz, 1H); MS (ESI) *m/z* 195.1 (M+H)^+^.

**Synthesis of 9-fluoro-2-methyl-1, 2, 6, 7-tetrahydropyrido [3, 2, 1-ij] quinazolin-3(5H)-imine-Hydrobromide (Compound 2)-** To a solution of 1-(6-fluoro-1, 2, 3, 4-tetrahydroquinolin-8-yl)-N-methylmethanamine (**4**, 0.25 g, 1.28 mmol) in ethanol (5.0 mL), then cyanogen bromide (0.21 g, 1.93 mmol) was added, and the reaction mixture was stirred at 85 °C for 3 h, after cooling to room temperature solid was formed which was filtered through Buchner funnel and then solid was washed with ethanol (2.0 mL), pentane and diethyl ether. Finally collect the entire solid to afford the title **(Compound 2)** (0.25 g, 88%) as a light orange color solid. ^1^H NMR (DMSO-*d*_6,_ 400 MHz) δ 1.95 (t, *J* = 5.2 Hz, 2H), 2.74 (t, *J* = 6.0 Hz, 2H), 3.11 (s, 3H), 3.70 (t, *J* = 5.6 Hz, 2H), 4.46 (s, 2H), 6.95 (d, *J* = 5.6 Hz, 1H), 7.03 (d, *J* = 9.2 Hz, 1H), 8.04 (bs, 2H); ^13^C NMR (DMSO-*d*_6_) δ 158.02, 152.69, 128.48, 125.88, 123.67, 114.73, 110.49, 49.01, 48.77, 39.15, 25.55, 19.94.MS (ESI) *m/z* 220.1 (M+H)^+^-HBr;HPLC Purity @ 254nm,99.76%.

# Compound (3)

**Synthesis of 3-chloro-N-(3, 4-difluorophenyl) propanamide (2)**-To a solution of 3, 4-difluoroaniline (**1**, 5.0 g, 52.0 mmol) in acetone (50.0 mL) was added pyridine (10.50 mL, 130.1 mmol) and 3-chloro-propionyl chloride (6.0 mL, 62.2 mmol) at 0 °C and stirred for 4h at 55 °C. After completion of the reaction [monitored by TLC, ethyl acetate: hexane (1:9)]. The reaction mixture was quenched with cold water (15.0 mL) and extracted into ethyl acetate. The organic layer was washed with water and brine, dried over sodium sulphate, filtered and concentrated under reduced pressure to afford the desired product **(2)** as a light brown solid (7.5 g, 88 %).^1^H NMR (DMSO-*d*_6,_ 400 MHz) δ 2.79 (t, *J* = 6.0 Hz, 2H), 3.85 (t, *J* = 6.0 Hz, 2H), 7.26 (d, *J* = 8.8 Hz, 1H), 7.35 (q, *J* = 10.0 Hz, 1H), 7.73-7.79 (m, 1H), 10.26 (s, 1H); MS (ESI) *m/z* 218.0 (M-H)^+^.

**Synthesis of 6, 7-difluoro-3, 4-dihydroquinolin-2(1H)-one (3)-** To a mixture of 3-chloro-N-(3, 4-difluorophenyl) propanamide (**2**, 6.0 g, 29.7 mmol) in AlCl_3_ (13.8 g, 104.1 mmol) at 0 °C and stirred for 12 h at 120 °C. After completion of the reaction [monitored by TLC, ethyl acetate: hexane (2:8)]. The reaction mixture was quenched with 50% aqueous HCl solution (20.0 mL) and extracted into ethyl acetate. The organic layer was washed with water and brine, dried over sodium sulphate, filtered and concentrated under reduced pressure to afford the desired product **(3)** as a grey solid (4.8 g, 95 %). ^1^H NMR (DMSO-*d*_6,_ 400 MHz) δ 2.41 (t, *J* = 8.0 Hz, 2H), 2.83 (t, *J* = 8.0 Hz, 2H), 6.78 (q, *J* = 4.0 Hz, 1H), 7.26 (t, *J* = 10.0 Hz, 1H), 10.03 (s, 1H); MS (ESI) *m/z* 184.1 (M+H)^+^.

**Synthesis of 6, 7-difluoro-1, 2, 3, 4-tetrahydroquinoline (4)-** To a solution of 6, 7-difluoro-3, 4-dihydroquinolin-2(1H)-one (**3**, 6.0, 32.78 mmol) in tetrahydrofuran (50.0 mL), was added BH_3_.DMS (4.92 mL, 65.57 mmol) at 0 °C and stirred for 12 h at 65 °C. The reaction mixture was quenched with methanol at 0 °C and concentrated under reduced pressure to afford the desired product and then .HCl in dioxane (15.0 mL) was added to the mixture then stirred for 2 h at room temperature, again concentrated under reduced pressure. The reaction mixture was basified with saturated NaHCO_3_ solution and extracted into ethyl acetate. The organic layer was washed with water and brine, dried over sodium sulphate, filtered and concentrated under reduced pressure. The obtained crude was purified by combi-flash purifier with 5% Ethyl acetate in hexane as eluent to afford the desired product **(4)** as a viscous oil (4.7 g, 84 %). ^1^H NMR (DMSO-d_6,_ 400 MHz) δ 1.91 (t, *J* = 6.0 Hz, 2H), 2.67 (t, *J* = 6.0 Hz, 2H), 3.24 (t, *J* = 4.8 Hz, 2H), 3.72 (bs, 1H), 6.22 (q, *J* = 4.8 Hz, 1H), 6.72 (d, *J* = 9.6 Hz, 1H).

**Synthesis of (E)-2-(6, 7-difluoro-3, 4-dihydroquinolin-1(2H)-yl)-2-oxoacetaldehydeoxime (5)-** To a solution of 2,2,2-trichloroethane-1,1-diol (1.93 g, 11.70 mmol) in water (25.0 mL) was treated with sodium sulphate (13.84 g, 97.5 mmol). To the reaction mixture 6,7-difluoro-1,2,3,4-tetrahydroquinoline hydrochloride (**4**, 2.0 g, 9.75 mmol) in water was added followed by hydroxylamine hydrochloride (2.01 g, 29.25 mmol) in water was added and stirred for 5 h at 80 °C. After completion of the reaction [monitored by TLC, ethyl acetate: hexane (3:7)]. The reaction mixture was cooled to room temperature and basified with sat., NaHCO_3_ solution and extracted into ethyl acetate. The organic layer was washed with water and brine, dried over sodium sulphate, filtered and concentrated under reduced pressure. The obtained crude was washed with DCM/pentane mixture and followed by Ether/Pentane to afford the desired product **(5)** as a grey solid (1.5 g, 65 %). ^1^H NMR (DMSO-d_6,_ 400 MHz) δ 1.88 (t, *J* = 6.4 Hz, 2H), 2.69 (t, *J* = 6.4 Hz, 2H), 3.72(t, *J* = 6.4 Hz, 2H), 7.28 (t, *J* = 9.2 Hz, 1H), 7.48 (bs, 1H), 7.79 (s, 1H), 11.91 (s, 1H).

**Synthesis of 8, 9-difluoro-5, 6-dihydro-4H-pyrrolo [3, 2, 1-ij] quinoline-1, 2-dione (6)-** To a stirred solution of H_2_SO_4_ (**5**, 0.45 g, 1.98 mmol) in water (6.0 mL) was heated to 55 °C for 10 mins, After reaching into 55 °C (E)-2-(6, 7-difluoro-3, 4-dihydroquinolin-1(2H)-yl)-2-oxoacetaldehyde oxime (1.5 g, 6.25 mmol) was added portionwise over the period of 15 mins, then the reaction mixture was heated to 80 °C until the completion of the reaction [ monitored by TLC, ethyl acetate: hexane (5:5)]. The reaction mixture was diluted with water and extracted into ethyl acetate. Organic layer was washed with water and brine, dried over sodium sulphate, filtered and concentrated under reduced pressure. The obtained crude was washed with DCM/pentane mixture and followed by Ether/Pentane to afford the desired product **(6)** as a light reddish powder (1.0 g, 76 %). ^1^H NMR (DMSO-d_6,_ 400 MHz) δ 1.88 (t, *J* = 6.0 Hz, 2H), 2.66 (d, *J* = 6.0 Hz, 2H), 3.56 (t, *J* = 6.0 Hz, 2H), 7.56 (q, *J* = 8.0 Hz, 1H), 7.70 (bs, 1H), 12.98 (bs, 1H); ^13^C NMR (DMSO-*d*_6_) δ 152.34, 146.50, 144.47, 142.11, 128.02, 122.74, 116.27, 110.02, 44.75, 37.95, 24.88, 20.45. MS (ESI) *m/z* 224.1 (M+H)^+^.

**Synthesis of 6, 7-difluoro-1, 2, 3, 4-tetrahydroquinoline-8-carboxylic acid (7)-** To a solution of 8, 9-difluoro-5, 6-dihydro-4H-pyrrolo [3, 2, 1-ij] quinoline-1, 2-dione (**6**, 1.0 g, 4.48 mmol) in H_2_O_2_ (2.0 ml) was added 30% aqueous NaOH solution (10.0 mL) then the mixture was stirred at room temperature for 1 h. After completion of the reaction [monitored by TLC, ethyl acetate: hexane (5:5)]. The reaction mixture was acidified with saturated Citric acid solution and extracted into ethyl acetate. Organic layer was washed with water and brine, dried over sodium sulphate, filtered and concentrated under reduced pressure to get desired product (7) as a pale yellow solid (0.65 g, 68 %). ^1^H NMR (DMSO-d_6,_ 400 MHz) δ 1.73 (t, *J* = 5.2 Hz, 2H), 2.46-2.48 (m, 2H), 2.64 (s, 2H), 7.10 (t, *J* = 6.0 Hz, 1H), 10.11 (bs, 1H); MS (ESI) *m/z* 214.1 (M+H)^+^.

**Synthesis of 6, 7-difluoro-N-methyl-1, 2, 3, 4-tetrahydroquinoline-8-carboxamide (8)-** To a solution of 6, 7-difluoro-1, 2, 3, 4-tetrahydroquinoline-8-carboxylic acid (**7**, 0.65 g, 3.05 mmol) in dichloromethane (20.0 mL) was added triethylamine (0.932 mL, 9.15mmol) at 0 °C followed by 2M methylamine solution in THF (2.30 mL, 4.57 mmol) and 1-propane phosphoric acid cyclic anhydride (2.9 mL, 9.15 mmol), and stirred for 12 h at room temperature, reaction mixture was concentrated under reduced pressure, and added water (10 mL), then extracted into EtOAc, Organic layer was washed with saturated NaHCO_3_ solution and brine, dried over sodium sulphate, filtered and concentrated under reduced pressure to afford the desired product (8) as a brown solid (0.6 g, 86 %).^1^H NMR (DMSO-d_6,_ 400 MHz) δ 1.71 (t, *J* = 5.2 Hz, 2H), 2.61 (t, *J* = 5.6 Hz, 2H), 2.73 (d, *J* = 10.0 Hz, 3H), 3.17 (s, 2H), 6.09 (s, 1H), 6.97 (t, *J* = 10.0 Hz, 1H), 8.24 (bs, 1H); MS (ESI) *m/z* 227.1 (M+H)^+^.

# Synthesis of 1-(6, 7-difluoro-1, 2, 3, 4-tetrahydroquinolin-8-yl)-N-methylmethanamine (9)-To a solution of 6,7-difluoro-N-methyl-1,2,3,4-tetrahydroquinoline-8-carboxamide (8, 0.6, 2.65 mmol) in tetrahydrofuran (10.0 mL), was added BH_3_.DMS (4.00 mL) at 0 °C and stirred for 48 h at 65 °C, then the reaction mixture was quenched with methanol at 0 °C and concentrated under reduced pressure to afford the desired product, and then 15.0 mL of HCl in dioxane was added to the mixture then stirred for 2 h at room temperature again concentrated under reduced pressure. The reaction mixture was basified with saturated NaHCO_3_ solution and extracted into ethyl acetate. Organic layer was washed with water and brine, dried over sodium sulphate, filtered and concentrated under reduced pressure. The obtained crude was purified by combi-flash purifier with 7% Methanol in Dichloromethane as eluent to afford the desired product (9) as a yellow oil (0.31 g, 55 %).^1^H NMR (DMSO-d_6,_ 400 MHz) δ 1.70-1.76 (m, 2H), 2.22 (s, 3H), 2.61 (t, *J* = 5.6 Hz, 2H), 3.18 (t, *J* = 5.2 Hz, 2H), 3.58 (s, 2H), 5.91 (bs, 1H), 6.80 (t, *J* = 9.6 Hz, 1H); MS (ESI) *m/z* 213.1 (M+H)^+^.

# Synthesis of 9, 10-difluoro-2-methyl-1, 2, 6, 7-tetrahydro-3H, 5H-pyrido [3, 2, 1-ij] quinazolin-3-iminehydrobromide (Compound 3)- To a solution of 1-(6,7-difluoro-1,2,3,4-tetrahydroquinolin-8-yl)-N-methylmethanamine (9, 0.31 g, 1.46 mmol) in ethanol (10.0 mL), then cyanogen bromide (0.18 g, 1.75 mmol) was added, and the reaction mixture was stirred at 85 °C for 2 h, after cooled to room temperature reaction mixture was concentrated under reduced pressure. The obtained solid was washed with ethanol, pentane, and ether, and then the solid was concentrated to afford the desired product (Compound 3) as a light pink solid (0.35 g, 75 %). ^1^H NMR (DMSO-d_6,_ 400 MHz) δ 1.94 (t, *J* = 5.6 Hz, 2H), 2.68 (t, *J* = 5.6 Hz, 2H), 3.14 (s, 3H), 3.68 (t, *J* = 6.0 Hz, 2H), 4.57 (s, 2H), 7.28 (t, *J* = 6.0 Hz, 2H), 8.12 (bs, 2H); MS (ESI) *m/z* 238.0 (M+H)^+^-HBr; HPLC Purity @ 284nm, 98.24%.

**Compound (4)**

# Synthesis of 8-bromo-1,2,3,4-tetrahydro-1,6-naphthyridine (2)- To a solution of 1,2,3,4-tetrahydro-1,6-naphthyridine (1, 0.5g, 3.73 mmol) in acetic acid (5.0 mL) was added N-Bromosuccinimide (0.73 g, 4.1 mmol) at 0 °C and stirred for 16 h at 80 °C. After completion of the reaction, the reaction mixture was concentrated under reduced pressure. The obtained crude was purified by combi-flash purifier with 3-20% ethyl acetate in hexane as eluent to afford the desired product (2) as an off-white solid (0.3 g, 38 % yield). MS (ESI) *m/z* 215.1 (M+2H)+.

# Synthesis of 1,2,3,4-tetrahydro-1,6-naphthyridine-8-carbonitrile (3)- To a solution of 8-bromo-1,2,3,4-tetrahydro-1,6-naphthyridine (2, 1 g, 4.69 mmol) in DMF (20 mL) was purged with nitrogen gas for 10 mins followed by added Pd_2_(dba)_3_ (0.429 g, 0.469 mmol) and dppf (0.695 g, 0.938 mmol) under inert atmosphere and the reaction mixture was purged again for 15 mins with nitrogen. Then zinc cyanide (0.82 g, 7.04 mmol) was added and the reaction mixture was stirred for 16 h at 100 °C. After stirring 16 h, the reaction mixture was quenched with water and extracted with ethyl acetate and separated the organic layer. Organic layer was washed with water and brine, dried over sodium sulphate, filtered and concentrated under reduced pressure to afford the desired product (3) as a yellow solid (1 g, crude). The compound was taken for next step without purification.

**Synthesis of 1, 2, 3, 4-tetrahydro-1, 6-naphthyridine-8-carboxylic acid (4)-**To a solution of 1, 2, 3, 4-tetrahydro-1, 6-naphthyridine-8-carbonitrile (**3**, 0.5 g, 3.14 mmol) in conc.HCl (15.0 mL) and the reaction mixture was stirred at 100 °C for 16 h. The reaction mixture was concentrated completely under reduced pressure and washed with pentane and diethyl ether to afford the desired product **(4)** as an off-white solid (0.35 g, crude). ^1^H NMR (DMSO-*d*_6,_ 400 MHz) δ 1.75-1.79 (m, 2H), 2.64-2.68 (m, 2H), 3.38-3.42 (m, 2H), 7.79 (s, 1H), 8.40 (s, 1H), 10.40 (brs, 1H), MS (ESI) *m/z* 179.1 (M+H)^+^.

**Synthesis of N-methyl-1, 2, 3, 4-tetrahydro-1, 6-naphthyridine-8-carboxamide (5)-** To a solution of 1, 2, 3, 4-tetrahydro-1, 6-naphthyridine-8-carboxylic acid (**4**, 0.35 g, 1.96 mmol) in dichloromethane (15.0 mL) was added triethylamine (0.9 mL, 9.8 mmol) at 0 °C followed by 2M methylamine solution in THF (2.0 mL, 3.93 mmol) and 1-propane phosphoric acid cyclic anhydride (1.86 mL, 5.88 mmol), and stirred for 12 h at room temperature, reaction mixture was concentrated under reduced pressure, and added water (10 mL), then extracted with EtOAc, Organic layer was washed with saturated NaHCO_3_ solution and brine, dried over sodium sulphate, filtered and concentrated under reduced pressure. The obtained crude was purified by combi-flash purifier with 7% methanol in dichloromethane as eluent to afford the desired product **(5)** as a viscous liquid (0.17 g, 48 % yield). ^1^H NMR (DMSO-*d*_6,_ 400 MHz) δ 1.75 (t, *J* = 5.2 Hz, 2H), 2.63 (t, *J* = 6.0 Hz, 2H), 2.71 (d, *J* = 4.4 Hz, 3H), 3.27-3.32 (m, 2H), 7.83 (s,1H), 8.30 (s, 1H), 8.33 (bs, 2H); MS (ESI) *m/z* 192.1 (M+H)^+^.

**Synthesis of N-methyl-1-(1, 2, 3, 4-tetrahydro-1, 6-naphthyridin-8-yl) methanamine (6)-**To a solution of N-methyl-1, 2, 3, 4-tetrahydro-1, 6-naphthyridine-8-carboxamide (**5**, 0.16 g, 0.837 mmol) in tetrahydrofuran (20.0 mL) was added 1M LiAlH_4_ solution in THF (4.18 mL, 4.18 mmol) at 0 °C and the reaction mixture was stirred at 65 °C for 48h. After refluxing overnight the reaction mixture was quenched with brine solution (1.0 mL) at 0 °C and diluted ethyl acetate, then it was filtered through diatomaceous earth and concentrated under reduced pressure to get desired crude compound **(6)** as a viscous oil (0.15 g, crude), MS (ESI) *m/z* 178.1 (M+H)^+^.

**Synthesis of 2-methyl-1,2,6,7-tetrahydro-3H,5H-pyrimido[5,6,1-ij][1,6]naphthyridin-3-imine Hydrobromide ( Compound 4)-**To a solution of N-methyl-1-(1, 2, 3, 4-tetrahydro-1, 6-naphthyridin-8-yl) methanamine (**6**, 0.15 g, 0.85 mmol) in ethanol (10.0 mL), then cyanogen bromide (0.108 g, 2.04 mmol) was added, and the reaction mixture was stirred at 80 °C for 2 h, after cooled to room temperature solid was formed which was filtered through Buckner funnel and then solid was washed with acetonitrile, pentane and diethyl ether. Finally collect the entire solid to afford the title **(Compound 4)** (0.06 g, 37%) as an off-white solid. ^1^H NMR (DMSO-*d*_6,_ 400 MHz) δ 2.74-2.78 (m, 2H), 3.15 (s, 3H), 3.71-3.75 (m, 2H), 4.58(s, 2H), 8.30-8.34 (m, 1H), 8.42-8.44 (m, 3H); MS (ESI) *m/z* 203.1 (M+H)^+^-HBr; HPLC Purity @254nm 98.52%.

**Compound (5)**

**Synthesis of 2,3,4,5-tetrahydro-1H-benzo[b]azepine hydrochloride (2)** To a solution of 2,3,4,5-tetrahydro-1H-benzo[b]azepine (2 g, 13.58 mmol) in dichloromethane (20 mL) was cooled to 0 °C, then 4 M HCl in dioxane (3 ml) was added and the reaction mixture was stirred at room temperature for 1h. The reaction mixture was concentrated completely under reduced pressure to give the crude compound (**2**) as a brown solid (2.45 g, 98.4 %). The crude proceeds to next step without further purification. ^1^H NMR (DMSO-*d_6_*_,_ 400 MHz) δ 1.65 (s, 2H), 2.05 (s, 2H), 2.95-2.97 (m, 2H), 3.22 (s, 2H), 7.29-7.32 (m, 3H), 7.55-7.57 (m, 1H), 11.18 (bs, 1H);

**Synthesis of (E)-2-oxo-2-(2,3,4,5-tetrahydro-1H-benzo[b]azepin-1-yl)acetaldehyde oxime (3)** To a solution of chloral hydrate ( 2.6 g, 15.6 mmol) in 25 ml of water was treated with sodium sulphate (18.6 g, 130 mmol). To this reaction mixture 2,3,4,5-tetrahydro-1H-benzo[b]azepine hydrochloride (**2**, 2.4 g, 13 mmol) in water (10 mL) was added followed by hydroxylamine hydrochloride (2.72 g, 39.1 mmol) in water (5 mL) was added and the reaction mixture was heated at 80 °C for 5 h. The reaction mixture was cooled to room temperature, basify using sodium bicarbonate solution and was extracted with ethyl acetate. The combined organic layer washed with brine solution and dried over anhydrous sodium sulphate, concentrated solvent under reduced pressure to give crude residue which was triturated using diethyl ether and n-pentane and dried completely to give the title product **3** as an off brownish solid (1.2 g, 42.1 % yield). MS (ESI) *m/z* 219.1 (M+H) ^+^.

**Synthesis of 1,2,3,4-tetrahydroazepino[3,2,1-hi]indole-6,7-dione (4)** To a solution of (E)-2-oxo-2-(2,3,4,5-tetrahydro-1H-benzo[b]azepin-1-yl)acetaldehyde oxime (**3,** 1.2 g, 5.49 mmol) in methanesulfonic acid (10 mL) was heated to 80 °C for 15 min. The reaction mixture was cooled to room temperature and poured into crushed ice, the solid precipitate was filtered through Buchner funnel, and the residue was washed with water and completely dried to give the title compound as a red solid (4**,** 0.98 g, 89 % yield). ^1^H NMR (CDCl_3,_ 400 MHz) δ 2.04 (s, 4H), 2.94 (d, *J* = 5.6 Hz, 2H), 3.99 (d, *J* = 5.2 Hz, 2H), 6.98 (t, *J* = 7.2 Hz, 2H), 7.29 (d, *J* = 7.2 Hz, 1H), 7.42 (d, *J* = 7.2 Hz, 1H); MS (ESI) *m/z* 202.1 (M+H) ^+^.

**Synthesis of 2,3,4,5-tetrahydro-1H-benzo[b]azepine-9-carboxylic acid (5)** To a solution of 1,2,3,4-tetrahydroazepino[3,2,1-hi]indole-6,7-dione (**4,** 0.6 g, 2.98 mmol) in 4.5 % sodium hydroxide solution (10 ml) was added 30 % hydrogen peroxide (2 ml) drop wise over 15 min. After the addition was complete, the reaction mixture was stirred at room temperature for 1 h. After completion of the reaction, the reaction mixture was acidified using citric acid solution (P^H^ ~ 3 to 4), the solid precipitate was filtered, the residue was washed with water and dried to give the title compound as pure compound as a white solid (**5,** 0.5 g, 87.7 % yield). ^1^H NMR (DMSO-*d_6_*_,_ 400 MHz) δ 1.65 (s, 2H), 1.83 (s, 2H), 2.86 (s, 2H), 3.24 (s, 2H), 6.90 (s, 1H), 7.34 (s, 1H), 7.74 (d, *J* = 7.6 Hz, 1H); MS (ESI) *m/z* 192.1 (M+H) ^+^.

**Synthesis of N-methyl-2,3,4,5-tetrahydro-1H-benzo[b]azepine-9-carboxamide (6)** To a solution of 2,3,4,5-tetrahydro-1H-benzo[b]azepine-9-carboxylic acid (**5,** 0.5 g, 2.61 mmol) in dichloromethane (10 mL) were added 2M methyl amine (1.9 mL, 3.92 mmol), N-(3-dimethylaminopropyl)-N′-ethylcarbodiimide hydrochloride (1.25 g, 6.53 mmol), triethyl amine (1.1 mL, 7.84 mmol) and hydroxybenzotriazole (0.2 g, 1.3 mmol). The reaction mixture was stirred at room temperature for 16 h. The reaction mixture was basifyed using sodium bicarbonate solution and was extracted with dichloromethane. The combined organic layer washed with brine solution and dried over anhydrous sodium sulphate, concentrated solvent under reduced pressure to give crude residue which was purified by combi flash purifier using 18% ethyl acetate/hexane as eluent to give the title compound as a white solid (**6,** 0.322 g, 60.3 % yield). ^1^H NMR (CDCl_3,_ 400 MHz) δ 1.69-1.71 (m, 2H), 1.76-1.79 (m, 2H), 2.80 (t, *J* = 5.2 Hz, 2H), 2.96 (d, *J* = 4.8 Hz, 2H), 3.10 (t, *J* = 4.8 Hz, 2H), 6.03 (bs, 1H), 6.69 (t, *J* = 7.6 Hz, 1H), 7.04 (s, 1H), 7.14-7.19 (m, 2H). MS (ESI) *m/z* 205.1 (M+H) ^+^.

**Synthesis of N-methyl-1-(2,3,4,5-tetrahydro-1H-benzo[b]azepin-9-yl)methanamine (7)** To a solution of N-methyl-2,3,4,5-tetrahydro-1H-benzo[b]azepine-9-carboxamide (**6,** 0.32 g, 1.56 mmol) in tetrahydrofuran (15 mL) was cooled to 0 °C, then 1M LAH solution in tetrahydrofuran (7.8 mL, 7.83 mmol) was added and the reaction mixture was heated at 65 °C for 16 h. The reaction mixture was cooled to room temperature and slowly quenched with ammonium chloride solution at 0 °C, add ethyl acetate and stirred for 0.5 h. The precipitate was filtered, the filtrate was extracted. The combined organic layer washed with brine solution and dried over anhydrous sodium sulphate, concentrated solvent under reduced pressure to give crude residue which was used next step without any purification (**7,** 0.28 g, crude). MS (ESI) *m/z* 191.1 (M+H)^+.^

**Synthesis of 2-methyl-1,2,5,6,7,8-hexahydro-3H-azepino[3,2,1-ij]quinazolin-3-imine (Compound 5)** To a solution of N-methyl-1-(2,3,4,5-tetrahydro-1H-benzo[b]azepin-9-yl)methanamine (0.28 g, 1.47 mmol) in ethanol (5 mL) was added cyanogen bromide (0.187 g, 1.76 mmol). The reaction mixture was heated at 80 °C for 1 h. The reaction mixture was cooled to room temperature and concentrated solvent completely give crude residue which was purified using which was purified by combi flash purifier using 5% methanol/dichloromethane as eluent to give the title **Compound (5)** as a white solid (0.165 g, 38 % yield). ^1^H NMR (DMSO-*d_6_*_,_ 400 MHz) δ 1.76-1.77 (m, 2H), 1.93 (s, 2H), 2.83 (t, *J* = 5.2 Hz, 2H), 3.10 (s, 3H), 3.84 (s, 2H), 4.39 (s, 2H), 7.04-7.15 (m, 3H), 8.02 (bs, 2H). 13C NMR (DMSO-d6) δ 156.05, 135.62, 131.34, 130.38, 125.94, 125.21, 123.52, 49.63, 49.53, 37.46, 31.12, 25.59, 23.76. MS (ESI) *m/z* 216.1 (M+H)^+^-HBr. HPLC purity @ 254 nm, 99.68 %.

**Compound (6)**

**Synthesis of 1-(1H-indol-7-yl)-N-methylmethanamine (2)-** To a solution of 1H-indole-7-carbaldehyde (**1**, 15.0 g, 103.4 mmol) in MeOH (200 mL) was added methylamine (40% in water), (17.1 mL, 155.1 mmol) at 0°C, and stirred for 1.5 h at room temperature. Then sodium borohydride (4.9 g, 129.2 mmol) was added by lot’s wise over 15 min at 0°C. The reaction mixture was stirred for 1 h at RT. The reaction mixture was concentrated under reduced pressure, and added water (10 mL), then extracted in to EtOAc, Organic layer was washed with water and brine, dried over sodium sulphate, filtered and concentrated under reduced pressure to afford the desired product (**2**) as a light green semi solid (15.0 g, 90.6 %). MS (ESI) *m/z* 161.1 (M+H)+.

# Synthesis of 1-(indolin-7-yl)-N-methylmethanamine (3)-To a solution of 1-(1H-indol-7-yl)-N-methylmethanamine (2, 15.0 g, 93.62 mmol) in TFA (100 mL) was added triethyl silane (22.3 mL, 140.4 mmol) at 0 °C and the reaction mixture was stirred at RT for 2 h. The reaction mixture was concentrated under reduced pressure, and added water (10 mL), then extracted in to EtOAc, Organic layer was washed with saturated NaHCO_3_ solution and brine, dried over sodium sulphate, filtered and concentrated under reduced pressure to afford the desired product (3) as a light green semi solid (15.0 g, 98.8 %). MS (ESI) *m/z* 163.2 (M+H)+.

**Synthesis of 2-methyl-1,2,5,6-tetrahydro-3H-pyrrolo[3,2,1-ij]quinazolin-3-imine hydrobromide (Compound 6)**-To a solution of 1-(indolin-7-yl)-N-methylmethanamine (**3**, 16 g, 98.76 mmol) in ethanol (100 mL) was added cyanogen bromide (11.51g, 108.6 mmol) at RT and the reaction mixture was stirred at 85 °C for 2 h. The reaction mixture was concentrated under reduced pressure. The crude was washed with ether twice and with acetonitrile twice. The obtained crude was purified by combi-flash purifier with 1-10% MeOH in DCM as eluent to afford the desired product (**Compound** **6**) as off-white solid (16 g, 60%). ^1^H NMR (DMSO-d_6_, 400 MHz) δ 3.05 (s, 3H), 3.28 (t, *J* = 8.0 Hz, 2H), 4.05 (t, *J* = 8.4 Hz, 2H), 4.64 (s, 2H), 7.05-6.99 (m, 2H), 7.19 (d, *J* = 7.2 Hz, 1H), 8.02 (s, 2H); 13C NMR (DMSO-d6) δ 150.81, 136.55, 128.52, 124.70, 122.93, 114.29, 116.19, 50.46, 48.00, 36.83, 27.93. HPLC Purity @ 280 nm, 99.74%. MS (ESI) *m/z* 188.2 (M+H)+.

**Compound-(7)**

**Synthesis of 1-(1H-indol-7-yl)-N-methylmethanamine (2)-** A solution of 1H-indole-7-carbaldehyde (**1**, 1 g, 6.80 mmol) in methanol (10 mL) was added 40% methylamine solution in water (1.14 mL, 10.3 mmol) at 0 °C and the reaction mixture was stirred for 40 mins at room temperature followed by added sodium borohydride (0.38 g, 10.3 mmol) at 0 °C and the reaction mixture was further stirred for 30 minutes at room temperature. After completion of the reaction, reaction mixture was concentrated under reduced pressure, to the residue obtained was added water and extracted with DCM and separated the organic layer. Organic layer was washed with water and brine, dried over sodium sulphate, filtered and concentrated under reduced pressure to get desired crude compound. The obtained crude was purified by combi-flash purifier with 5% methanol in DCM as eluent to afford the desired product **(2)** as a pale yellow semi-solid (1.1 g, 90 % yield). ^1^H NMR (DMSO-*d*_6,_ 400 MHz) δ 2.28 (s, 3H), 3.90 (s, 2H), 6.39 (d, *J* = 3.2 Hz, 1H), 6.89-6.92 (m, 1H), 6.98-7.02 (m, 1H), 7.27 (d, *J* = 3.2 Hz, 1H), 7.39 (d, *J* = 7.6 Hz, 1H), 10.87 (bs, 1H); MS (ESI) *m/z* 161.1 (M+H)^+^

**Synthesis of 1-(indolin-7-yl)-N-methylmethanamine (3)**- To a solution of 1-(1H-indol-7-yl)-N-methylmethanamine (**2**, 1 g, 6.20 mmol) in trifluoro acetic acid (10 mL) was added triethyl silane (1.49 mL, 9.30 mmol) at 0 °C and the reaction mixture was stirred for 2 h at room temperature. After completion of the reaction [monitored by TLC as 5% MeOH-DCM as eluent], the reaction mixture was concentrated under reduced pressure, residue obtained was dissolved in ethyl acetate and diluted with water then separated the organic layer. Organic layer was washed with saturated bicarbonate solution and brine, dried over sodium sulphate, filtered and concentrated under reduced pressure to get desired crude compound **(3)** as a yellow sticky solid (1 g crude).^1^H NMR (DMSO-*d*_6,_ 400 MHz) δ 2.93 (t, *J* = 8.8 Hz, 2H), 3.45 (t, *J* = 8.8 Hz, 1H), 3.87 (s, 2H), 6.54 (t, *J* = 7.6 Hz, 1H), 6.93 (d, *J* = 7.6 Hz, 1H), 7.03 (d, *J* = 7.2 Hz, 1H); MS (ESI) *m/z* 163.1 (M+H)^+^

**Synthesis of 2-methyl-1,2,5,6-tetrahydro-3H-pyrrolo[3,2,1-ij]quinazolin-3-one (Compound 7)**-

A solution of 1-(indolin-7-yl)-N-methylmethanamine (**3**, 1 g, 6.10 mmol) in THF (20 mL) was added CDI (1.5 g, 9.20 mmol) and the reaction mixture was stirred for 12 h at 60 °C. After stirring 12 h, the reaction mixture was poured into water and extracted with ethyl acetate and separated the organic layer. Organic layer was washed with water and brine, dried over sodium sulphate, filtered and concentrated under reduced pressure to get desired compound. The obtained crude was purified by combi-flash purifier with 25% ethyl acetate in hexane as eluent to afford the desired product **(Compound 7)** as an off-white solid (0.35 g, 30 % yield). ^1^H NMR (DMSO-*d*_6,_ 400 MHz) δ 2.87 (s, 3H), 3.09 (t, *J* = 8.4 Hz, 2H), 3.81 (t, *J* = 8.4 Hz, 2H), 4.45 (s, 2H), 6.78-6.86 (m, 1H), 6.92 (d, *J* = 7.6 Hz, 1H), 7.05 (d, *J* = 7.6 Hz, 1H); MS (ESI) *m/z* 189.1 (M+H)^+^; HPLC Purity @ 280 nm, 99.84%.

# Compound (8)

#

**Synthesis of 5-fluoroindoline (2)-**To a solution of 5-fluoro-1H-indole (**1**, 10 g, 74.07 mmol) in acetic acid (50 mL) was added sodium cyano borohydride (13.99 g, 222.2 mmol) at 0 °C under N_2_ atmosphere. Then the reaction mixture was stirred for 16 h at RT. The reaction mixture was concentrated under reduced pressure and quenched with water, extracted with ethyl acetate, washed with 2N NaOH. The organic layer was washed with brine solution and dried over anhydrous sodium sulphate, concentrated under reduced pressure to give the title compound (**2**) as a light yellow solid (5.5 g, 54.2 %). MS (ESI) *m/z* 138.0 (M+H)+.

**Synthesis of tert-butyl 5-fluoroindoline-1-carboxylate (3)-**To a solution of 5-fluoroindoline (**2**, 6 g, 138.6 mmol) in DCM (200 mL) was added triethylamine (16.02 mL, 115.1 mmol) and DMAP (0.534 g, 4.37 mmol) at RT. Then di-tert-butyl dicarbonate (10.56 mL, 46.04 mmol) was added. The reaction mixture was stirred for 16 h at RT. The reaction mixture was quenched with water and extracted with dichloromethane. The organic layer was washed with brine solution and dried over anhydrous sodium sulphate, concentrated under reduced pressure to give the title compound (**3**) as an off-white solid (8 g, 77.1 %). ^1^H NMR (CDCl_3_, 400 MHz) δ 1.55 (s, 9H), 3.08 (t, *J* = 8.4 Hz, 2H), 4.0 (t, *J* = 8.0 Hz, 2H), 6.85 (t, *J* = 8.4 Hz, 2H), 7.77 (bs, 1H).

**Synthesis of 1-(tert-butoxycarbonyl)-5-fluoroindoline-7-carboxylic acid (4)-**To a solution of tert-butyl 5-fluoroindoline-1-carboxylate (**3**, 5 g, 21.07 mmol) in diethyl ether (20 mL) was added TMEDA (4.1 mL, 27.39 mmol) at -78°C under N_2_ atmosphere. Then s-BuLi (18 mL, 25.28 mmol) was added, stirred for 1 h at -78°C and quenched with dry ice pieces directly in to the reaction mixture. The mixture was stirred for 15 min at -78 °C and the allowed to RT. The reaction mixture was stirred for 3 h at RT. The reaction mixture was quenched with water and concentrated under reduced pressure. The crude was washed with ether and the aqueous layer was acidified with saturated citric acid solution to pH~6.0. The obtained solid was filtered and dried under reduced pressure to give the title compound (**4**) as a light yellow solid (5 g, 84.4 %). MS (ESI) *m/z* 280.1 (M-H)+.

**Synthesis of tert-butyl 5-fluoro-7-(methyl carbonyl) indoline-1-carboxylate (5)-** To a solution of 1-(tert-butoxycarbonyl)-5-fluoroindoline-7-carboxylic acid (**4**, 1 g, 3.55 mmol) in dichloromethane (30 mL) was added 2M methyl amine (2.66 mL, 5.33 mmol), N-(3-dimethylaminopropyl)-N′-ethylcarbodiimide hydrochloride (1.02 g, 5.32 mmol), HOAt (6.5 mL, 3.9 mmol), N-methyl morpholine (1.2 mL, 10.65 mmol)*.* The reaction mixture was stirred at RT for 2 h. The reaction mixture was basified using sodium bicarbonate solution and was extracted with dichloromethane. The combined organic layer washed with brine solution and dried over anhydrous sodium sulphate, concentrated solvent under reduced pressure to give crude residue which was purified by column chromatography over silica gel using 10-55% ethyl acetate and hexane as eluent to give the title compound (**5**) as a white solid (0.55 g, 55 %). MS (ESI) *m/z* 295.1 (M+H)+.

**Synthesis of 5-fluoro-N-methylindoline-7-carboxamide (6)-** To a solution of tert-butyl 5-fluoro-7-(methylcarbamoyl)indoline-1-carboxylate (**5**, 0.55 g, 1.87 mmol) in dry dichloromethane (20 mL) was added TFA (0.71 mL, 9.35 mmol) at 0 °C and the reaction mixture was stirred at RT for 1h. The reaction mixture was concentrated under reduced pressure, added water (10 mL), then extracted with EtOAc. Organic layer was washed with saturated NaHCO_3_ solution and brine, dried over sodium sulphate, filtered and concentrated under reduced pressure to afford the desired product (**6**) as an off-white solid (0.45 g crude). MS (ESI) *m/z* 195.1 (M+H)+.

**Synthesis of 1-(5-fluoroindolin-7-yl)-N-methylmethanamine (7)-**To a solution of 5-fluoro-N-methylindoline-7-carboxamide (**6**, 0.45 g, 2.31 mmol) in tetrahydrofuran (20 mL) was cooled to 0 °C, then BH_3_ DMS solution (2.2 mL, 23.16 mmol) was added and the reaction mixture was heated at 65 °C for 16 h. The reaction mixture was cooled to room temperature and slowly quenched with MeOH at 0 °C. The reaction mixture was concentrated under reduced pressure, added water (10 mL), then extracted with EtOAc, Organic layer was washed with cold water and brine, dried over sodium sulphate, filtered and concentrated under reduced pressure to afford the desired product (**7**) as a light brown liquid (0.1 g, 24%). MS (ESI) *m/z* 181.1 (M+H)+.

**Synthesis of 8-fluoro-2-methyl-1,2,5,6-tetrahydro-3H-pyrrolo[3,2,1-ij]quinazolin-3-imine hydro bromide (Compound 8)-**To a solution of 1-(5-fluoroindolin-7-yl)-N-methylmethane amine (**7**, 0.1 g, 0.55 mmol) in ethanol (10 mL) was added cyanogen bromide (0.07 g, 0.66 mmol).Then reaction mixture was heated at 80 °C for 1 h. The reaction mixture was cooled to RT and concentrated solvent completely give crude residue which was purified by column chromatography over silica gel using 1-10 % methanol and dichloromethane as eluent to afford the desired product (**Compound 8**) as an off-white solid (0.029 g, 18.3 %). ^1^H NMR (DMSO-d_6_, 400 MHz) δ 3.03 (s, 3H), 4.07 (t, *J* = 8.0 Hz, 2H), 4.62 (s, 2H), 6.93 (d, *J* = 8.8 Hz, 1H), 7.09 (d, *J* = 8.8 Hz, 1H), 8.01 (s, 2H); ^13^C NMR (DMSO-d_6_) δ 159.66, 150.64, 132.95, 130.67, 115.23, 111.88, 109.97, 50.27, 48.4, 36.79, 28.02.MS (ESI) *m/z* 206.1 (M+H)+-HBr. HPLC purity 99.1 %.

**Compound (9)**

**Synthesis of 8,9-difluoro-2-methyl-1,2,5,6-tetrahydro-3H-pyrrolo[3,2,1-ij]quinazolin-3-imine hydrobromide (9)**

# Compound (9) was synthesized similar to the procedure described for Compound (8). A white solid (0.227 g, 49.4 %). ^1^H NMR (DMSO-*d_6_*_,_ 400 MHz) δ 3.06 (s, 3H), 3.23 (t, *J* =14 Hz, 2H), 4.06 (t, *J* = 8 Hz, 2H), 4.74 (s, 2H), 7.34 (t, *J* = 8.4 Hz, 1H), 8.14 (bs, 2H). ^13^C NMR (DMSO-d_6_) δ 150.51, 146.06, 143.57, 133.24, 124.15, 113.52, 103.92, 48.84, 46.17, 36.88, 27.69. MS (ESI) *m/z* 224.1 (M+H) ^+^-HBr. HPLC purity @ 270 nm, 99.28 %

**Compound (10)**

**Synthesis of tert-butyl 5-fluoro-7-formylindoline-1-carboxylate (2)-** To a solution of tert-butyl 5-fluoroindoline-1-carboxylate (**1,** 5.3 g, 22.36 mmol) in ether (200 mL) was added TMEDA (6.7 mL, 44.72 mmol) at room temperature under N_2_ atmosphere. Then s-BuLi (18 mL, 42.48 mmol) was added at -78 °C. Then the reaction mixture was stirred for 2 h at -78 °C and DMF was added to the reaction mixture and stirred for 2 h at -78 °C. The reaction mixture was quenched with saturated NH_4_Cl then extracted with EtOAc. The organic layer was washed with cold water and brine, dried over sodium sulphate, filtered and concentrated under reduced pressure. The obtained crude was purified by combi-flash purifier with 2-10% ethyl acetate in hexane as eluent to afford the desired product **(2)** as a yellow solid (3.4 g, 57.4 %). ^1^H NMR (DMSO-d_6_, 400 MHz) δ 1.43 (s, 9H), 3.1 (t, *J* = 8.0 Hz, 2H), 4.12 (t, *J* = 8.0 Hz, 2H), 7.18 (t, *J* = 6.8 Hz, 1H), 7.4 (t, *J* = 8.0 Hz, 1H), 9.91 (s, 1H);

**Synthesis of tert-butyl 7-((ethylamino)methyl)-5-fluoroindoline-1-carboxylate (3)-** To a solution of tert-butyl 5-fluoro-7-formylindoline-1-carboxylate (**2,** 1.0 g, 3.77 mmol) in MeOH (20 mL) was added ethylamine (70% in water), (0.45 mL, 5.66 mmol) at 0 °C, and stirred for 2 h at room temperature. Then sodium borohydride (0.17 g, 4.52 mmol) was added by lot’s wise over 15 mins at 0 °C. The reaction mixture was stirred for 1 h at room temperature. The reaction mixture was concentrated under reduced pressure, and added water (10 mL), then extracted into EtOAc, Organic layer was washed with water and brine, dried over sodium sulphate, filtered and concentrated under reduced pressure to afford the desired product **(3)** as a yellow liquid (1.0 g crude). ^1^H NMR (DMSO-d_6_, 400 MHz) δ 3.03 (s, 3H), 4.07 (t, *J* = 8.0 Hz, 2H), 4.62 (s, 2H), 6.93 (d, *J* = 8.8 Hz, 1H), 7.09 (d, *J* = 8.8 Hz, 1H), 8.01 (s, 2H); MS (ESI) *m/z* 206.1 (M+H)+-HBr. HPLC purity 99.1 %.

**Synthesis of N-((5-fluoroindolin-7-yl) methyl)ethanamine (4)-** To a solution of tert-butyl 7-((ethylamino)methyl)-5-fluoroindoline-1-carboxylate (**3,** 1.1 g crude, 3.74 mmol) in DCM (10 mL) was added TFA (2.86 mL, 37.4 mmol) at 0 °C and the reaction mixture was stirred at RT for 16 h. The reaction mixture was concentrated under reduced pressure, and added water (10 mL), then extracted into EtOAc, Organic layer was washed with saturated NaHCO_3_ solution and brine, dried over sodium sulphate, filtered and concentrated under reduced pressure to afford the desired product **(4)** as a brown solid (1 g crude).^1^H NMR (CDCl_3_, 400 MHz) δ 1.17 (t, *J* = 9.6 Hz, 3H), 3.02 (t, *J* = 8.4 Hz, 2H), 2.74 (q, *J* = 7.2 Hz, 2H), 3.59 (t, *J* = 7.8 Hz, 2H), 3.72 (s, 2H), 6.64 (d, *J* = 9.2 Hz, 1H), 6.78 (d, *J* = 8.0 Hz, 1H).

**Synthesis of 2-ethyl-8-fluoro-1,2,5,6-tetrahydro-3H-pyrrolo[3,2,1-ij]quinazolin-3-imine hydrobromide (Compound 10)-** To a solution of N-((5-fluoroindolin-7-yl)methyl)ethanamine (**4,** 1 g crude, 5.15 mmol) in ethanol (30 mL) was added cyanogen bromide (0.6 g, 5.67 mmol). Then the reaction mixture was heated at 80 °C for 2 h. The reaction mixture was cooled to room temperature and concentrated solvent completely give crude residue was washed with ether twice and with ACN twice, filtered, dried under reduced pressure to give the title **Compound (10)** as a yellow solid (0.32 g, 20.7 %).^1^H NMR (DMSO-d_6_, 400 MHz) δ 1.18 (t, *J* = 6.8 Hz, 3H), 3.48 (q, *J* = 7.2 Hz, 2H), 4.07 (t, *J* = 8.0 Hz, 2H), 4.66 (s, 2H), 6.93 (d, *J* = 9.2 Hz, 1H), 7.09 (d, *J* = 8.8 Hz, 1H), 8.0 (s, 2H); 13C NMR (DMSO-d6) δ 159.7, 149.9, 132.98, 130.63, 115.19, 111.88, 110.05, 48.45, 47.94, 44.08, 28.01, 10.96. HPLC purity 99.1 %.MS (ESI) *m/z* 220.1 (M+H)^+^-HBr.

# Compound (11)

#

# Synthesis of 7-bromo-4-fluoro-1H-indole (2)-To a solution of 1-bromo-4-fluoro-2-nitrobenzene (1, 5 g, 22.72 mmol) in tetrahydrofuran (50 mL) was added vinylmagnesium bromide (136.36 mL, 136.36 mmol) at -78 °C under N_2_ atmosphere. Then the reaction mixture was stirred for 4 h at -78 °C. The reaction mixture was quenched with saturated NH_4_Cl then extracted with EtOAc, Organic layer was washed with cold water and brine, dried over sodium sulphate, filtered and concentrated under reduced pressure. The obtained crude was purified by combi-flash purifier with 1-10% ethyl acetate in hexane as eluent to afford the title compound (2) as an off-white solid (2.5 g, 51 % yield). MS (ESI) *m/z* 213.9 (M-H)+.

# Synthesis of 7-bromo-4-fluoroindoline (3)-To a solution of 7-bromo-4-fluoro-1H-indole (2, 2.3 g, 10.74 mmol) in trifluoro acetic acid (25 mL) was added triethyl silane (4.45 mL, 27.94 mmol) at 0 °C and the reaction mixture was stirred at room temperature for 2 h. The reaction mixture was concentrated under reduced pressure, and added water (10 mL), then extracted into ethyl acetate, Organic layer was washed with saturated sodium bicarbonate solution and brine, dried over sodium sulphate, filtered and concentrated under reduced pressure. The obtained crude was purified by combi-flash purifier with 1-5 % ethyl acetate in hexane as eluent to afford the desired product (3) as an off-white solid (1.8 g, 77.5 % yield). MS (ESI) *m/z* 218.0 (M+H)+.

# Synthesis of tert-butyl 7-bromo-4-fluoroindoline-1-carboxylate (4) -To a solution of 7-bromo-4-fluoroindoline (3, 1.5 g, 6.94 mmol) in dichloromethane (50 mL) was added diisopropyl ethyl amine (3.63 mL, 20.82 mmol) and 4-dimethylaminopyridine (0.084 g, 0.694 mmol) at rt. Then di-tert-butyl dicarbonate (1.67 mL, 7.25 mmol) was added. The reaction mixture was stirred for 16 h at room temperature. The reaction mixture was quenched with water and extracted with dichloromethane. The organic layer was washed with brine solution and dried over anhydrous sodium sulphate, concentrated under reduced pressure. The obtained crude was purified by combiflash purifier with 1-20 % ethyl acetate in hexane as eluent to afford the desired product (4) as an off-white solid (1.4 g, 64 % yield). ^1^H NMR (CDCl_3_, 400 MHz) δ 1.54 (s, 9H), 3.06 (t, *J* = 7.6 Hz, 2H), 4.15 (t, *J* = 7.6 Hz, 2H), 6.67 (t, *J* = 8.0 Hz, 1H), 7.33-? (m, 1H);

# Synthesis of 8-fluoro-2-methyl-1,2,5,6-tetrahydro-3H-pyrrolo[3,2,1-ij]quinazolin-3-imine hydrobromide Compound (11)--The intermediate 4 was converted to compound 11 using the similar procedure described for Compound 8- title compound obtained as a white solid (11) (0.095 g, 40% yield). ^1^H NMR (DMSO-d_6_, 400 MHz) δ 3.05 (s, 3H), 3.32 (m, 2H), 4.1 (t, *J* = 8.0 Hz, 2H), 4.62 (s, 2H), 6.88 (t, *J* = 8.8 Hz, 1H), 7.09 (t, *J* = 7.2 Hz, 1H), 8.01 (s, 2H); HPLC purity 99.46 %. MS (ESI) *m/z* 206.1 (M+H)+ -HBr.

# Compound (12)

#

**Synthesis of 2-amino-3-hydroxybenzoic acid (1)-**To a solution of 2-amino-3-methoxybenzoic acid (SM, 33.4 g, 0.2 mol) in toluene (1000 mL) was added AlCl_3_ (133 g ,1.0 mol). The reaction mixture was stirred at 105 ^o^C for 5 hrs. After cooling to room temperature, water was added, adjusted pH to 3-4, filtered and the brown solid was washed with water and then dried under vacuum to get intermediate **1** as brown solid (20.15 g, 65.4% yield).

**Synthesis of methyl 2-amino-3-hydroxybenzoate (2)-**To a solution of **1** (20.0 g, 0.13 mol) in anhydrous methanol (300 mL ) was added dropwise thionyl chloride (30 mL, 0.4 mol) at room temperature over 1 h. The mixture was refluxed for 3 hours (gas trap). The solvent was removed under vacuum and the residue was taken in dichloromethane (400 mL), washed with saturated aqueous sodium bicarbonate (200 mL x 2), brine and dried over sodium sulphate. After filtration, the filtrate was concentrated to yield the desired intermediate **2** as a solid (18.8 g, 86.6% yield).

**Synthesis of methyl 3-oxo-3,4-dihydro-2H-benzo[b][1,4]oxazine-5-carboxylate (3)-** To a solution of **2** (18.4 g, 110 mmol) in CH_3_CN (300 ml) was added NEt_3_, the mixture was cooled to -10 ℃ and 2-chloroacetyl chloride was added dropwise. Then the mixture was stirred at room temperature overnight. The solvent was concentrated, and the residue dissolved in water (300 mL) and acidified with 1M HCl. The mixture was extracted with ethyl acetate (150 mL X 3), the combined organic layer was dried over Na_2_SO_4_, filtered and concentrated to afford intermediate **3** (20.2 g, 88.7% yield).

**Synthesis of N-methyl-3-oxo-3,4-dihydro-2H-benzo[b][1,4]oxazine-5-carboxamide (4)-**A mixture of intermediate **3** (20.0 g) and 40% methylamine in water (1000 mL) is heated to 70 ^o^C .in a sealed flask overnight. The resulting solution was concentrated and dried in vacuo to afford the intermediate **4** (17.0 g, 85% yield) which was used in next step without further purification.

**Synthesis of 1-(3,4-dihydro-2H-benzo[b][1,4]oxazin-5-yl)-N-methylmethanamine (5)-**To a solution of intermediate **4** (16.0 g, 77.7 mmol) in anhydrous THF (400 mL) was added LiAlH_4_ (30 g, 0.81 mol) in 1 h. The mixture was refluxed for 48 hrs (gas trap). The mixture was diluted with water and acidified with 2M HCl. The mixture was extracted with CH_2_Cl_2_ (200 ml*3), the combined organic layer was dried over Na_2_SO_4_, filtered and concentrated to afford intermediate **5** (15.4 g) which was used in next step without further purification.

**Synthesis of 6-methyl-2,3,6,7-tetrahydro-5H-[1,4]oxazino[2,3,4-ij]quinazolin-5-imine (Compound 12)-**The crude intermediate **5** (15.4 g) was dissolved in ethanol (300 mL), then cyanogen bromide was added, and the reaction mixture was stirred at 85 ^o^C for 4 hrs. After cooling to room temperature, the ethanol was evaporated to give crude product which was purified by column to afford the title **Compound 12** (6.80 g, 43% yield for 2 steps) as a brown solid (HBr salt). ^1^H NMR (DMSO-*d*_6,_ 400 MHz) δ 3.15 (s, 3H), 3.95 (m, 2H), 4.30 (m, 2H), 4.60 (s, 2H), 6.70 (m, 1H), 6.90 (m, 1H), 7.10 (m, 1H), 8.15 (bs, 2H); ^13^C NMR (DMSO-d6) δ 151.50, 124.80, 121.0, 120.50, 117.50, 116.0, 63.50, 48.70, 43.0, 38,0; MS (ESI) *m/z* 204.1 (M+H)^+^.

**Compound (13)**

**Synthesis of 3, 4-dihydro-2H-benzo[b][1,4]thiazine (2)-** To a solution of 2-aminobenzenethiol (**1**, 10.0 g, 80.0mmol) in acetone (100 mL) was added potassium carbonate (33.12 g, 240.0 mmol) at 0 °C followed by 1,2-dibromoethane (8.5 mL, 96.0 mmol) and stirred for 12h at room temperature. After completion of the reaction [monitored by TLC, ethyl acetate: hexane (2:8)]. The reaction mixture was filtered through diatomaceous earth; Organic layer concentrated under reduced pressure. The obtained crude was purified by combi-flash purifier with 10% ethyl acetate in hexane as eluent to afford the desired product **(2)** as a yellow oil (4.2 g, 35 %). ^1^H NMR (DMSO-d_6,_ 400 MHz) δ 2.92-2.94 (m, 2H), 3.44 (t, *J* = 4.8 Hz, 2H), 5.95 (bs, 1H), 6.39-6.47 (m, 2H), 6.75-6.81 (m, 2H); MS (ESI) *m/z* 152.1 (M+H)^+^.

**Synthesis of 2, 3-dihydro-[1, 4] thiazino [2, 3, 4-hi] indole-5, 6-dione (3)** To a solution of 3,4-dihydro-2H-benzo[b][1,4]thiazine (**2**, 1.0 g, 6.62 mmol) in tetrahydrofuran (10 mL) was added oxalyl chloride (1.12 mL, 13.24 mmol) at 0 °C, over the period of 10 mins and the mixture was boiled under reflux for 3 h, and then concentrated under reduced pressure. The resulting mixture was dissolved into CS_2_ (10.0 mL) and AlCl_3_(1.8 g, 13.24 mmol) was added portion wise over the period of 10 mins, when addition was completed the reaction mixture was boiled under reflux an additional 12h, After cooling and decantation of CS_2_ solution 0.5N aqueous HCl solution was added at 0 °C to the residue and the mixture was stirred until precipitation. The solid was filtered and washed with water, pentane, and ether to afford the desired product **(3)** as a purple powder (1.1 g, 81%). ^1^H NMR (DMSO-*d*_6,_ 400 MHz) δ 3.16 (t, *J* = 4.8 Hz, 2H), 3.87 (t, *J* = 4.8 Hz, 2H), 7.03-7.11 (m, 1H), 7.29 (d, *J* = 7.6 Hz, 1H), 7.45 (d, *J* = 8.0 Hz, 1H); MS (ESI) *m/z* 206.1 (M+H)^+^.

**Synthesis of 3, 4-dihydro-2H-benzo[b] [1, 4] thiazine-5-carboxylic acid (4)** To a solution of 2,3-dihydro-[1,4]thiazino[2,3,4-hi]indole-5,6-dione (**3**, 1.0 g, 4.87 mmol) in water (10.0 mL) was added 30%aqueous NaOH Solution (20.0 mL), then the mixture was stirred at 65 °C for 48 h. After completion of the reaction [monitored by TLC, ethyl acetate: hexane (5:5)]. The reaction mixture was acidified with conc.HCl and extracted into ethyl acetate. The organic layer was washed with water and brine, dried over sodium sulphate, filtered and concentrated under reduced pressure to get desired crude compound. The obtained crude was purified by combi-flash purifier with 20%ethyl acetate in hexane eluent to afford the desired product **(4)** as a yellow solid (0.18 g, 18 %). ^1^H NMR (DMSO-*d*_6,_ 400 MHz) δ 2.95 (t, *J* = 4.8 Hz, 2H), 3.67 (t, *J* = 4.8 Hz, 2H), 6.44 (t, *J* = 7.6 Hz, 1H), 7.10 (d, *J* = 7.2 Hz, 1H), 7.55 (d, *J* = 8.0 Hz, 1H), 8.24 (bs, 1H), 12.80 (bs, 1H); MS (ESI) *m/z* 196.1 (M+H)^+^.

**Synthesis of N-methyl-3, 4-dihydro-2H-benzo[b] [1, 4] thiazine-5-carboxamide (5)-** To a solution of 3,4-dihydro-2H-benzo[b][1,4]thiazine-5-carboxylic acid (**4**, 0.18 g, 0.92mmol) in dichloromethane (5.0 mL) was added triethylamine (0.27 mL, 2.76 mmol) at 0 °C followed by 2M methylamine solution in THF (0.50 mL, 1.10 mmol) and 1-propane phosphoric acid cyclic anhydride (0.87 mL, 2.76 mmol), and stirred for 12 h at room temperature, reaction mixture was concentrated under reduced pressure, and added water (10 mL), then extracted in to EtOAc, Organic layer was washed with saturated NaHCO_3_ solution and brine, dried over sodium sulphate, filtered and concentrated under reduced pressure. The obtained crude was purified by combi-flash purifier with 20% ethyl acetate in hexane as eluent to afford the desired product **(5)** as a yellow liquid (0.18 g, 93 %). ^1^H NMR (DMSO-*d*_6,_ 400 MHz) δ 2.70 (d, *J* = 3.6 Hz, 3H), 2.93 (t, *J* = 4.8 Hz, 2H), 3.58 (t, *J* = 2.4 Hz, 2H), 6.44 (t, *J* = 7.6 Hz, 1H), 6.98 (d, *J* = 7.6 Hz, 1H), 7.22 (d, *J* = 7.2 Hz, 1H), 8.05 (s, 1H), 8.22 (bs, 1H); MS (ESI) *m/z* 209.1 (M+H)^+^.

**Synthesis of 1-(3, 4-dihydro-2H-benzo[b] [1, 4] thiazin-5-yl)-N-methylmethanamine (6)-**

# To a solution of N-methyl-3,4-dihydro-2H-benzo[b][1,4]thiazine-5-carboxamide (5, 0.18 g, 0.96 mmol) in tetrahydrofuran (10.0 mL) was added 2M LiAlH_4_ solution in THF (0.75 mL, 1.44 mmol) at 0 °C and the reaction mixture was stirred at 65 °C for 48h. After refluxing overnight, the reaction mixture was quenched with brine solution at 0 °C and diluted with ethyl acetate. Then it was filtered through diatomaceous earth and the organic layer separated. Organic layer was washed with water and brine, dried over sodium sulphate, filtered and concentrated under reduced pressure to get desired crude compound. The obtained crude was purified by combi-flash purifier with 7% methanol in dichloromethane as eluent to afford the desired product (6) as a viscous oil (0.06 g, 32 %). ^1^H NMR (DMSO-*d*_6,_ 400 MHz) δ 2.10 (bs, 1H), 2.22 (s, 1H), 2.93 (t, *J* = 4.8 Hz, 2H), 3.53 (s, 4H), 6.35 (bs, 1H), 6.41 (t, *J* = 7.6 Hz, 1H), 6.76 (t, *J* = 7.6 Hz, 2H); MS (ESI) *m/z* 195.1 (M+H)^+^.

**Synthesis of 6-methyl-2, 3, 6, 7-tetrahydro-5H-[1, 4] thiazino [2, 3, 4-ij] quinazolin-5-imine Hydrobromide (Compound 13)-**To a solution of 1-(3, 4-dihydro-2H-benzo[b][1,4]thiazin-5-yl)-N-methylmethanamine (**6**, 0.06 g, 0.309 mmol) in ethanol (5.0 mL), then cyanogen bromide (0.05 g, 0.46 mmol) was added, and the reaction mixture was stirred at 85 °C for 2 h, after cooled to room temperature reaction mixture was concentrated under reduced pressure to get solid which was filtered through Buckner funnel and then solid was washed with ethanol (2.0 mL),acetonitrile (2.0 mL), pentane and diethyl ether. Finally collect the entire solid to afford **(Compound 13)** (0.045 g, 67%) as a grey solid. ^1^H NMR (DMSO-*d*_6,_ 400 MHz) δ 3.15 (s, 3H),3.24 (s, 2H), 4.05 (bs, 2H), 4.43 (s, 2H), 6.98 (d, *J* = 7.6 Hz, 1H), 7.08 (t, *J* = 7.2 Hz, 1H), 7.19 (d, *J* = 7.2 Hz, 1H), 8.19 (bs, 2H); ^13^C NMR (DMSO-d6) δ 153.33, 130.30, 126.97, 124.72, 123.57, 122.55, 122.19, 49.05, 46.06, 37.97, 25.42. MS (ESI) *m/z* 220.1 (M+H)^+^-HBr;HPLC Purity @ 254nm,98.06%.

**Compound (14)**

**Synthesis of**  ethyl 2, 3-diaminobenzoate **(2)**- To a solution of ethyl 2-amino-3-nitrobenzoate (**1**, 5 g, 23.7 mmol) in ethanol (60 mL) was added Pd/C (10 %) under nitrogen atmosphere and the reaction mixture was stirred at room temperature for 5 h under hydrogen balloon (H_2_). The reaction mixture was filtered through celite washed with methanol, the filtrate was concentrated under reduced pressure to give crude residue which was purified Combi flash purifier using 15% ethyl acetate/hexane as eluent to give the title compound **(2)** as a brown solid (3.3 g, 77.1 %). MS (ESI) *m/z* 287.1 (M+H) ^+^. ^1^H NMR (CDCl_3,_ 400 MHz) δ 1.38 (t, *J* = 6.8 Hz, 3H), 3.34 (bs, 2H), 4.33(q, *J* =6.8 Hz, 2H), 5.45 (bs, 2H), 6.59 (t, *J* = 8.0 Hz, 1H), 6.84 (dd, *J* = 1.6 Hz, *J* = 7.6 Hz, 1H), 7.48 (dd, *J* = 1.2 Hz, *J* = 8.0 Hz, 1H); MS (ESI) *m/z* 181.1 (M+H) ^+^.

**Synthesis of ethyl 3-oxo-1,2,3,4-tetrahydroquinoxaline-5-carboxylate (3)-** To a solution of ethyl 2, 3-diaminobenzoate (**2**, 1.6 g, 8.87 mmol) in water (30 mL) were added 2-chloroacetic acid (1.25 g, 13.3 mmol) and sodium hydroxide (0.71 g, 17.7 mmol). The reaction mixture was stirred at 100 °C for 16 h. The reaction mixture was cooled to room temperature, which was acidified using 1N HCl solution and it was extracted with ethyl acetate. The combined organic layer washed with brine solution and dried over anhydrous sodium sulphate, concentrated solvent under reduced pressure to give crude residue which was purified by Combi flash purifier using 30% ethyl acetate/hexane as eluent to give the title compound **(3)** as a yellow solid (0.6 g, 30.76 %). ^1^H NMR (DMSO *d_6_*_,_ 400 MHz) δ 1.30 (t, *J* = 7.2 Hz, 3H), 3.82 (s, 2H), 4.33(q, *J* = 6.8 Hz, 2H), 6.30 (s, 2H), 6.86 (t, *J* = 8.0 Hz, 1H), 6.91 (d, *J* = 7.6 Hz, 1H), 7.22 (d, *J* = 7.6 Hz, 1H), 10.14 (s, 1H); MS (ESI) *m/z* 221.1 (M+H) ^+^.

**Synthesis of N-methyl-3-oxo-1,2,3,4-tetrahydroquinoxaline-5-carboxamide (4)-** To a solution of ethyl 3-oxo-1, 2, 3, 4-tetrahydroquinoxaline-5-carboxylate (**3**, 0.2 g, 0.908 mmol) in methylamine in water (5 mL) was heated at 80 °C for 16 h in a sealed bomb. The reaction mixture was cooled to room temperature which was concentrated under reduced pressure to give crude residue which was triturated using n-pentane to give the title compound **(4)** as an off yellow solid (0.2 g, crude). MS (ESI) *m/z* 206.1 (M+H) ^+^.

**Synthesis of N-methyl-1-(1,2,3,4-tetrahydroquinoxalin-5-yl)methanamine (5)-** To a solution of N-methyl-3-oxo-1,2,3,4-tetrahydroquinoxaline-5-carboxamide (**4**, 0.2 g, 0.974 mmol) in tetrahydrofuran (10 mL) was cooled to 0 °C, then 1M LAH solution in THF (7.7 mL, 7.79 mmol) was added and the reaction mixture was heated at 68 °C for 48 h. The reaction mixture was cooled to room temperature and slowly quenched with ammonium chloride solution at 0 °C, add ethyl acetate and stirred for 0.5 h. The precipitate was filtered, the filtrate was extracted. The combined organic layer washed with brine solution and dried over anhydrous sodium sulphate, concentrated solvent under reduced pressure to give crude residue which was purified by Combi flash purifier using 8% methanol/dichloromethane as eluent to give the title compound **(5)** as a yellow oily mass (0.07 g, 40.69 %). ^1^H NMR (DMSO *d_6_*_,_ 400 MHz) δ 2.42 (s, 3H), 3.00 (t, *J* = 8.4 Hz, 2H), 3.57 (t, *J* = 8.4 Hz, 2H), 3.64 (s, 3H), 6.61 (d, *J* = 10.0 Hz, 1H), 6.75 (d, *J* = 8.0 Hz, 1H); MS (ESI) *m/z* 178.2 (M+H) ^+^.

**Synthesis of 5-imino-6-methyl-2,3,6,7-tetrahydro-1H,5H-pyrazino[3,2,1-ij]quinazoline-1-carbonitrile hydrobromide (Compound 14)-** To a solution of N-methyl-1-(1,2,3,4-tetrahydro quinoxalin-5-yl)methanamine (**5**, 0.07 g, 0.394 mmol) in ethanol (6 mL) was added cyanogen bromide (0.05 g, 0.473 mmol).Then reaction mixture was heated at 80° C for 2 h. The reaction mixture was cooled to room temperature, the precipitate formed was filtered through sintered funnel and washed with n-pentane and dried completely to give the title **(Compound 14)** as an off-white solid (0.012 g, 9.9 %).

# ^1^H NMR (DMSO-*d_6_*_,_ 400 MHz) δ 3.10 (s, 3H), 3.99 (s, 4H), 4.53 (s, 2H), 6.93 (d, *J* = 7.2 Hz, 1H)*,* 7.07 (d, *J* = 8.0 Hz, 1H), 7.21 (t, *J* = 7.6 Hz, 1H), 8.19 (bs, 2H); MS (ESI) *m/z* 228.1(M+H)^+^-HBr. HPLC purity @ 240nm, 93.7 %.

**Compound (15)**

**Synthesis of methyl 3-fluoro-2-nitrobenzoate (2)** To a solution of 3-fluoro-2-nitrobenzoic acid (**1**, 5 g, 27 mmol) in methanol (60 mL) was cooled to 0 °C, sulfuric acid (14.39 mL, 270 mmol) was added slowly and the reaction mixture was heated at 65 °C for 48 h. The reaction mixture was cooled to room temperature, concentrated solvent completely, basify using sodium bicarbonate solution and was extracted with ethyl acetate. The combined organic layer washed with brine solution and dried over anhydrous sodium sulphate, concentrated solvent under reduced pressure and dried completely to give the title compound **(2)** as an off-white solid (5.2 g, 96.8 % yield). ^1^H NMR (CDCl_3,_ 400 MHz) δ 3.92 (s, 3H), 7.45 (t, *J* = 8.4 Hz, 1H), 7.54-7.60 (m, 1H), 7.80 (d, *J* = 7.6 Hz, 1H).

**Synthesis of methyl 3-(benzylamino)-2-nitrobenzoate (3**)-To a solution of methyl methyl 3-fluoro-2-nitrobenzoate (4.7 g, 23.6 mmol) in tetrahydrofuran (60 mL) was added benzylamine (10 mL) and the reaction mixture was stirred at room temperature for 16 h. The reaction mixture was diluted with water and was extracted with ethyl acetate. The combined organic layer washed with brine solution and dried over anhydrous sodium sulphate, concentrated solvent under reduced pressure to give crude residue which was purified by combi flash purifier using 100% hexane as eluent to give the title compound **(3)** as an orange solid (5.2 g, crude). MS (ESI) *m/z* 287.1 (M+H) ^+^.

**Synthesis of methyl 2-amino-3-(benzylamino)benzoate (4)-** To a solution of methyl 3-(benzylamino)-2-nitrobenzoate (**3**, 5.2 g, 18.1 mmol) in methanol (60 mL) and water (15 mL) were added zinc dust (5.9 g, 90.8 mmol) and ammonium chloride (9.7 g, 181.7 mmol). The reaction mixture was stirred at room temperature for 3 h. The reaction mixture was filtered through celite washed with methanol. The filtrate was concentrated and the crude residue was extracted with ethyl acetate. The combined organic layer washed with brine solution and dried over anhydrous sodium sulphate, concentrated solvent under reduced pressure to give crude residue which was purified by combiflash purifier using 8% ethyl acetate/hexane as eluent to give the title compound **(4)** an off yellow solid (3.5 g, 75.2 % yield). ^1^H NMR (CDCl_3,_ 400 MHz) δ 3.87 (s, 3H), 4.31 (s, 2H), 5.3 (bs, 1H), 6.66 (t, *J* = 8.0 Hz, 1H), 6.81 (d, *J* = 7.6 Hz, 1H), 7.27 (d, *J* = 6.8 Hz, 1H), 7.30-7.40 (m, 4H), 7.45 (d, *J* = 8 Hz, 1H); MS (ESI) *m/z* 257.2 (M+H) ^+^.

**Synthesis of 1-benzyl-3-oxo-1,2,3,4-tetrahydroquinoxaline-5-carboxylic acid (5)-**To a solution of methyl 2-amino-3-(methylamino)benzoate (**4**, 2 g, 7.8 mmol) in water (30 mL) were added 2-chloroacetic acid (1.1 g, 11.7 mmol) and sodium hydroxide (0.624 g, 15.6 mmol). The reaction mixture was stirred at 80 °C for 16 h. The reaction mixture was cooled to room temperature, which was acidified using 1N HCl solution (p^H^-3 to 4), the solid precipitated, it was filtered and washed with cold water and dried completely to give the title compound **(5)** as a brown solid (2 g, crude). MS (ESI) *m/z* 283.3 (M+H) ^+^.

**Synthesis of 1-benzyl-N-methyl-3-oxo-1,2,3,4-tetrahydroquinoxaline-5-carboxamide (6)-**To a solution of 1-benzyl-3-oxo-1,2,3,4-tetrahydroquinoxaline-5-carboxylic acid (**5**, 1.5 g, 5.31 mmol) in dichloromethane (30 mL) were added 2M methyl amine (4 mL, 7.97 mmol), N-(3-dimethylaminopropyl)-N′-ethylcarbodiimide hydrochloride (2.5 g, 13.2 mmol), triethyl amine (2.27 mL, 15.9 mmol) and hydroxybenzotriazole (0.4 g, 2.65 mmol). The reaction mixture was stirred at room temperature for 16 h. The reaction mixture was basified using sodium bicarbonate solution and was extracted with dichloromethane. The combined organic layer washed with brine solution and dried over anhydrous sodium sulphate, concentrated solvent under reduced pressure to give crude residue which was purified by combi flash purifier using 20% ethyl acetate/hexane as eluent to give the title compound **(6)** as a brown oily mass (0.62 g, crude).MS (ESI) *m/z* 296.2 (M+H) ^+^.

# Synthesis of 1-(1-benzyl-1,2,3,4-tetrahydroquinoxalin-5-yl)-N-methylmethanamine (7)-To a solution of 1-benzyl-N-methyl-3-oxo-1,2,3,4-tetrahydroquinoxaline-5-carboxamide (6, 0.85 g, 2.89 mmol) in tetrahydrofuran (25 mL) was cooled to 0 °C, then 1M LAH solution in tetrahydrofuran (14.47 mL, 14.4 mmol) was added and the reaction mixture was heated at 68 °C for 16 h. The reaction mixture was cooled to room temperature and slowly quenched with ammonium chloride solution at 0 °C Ethyl acetate was added and it was and stirred for 0.5 h. The precipitate was filtered; the filtrate was extracted. The combined organic layer washed with brine solution and dried over anhydrous sodium sulphate, concentrated solvent under reduced pressure to give crude residue which was purified by combiflash purifier using 20% ethyl acetate/hexane as eluent to give the title compound (7) as a yellow oily mass (0.77 g, crude). MS (ESI) *m/z* 268.2 (M+H) ^+^.

# Synthesis of tert-butyl 4-benzyl-8-(((tert-butoxycarbonyl)(methyl)amino)methyl)-3,4-dihydroquinoxaline-1(2H)-carboxylate (8)- To a solution of 1-(1-benzyl-1,2,3,4-tetrahydroquinoxalin-5-yl)-N-methylmethanamine (0.65 g, 2.42 mmol) in di-tert-butyl dicarbonate (10 mL) was added and the reaction mixture was heated at 50 °C for 16 h. The reaction mixture was cooled to room temperature and concentrated completely under reduced pressure to give crude residue which was purified by combi flash purifier using 15% ethyl acetate/hexane as eluent to give (8) as a oily mass (0.75 g, 66.37 % yield). MS (ESI) *m/z* 468.2 (M+H) ^+^.

# Synthesis of tert-butyl 8-(((tert-butoxycarbonyl)(methyl)amino)methyl)-3,4-dihydroquinoxaline-1(2H)-carboxylate (9)- To a solution of tert-butyl 4-benzyl-8-(((tert-butoxycarbonyl)(methyl)amino)methyl)-3,4-dihydroquinoxaline-1(2H)-carboxylate (8, 0.55 g, 1.176 mmol) in methanol (15 mL) was added palladium hydroxide (0.2 g) under nitrogen atmosphere. The reaction mixture was stirred at room temperature for 0.5 h under hydrogen balloon. The reaction mixture was filtered through celite washed with methanol. The filtrate was concentrated under reduced pressure to give crude residue (9) which was used to next step without any purification (0.44 g, 99.3 % yield). ^1^H NMR (CDCl_3,_ 400 MHz) δ 1.46 (s, 18H), 2.76 (s, 3H), 3.42-3.45 (m, 2H), 3.96-4.06 (m, 2H), 4.41-4.66 (m, 2H), 6.43 (d, *J* = 8.0 Hz, 1H), 6.53 (d, *J* = 7.6 Hz, 1H), 6.95 (t, *J* = 7.6 Hz, 1H); MS (ESI) *m/z* 378.4 (M+H) ^+^.

# Synthesis of tert-butyl 4-acetyl-8-(((tert-butoxycarbonyl)(methyl)amino)methyl)-3,4-dihydroquinoxaline-1(2H)-carboxylate (10)- To a solution of tert-butyl 8-(((tert-butoxycarbonyl)(methyl)amino)methyl)-3,4-dihydroquinoxaline-1(2H)-carboxylate (9, 0.25 g, 0.662 mmol) in dichloromethane (10 mL) were added triethylamine (0.186 mL, 1.32 mmol) and acetyl chloride (0.062 g, 0.795 mmol) and the reaction mixture was stirred at room temperature for 1 h. The mixture was diluted with water and was extracted with dichloromethane. The combined organic layer washed with brine solution and dried over anhydrous sodium sulphate, concentrated solvent under reduced pressure to give crude residue which was purified by combi flash purifier using 15% ethyl acetate/hexane as eluent to give the title compound (10) as a sticky mass (0.23 g, 83 % yield) MS (ESI) *m/z* 320.2 (M+H) ^+^-BOC.

# Synthesis of 1-(5-((methylamino)methyl)-3,4-dihydroquinoxalin-1(2H)-yl)ethan-1-one (11)-To a solution of tert-butyl 4-acetyl-8-(((tert-butoxycarbonyl)(methyl)amino)methyl)-3,4-dihydroquinoxaline-1(2H)-carboxylate (10, 0.23 g, 0.548 mmol) in dichloromethane (10 mL) was cooled to 0 °C, 4M HCl in dioxane (2 mL) was added slowly and the reaction mixture was stirred at room temperature for 16 h. The reaction mixture was basified using sodium bicarbonate solution and was extracted with dichloromethane. The combined organic layer washed with brine solution and dried over anhydrous sodium sulphate, concentrated solvent under reduced pressure to give crude residue which was purified by column chromatography over silica gel using 1% IPA in 8 % methanol/dichloromethane as a eluent to give the title compound (11) as a brown oily mass (0.04 g, crude).MS (ESI) *m/z* 220.1 (M+H) ^+^. The crude compound (after column purification) was proceeded to next step.

**Synthesis of 1-(5-imino-6-methyl-2,3,6,7-tetrahydro-1H,5H-pyrazino[3,2,1-ij]quinazolin-1-yl)ethan-1-one hydrobromide (Compound 15)-** To a solution of 1-(5-((methylamino)methyl)-3,4-dihydroquinoxalin-1(2H)-yl)ethan-1-one (**11**, 0.04 g, 0.728 mmol) in ethanol (3 mL) was added cyanogen bromide (0.023 g, 0.218 mmol). Then the reaction mixture was heated at 80 °C for 1 h. The reaction mixture was cooled to room temperature and concentrated solvent completely give crude residue which was purified using which was washed with acetonitrile, diethyl ether and n-pentane and dried completely to give the title **(compound 15)** as a brown solid (0.012 g, 20.33 % yield). ^1^H NMR (DMSO-*d_6_*_,_ 400 MHz) δ 2.19 (s, 3H), 3.13 (s, 3H), 3.79 (s, 2H), 3.91 (s, 2H), 4.53 (s, 2H), 6.95-7.21 (m, 2H), 7.46 (s, 1H), 8.13 (bs, 2H). MS (ESI) *m/z* 245.1(M+H)^+^-HBr. HPLC purity @ 264 nm, 98.14 %.

**Compound (16)**

**Synthesis of methyl 3-fluoro-2-nitrobenzoate (2)-** To a solution of 3-fluoro-2-nitrobenzoic acid (**1**, 2 g, 10.8 mmol) in 20 mL of methanol (20 mL) was cooled to 0 °C, sulfuric acid (3 mL) was added slowly and the reaction mixture was heated at 65 °C for 24 h. The reaction mixture was cooled to room temperature, concentrated solvent completely, basified using sodium bicarbonate solution and it was extracted with ethyl acetate. The combined organic layer was washed with brine solution and dried over anhydrous sodium sulphate, concentrated solvent under reduced pressure and dried completely to give the title compound **(2)** as an off-white solid (2.1 g, 97.6 %). ^1^H NMR (CDCl_3_, 400 MHz) δ 3.92 (s, 3H), 7.45 (t, *J* = 8.4 Hz, 1H), 7.56-7.60 (m, 1H), 7.80 (d, *J* = 7.6 Hz, 1H);

**Synthesis of methyl 3-(methylamino)-2-nitrobenzoate (2)-** To a solution of methyl 3-fluoro-2-nitrobenzoate (**2**, 2 g, 10 mmol) in tetrahydrofuran (15 mL) was added methylamine in water (2 mL) and the reaction mixture was stirred at room temperature for 1 h. The reaction mixture was diluted with water and it was extracted with ethyl acetate. The combined organic layer washed with brine solution and dried over anhydrous sodium sulphate, concentrated solvent under reduced pressure to give crude residue which was purified by combi flash purifier using 10% ethyl acetate/hexane as eluent to give the title compound **(3)** as a orange oily mass (1.3 g, 62 %). ^1^H NMR (CDCl_3,_ 400 MHz) δ 3.00 (d, *J* = 4.8 Hz, 3H), 3.88 (s, 3H), 6.76 (d, *J* = 6.8 Hz, 1H), 6.91 (d, *J* = 8.4 Hz, 1H), 7.43 (t, *J* = 8 Hz, 1H), 7.49 (bs, 1H); MS (ESI) *m/z* 211.1 (M+H) ^+^.

**Synthesis of methyl 2-amino-3-(methylamino)benzoate (3)-**To a solution of methyl 3-(methylamino)-2-nitrobenzoate (**3**, 1.1 g, 4.9 mmol) in ethanol (25 mL) was added stannous chloride (3.3 g, 14.71 mmol). The reaction mixture was stirred at 78 °C for 5 h. The reaction mixture was cooled to room temperature, concentrated solvent completely, basify using sodium bicarbonate solution and it was extracted with ethyl acetate. The combined organic layer washed with brine solution and dried over anhydrous sodium sulphate, concentrated solvent under reduced pressure and dried completely to give the title compound **(4)** as a yellow oily mass (0.91 g, crude).MS (ESI) *m/z* 181.1 (M+H) ^+^.

**Synthesis of 1-methyl-3-oxo-1,2,3,4-tetrahydroquinoxaline-5-carboxylic acid (4)-**To a solution of methyl 2-amino-3-(methylamino) benzoate (**4**, 0.91 g, 5.04 mmol) in water (10 mL) were added 2-chloroacetic acid (0.715 g, 7.5 mmol) and sodium hydroxide (0.403 g, 10 mmol). The reaction mixture was stirred at 100 °C for 16 h. The reaction mixture was cooled to room temperature, which was acidified using 1N HCl solution (P^H^-3 to 4), and it was extracted with ethyl acetate. The combined organic layer washed with brine solution and dried over anhydrous sodium sulphate, concentrated solvent under reduced pressure to give crude residue which was triturated with 10 % diethyl ether and hexane and dried completely to give the title compound **(5)** as a brown solid (0.61 g, crude).MS (ESI) *m/z* 207.0 (M+H) ^+^.

**Synthesis of N,1-dimethyl-3-oxo-1,2,3,4-tetrahydroquinoxaline-5-carboxamide (5)-** To a solution of 1-methyl-3-oxo-1,2,3,4-tetrahydroquinoxaline-5-carboxylic acid (**5**, 0.6 g, 2.9 mmol) in dichloromethane (10 mL) were added 2M methyl amine (2.18 mL, 4.36 mmol), N-(3-dimethylaminopropyl)-N′-ethylcarbodiimide hydrochloride (1.39 g, 7.27 mmol), triethyl amine (1.2 mL, 8.72 mmol) and hydroxybenzotriazole (0.2 g, 1.4 mmol). The reaction mixture was stirred at room temperature for 16 h. The reaction mixture was basifying using sodium bicarbonate solution and it was extracted with dichloromethane. The combined organic layer washed with brine solution and dried over anhydrous sodium sulphate, concentrated solvent under reduced pressure to give crude residue which was purified by combi flash purifier using 20% ethyl acetate/hexane as eluent to give the title compound **(6)** as brown a oily mass (0.333 g, 51.8 %).MS (ESI) *m/z* 220.1 (M+H) ^+^.

# Synthesis of N-methyl-1-(1-methyl-1,2,3,4-tetrahydroquinoxalin-5-yl)methanamine (6)-To a solution of N,1-dimethyl-3-oxo-1,2,3,4-tetrahydroquinoxaline-5-carboxamide (6, 0.33 g, 1.50 mmol) in tetrahydrofuran (15 mL) was cooled to 0 °C, then 1M LAH solution in THF (15.05 mL, 15.05 mmol) was added and the reaction mixture was heated at 65 °C for 48 h. The reaction mixture was cooled to room temperature and slowly quenched with ammonium chloride solution at 0 °C, add ethyl acetate and stirred for 0.5 h. The precipitate was filtered, the filtrate was extracted. The combined organic layer was washed with brine solution and dried over anhydrous sodium sulphate, concentrated solvent under reduced pressure to give crude residue which was purified by combi flash purifier using 8 % methanol/dichloromethane as eluent to give the title compound (7) as a yellow oily mass (0.195 g, 68 %). ^1^H NMR (CDCl_3,_ 400 MHz) δ 2.42 (s, 3H), 2.85 (s, 3H), 3.22 (t, *J* = 4.4 Hz, 2H), 3.52 (s, *J* = 4 Hz, 2H), 3.69 (s, 2H), 6.48-6.59 (m, 3H); MS (ESI) *m/z* 192.2 (M+H)^+.^

**Synthesis of 1,6-dimethyl-2,3,6,7-tetrahydro-1H,5H-pyrazino[3,2,1-ij]quinazolin-5-imine hydrobromide (Compound 16)-**To a solution of N-methyl-1-(1-methyl-1,2,3,4-tetrahydroquinoxalin-5-yl)methanamine (**7**, 0.19 g, 0.993 mmol) in ethanol (4 mL) was added cyanogen bromide (0.126 g, 1.19 mmol). Then reaction mixture was heated at 80° C for 1.5 h. The reaction mixture was cooled to room temperature and concentrated solvent completely give crude residue which was purified by combi flash purifier using 5% methanol/dichloromethane as eluent to give the title **(Compound 16)** as an off-white solid (0.04 g, 13.5 %). ^1^H NMR (DMSO-*d_6_*_,_ 400 MHz) δ 2.82 (s, 3H), 3.11 (s, 3H), 3.24 (t, *J* = 5.2 Hz, 2H), 3.86 (t, *J* = 4.8 Hz, 2H), 4.44 (s, 2H), 6.52 (d, *J* = 7.6 Hz, 1H), 6.67 (d, *J* = 8.4 Hz, 1H), 7.01 (t, *J* = 8.0 Hz, 1H), 7.97 (bs, 2H). ^13^C NMR (DMSO-d6) δ 152.14, 138.13, 125.54, 121.21, 119.93, 115.07, 112.30, 50.46, 48.00, 45.06, 38.41, 38.21. MS (ESI) *m/z* 217.2 (M+H)^+^-HBr. HPLC purity @ 240 nm, 97.49 %.

**Compound (17)**

**Compound 17** was synthesized following the procedure described for the synthesis of **compound 15** till the intermediate 9**.**

# Synthesis of tert-butyl 8-(((tert-butoxycarbonyl)(methyl)amino)methyl)-4-(dimethylcarbamoyl)-3,4-dihydroquinoxaline-1(2H)-carboxylate (10)- To a solution of tert-butyl 8-(((tert-butoxycarbonyl)(methyl)amino)methyl)-3,4-dihydro quinoxaline-1(2H)-carboxylate (intermediate-9 compound-17, 0.28 g, 0.74 mmol) in pyridine (6 mL) was added dimethylcarbamic chloride (0.119 g, 1.11 mmol) and the reaction mixture was stirred at 80 °C for 3 h. Diluted with water and was extracted with ethyl acetate. The combined organic layer washed with citric acid solution, brine solution and dried over anhydrous sodium sulphate, concentrated solvent under reduced pressure to give crude residue which was purified by combi flash purifier using 20% ethyl acetate/hexane as eluent to give the title compound (10) as a yellow solid (0.262 g, 79.15 % yield).MS (ESI) *m/z* 449.3 (M+H) ^+^.

# Synthesis of N,N-dimethyl-5-((methylamino)methyl)-3,4-dihydroquinoxaline-1(2H)-carboxamide (11)- To a solution of tert-butyl 8-(((tert-butoxycarbonyl)(methyl)amino)methyl)-4-(dimethylcarbamoyl)-3,4-dihydroquinoxaline-1(2H)-carboxylate (0.23 g, 0.513 mmol) in dichloromethane (10 mL) was cooled to 0 °C, trifluoroacetic acid (0.4 mL, 5.13 mmol) was added slowly and the reaction mixture was stirred at room temperature for 16 h. The reaction mixture was basified using sodium bicarbonate solution and was extracted with dichloromethane. The combined organic layer washed with brine solution and dried over anhydrous sodium sulphate, concentrated solvent under reduced pressure to give crude residue (11) which was used next step without any purification (0.135 g, crude). MS (ESI) *m/z* 249.2 (M+H) ^+^.

**Synthesis of 5-imino-N,N,6-trimethyl-2,3,6,7-tetrahydro-1H,5H-pyrazino[3,2,1-ij]quinazoline-1-carboxamide hydrobromide (Compound 17)-** To a solution of N,N-dimethyl-5-((methylamino)methyl)-3,4-dihydroquinoxaline-1(2H)-carboxamide (**11**, 0.135 g, 0.543 mmol) in ethanol (5 mL) was added cyanogen bromide (0.069 g, 0.652 mmol). Then the reaction mixture was heated at 80 °C for 1 h. The reaction mixture was cooled to room temperature and concentrated solvent completely give crude residue which was by combiflash purifier using 8 % methanol/dichloromethane as eluent to give **(Compound 17)** as a yellow solid (0.09 g, 47.12 % yield). ^1^H NMR (DMSO-*d_6_*_,_ 400 MHz) δ 2.82 (s, 6H), 3.12 (s, 3H), 3.60 (s, 2H), 3.84 (s, 2H), 4.49 (s, 2H), 6.79 (d, *J =* 8 Hz, 2H), 7.05 (t, *J =* 8 Hz, 1H), 8.07 (bs, 2H). ^13^C NMR (DMSO-d6) δ 157.85, 151.8, 130.33, 124.44, 122.63, 121.35, 120.61, 118.8, 49.96, 46.16, 42.24, 39.07, 37.92. MS (ESI) *m/z* 274.2(M+H) ^+^-HBr. HPLC purity @ 254 nm, 98.78 %.

**Compound (18)**

**Synthesis of 9-fluoro-6-methyl-2,3,6,7-tetrahydro-5H-[1,4]oxazino[2,3,4-ij]quinazolin-5-imine hydrobromide (Compound 18).** The compound was synthesized following the procedure described for the synthesis of **(Compound 19)** starting from 3-fluoro-6-nitro phenol. white solid (0.09 g, 53 %). ^1^H NMR (DMSO-d_6,_ 400 MHz) δ 3.1 (s, 3H), 3.9 (t, *J* = 4.4 Hz, 2H), 4.36 (t, *J* = 4.4 Hz, 2H), 4.53 (s, 2H), 6.73 (dd, *J* = 1.8 Hz, *J* = 8.4 Hz, 1H), 6.85 (dd, *J* = 2.4 Hz, *J* = 10 Hz, 1H), 8.11 (bs, 2H); MS (ESI) *m/z* 222.1 (M+H)+-HBr. HPLC purity 99.27 %.

**Compound (19)**

**Synthesis of 6-amino-2,3-difluorophenol (2)-**To a suspension of 2,3-difluoro-6-nitrophenol (**1**, 10 g, 57.11 mmol) in ethanol (150 mL) was added 10 % Pd/C (2 g) under nitrogen atmosphere. The reaction mixture was stirred at room temperature for 48 h under hydrogen balloon. After completion of the reaction it was filtered through diatomaceous earth and washed with ethanol, the filtrate was concentrated under reduced pressure to get desired crude compound. The obtained crude was washed with pentane and concentrated to afford the title compound **(2)** as a brown solid (6.0 g, 72.3 %). MS (ESI) *m/z* 146.1 (M+H) ^+^.

**Synthesis 7,8-difluoro-3,4-dihydro-2H-benzo[b][1,4]oxazine (3)-** To a solution of 6-amino-2,3-difluorophenol (**2**, 5.0 g, 39.2 mmol) in dimethylformamide (50 mL) were added 1,2 dibromoethane (4.08 mL, 47.1 mmol) and potassium carbonate (16.28 g, 112.8 mmol) and the reaction mixture was heated at 125 °C for 16 h. The reaction mixture was cooled to room temperature, diluted with ice water, the precipitate formed was filtered. The filtrate was extracted with ethyl acetate. The combined organic layer washed with cold water (100 mL*2), brine solution and dried over anhydrous sodium sulphate, concentrated solvent under reduced pressure to give the title compound **(3)** as a black color solid (2.7 g, 40.3 %). ^1^H NMR (CDCl_3,_ 400 MHz) δ 3.41 (t, *J* = 4 Hz, 2H), 3.69 (s, 1H), 4.30 (t, *J* = 4.4 Hz, 2H), 6.24-6.27 (m, 1H), 6.54 (q, *J* = 9.6 Hz, 1H); MS (ESI) *m/z* 172.1 (M+H) ^+^.

**Synthesis of 7,8-difluoro-3,4-dihydro-2H-benzo[b][1,4]oxazine hydrochloride (4)-**To a solution of 7,8-difluoro-3,4-dihydro-2H-benzo[b][1,4]oxazine (**3**, 0.3 g, 1.75 mmol) in methanol (5 mL) was cooled to 0 °C, 4 M HCl in dioxane (0.5 mL) was added and the reaction mixture was stirred at room temperature for 1 h. The reaction mixture was concentrated completely under reduced pressure to give the crude compound **(4)** as a brown solid (0.34 g, 93.6 %). The crude proceeds to next step without further purification. ^1^H NMR (DMSO-d_6,_ 400 MHz) δ 3.27 (t, *J* = 4 Hz, 2H), 4.19 (t, *J* = 4.4 Hz, 2H), 6.36-6.40 (m, 1H), 6.68 (q, *J* = 8.8 Hz, 1H), 6.94 (bs, 2H).

**Synthesis of (E)-2-(7,8-difluoro-2,3-dihydro-4H-benzo[b][1,4]oxazin-4-yl)-2-oxoacetalde hyde oxime (5)-** To a stirred solution of chloral hydrate (**4**, 0.325 g, 1.96 mmol) in 5 mL of water was added with sodium sulphate (2.3 g, 16.3 mmol) and 7,8-difluoro-3,4-dihydro-2H-benzo[b][1,4]oxazine hydrochloride (0.34 g, 1.63 mmol) in water (1.0 mL) followed by hydroxylamine hydrochloride (0.341 g, 4.91 mmol) in water and the reaction mixture was heated at 80 °C for 5 h. The reaction mixture was cooled to room temperature, basify using sodium bicarbonate solution and was extracted with ethyl acetate. The combined organic layer washed with brine solution, dried over anhydrous sodium sulphate, concentrated solvent under reduced pressure to give crude residue which was purified by combi flash purifier using 100 % hexane as eluent to give the title compound **(5)** as an off brownish solid (0.38 g, 96 %). ^1^H NMR (DMSO-d_6,_ 400 MHz) δ 3.98 (t, *J* = 4 Hz, 2H), 4.39 (t, *J* = 4.4 Hz, 2H), 6.95 (q, *J* = 9.6 Hz, 1H), 7.38 (bs, 1H), 7.84 (s, 1H); MS (ESI) *m/z* 243.1 (M+H) ^+^.

**Synthesis of 8,9-difluoro-2,3-dihydro-[1,4]oxazino[2,3,4-hi]indole-5,6-dione (Compound 6)-**

To a stirred solution of sulfuric acid (1.6 mL) in water (0.65 mL) was added (E)-2-(7,8-difluoro-2,3-dihydro-4H-benzo[b][1,4]oxazin-4-yl)-2-oxoacetaldehyde oxime (**5**, 0.38 g 1.56 mmol) portion wise over 15 min at 60 °C. After the addition was complete, the reaction mixture was heated to 80 °C for 30 min. The reaction mixture was cooled to room temperature, diluted with water and was extracted with ethyl acetate (100 mL*2). The combined organic layer washed with brine solution and dried over anhydrous sodium sulphate, concentrated solvent under reduced pressure to give crude residue which was purified by combi flash purifier using 15 % ethyl acetate/hexane mixture as eluent to give the title compound **(6)** as a red solid (0.2 g, 56.6 %). ^1^H NMR (DMSO-d_6,_ 400 MHz) δ 3.81 (t, *J* = 4.8 Hz, 2H), 4.43 (t, *J* = 4.8 Hz, 2H), 7.38 (t, *J* = 8.4 Hz, 1H); MS (ESI) *m/z* 225.1 (M-H) ^+^.

**Synthesis of 7,8-difluoro-3,4-dihydro-2H-benzo[b][1,4]oxazine-5-carboxylic acid (7)-** To a solution of 8,9-difluoro-2,3-dihydro-[1,4]oxazino[2,3,4-hi]indole-5,6-dione (**6**, 0.2 g, 0.88 mmol) in 4.5 % sodium hydroxide solution (1 mL) was added 30 % hydrogen peroxide drop wise over 15 mins and stirred at room temperature for 15 min. After completion of the reaction, the reaction mixture was acidified using citric acid solution (p^H^-3 to 4), the solid precipitate was filtered, the residue was washed with water and dried to give the title compound as pure compound **(7)** as an off yellow solid (0.17 g, 89 %). ^1^H NMR (DMSO-d_6,_ 400 MHz) δ 3.45 (t, *J* = 4 Hz, 2H), 4.20 (t, *J* = 4.4 Hz, 2H), 7.25-7.20 (m, 1H), 7.59 (bs, 1H), 12.92 (bs, 1H), 13.02 (bs, 1H). MS (ESI) *m/z* 216.0 (M+H) ^+^.

**Intermediate 7 was converted to the compound 19, Following procedure described for compound 2 -**^1^H NMR (DMSO-d_6,_ 400 MHz) δ 3.10 (s, 3H), 3.93 (t, *J* = 4.8 Hz, 2H), 4.44 (t, *J* = 4.4 Hz, 2H), 4.50 (s, 2H), 6.95 (t, *J* = 10 Hz, 2H), 8.20 (bs, 2H). 13C NMR (DMSO-d_6_) δ 158.63, 151.06, 145.11, 121.8, 117.6, 104.96, 103.53, 60.05, 48.9, 42.68, 37.84.MS (ESI) *m/z* 240.1 (M+H) ^+.^ HPLC purity @ 260 nm 99.73 %.

**Compound (20)**

**Synthesis of 3, 5-difluoro-2-nitrobenzoic acid (2)-** To a solution of 3, 5-difluorobenzoic acid (**1**, 5.0 g, 31.64 mmol) in conc.H_2_SO_4_ (15.0 mL) and HNO_3_ (7.0 mL) was added at 0 °C and stirred for 12h at room temperature. The reaction mixture was cooled to room temperature and quenched with cold water (10.0 mL) and extracted into ethyl acetate. The organic layer was washed with water and brine, dried over sodium sulphate, filtered and concentrated under reduced pressure to afford the desired product **(2)** as a yellow solid (6.0 g, 93 %). ^1^H NMR (DMSO-*d*_6,_ 400 MHz) δ 7.68 (d, *J* = 8.0 Hz, 1H), 7.98 (t, *J* = 8.0 Hz, 1H), 14.20 (bs, 1H); MS (ESI) *m/z* 202.1 (M-H)^+^.

**Synthesis of Methyl 3, 5-difluoro-2-nitrobenzoate (3)-** To a solution of 3, 5-difluorobenzoic acid (**2**, 6.0 g, 29.55 mmol) in *N*, *N*-Dimethyl formamide (30.0 mL) was added sodium carbonate (9.4 g, 88.65 mmol) and methyl iodide (8.4 g, 59.11 mmol) at 0 °C and stirred for 4 h at 80 °C. After completion of the reaction [monitored by TLC, ethyl acetate: hexane (3:7)]. The reaction mixture was quenched with cold water (50.0 mL) and extracted into ethyl acetate. The organic layer was washed with water and brine, dried over sodium sulphate, filtered and concentrated under reduced pressure to afford the desired product **(3)** as a brown yellow viscous liquid (6.0 g, 93 %).^1^H NMR (DMSO-*d*_6,_ 400 MHz) δ 3.93 (s, 3H), 7.17 (t, *J* = 6.0 Hz, 1H), 7.51 (d, *J* = 8.0 Hz, 1H).

**Synthesis of Methyl 5-fluoro-3-(methylamino)-2-nitrobenzoate (4)-** Aqueous methylamine solution (2.5 mL) was added drop wise to methyl 3, 5-difluoro-2-nitrobenzoate (**3,** 6.0g, 27.64 mmol) at 0 °C, over 15 mins. After completion of the addition the reaction mixture was stirred at 0 °C for 15 mins, to the mixture water was added and the resulting yellow crystal was collected by filtration. Finally the crystal was washed with pentane and ether to afford the desired product **(4)**; MS (ESI) *m/z*2 28.1 (M+H)^+^.

**Synthesis of Methyl 2-amino-5-fluoro-3-(methylamino) benzoate (5) -**To a solution of methyl 5-fluoro-3-(methyl amino)-2-nitrobenzoate (**4,** 4.5 g, 19.73 mmol) in ethyl acetate (30.0 mL) was added Pd/C (1.0 g), then the mixture was stirred 12 h under H_2_ atmosphere at room temperature. After completion of the reaction [monitored by TLC, ethyl acetate: hexane (3:7)]. The reaction mixture was filtered through diatomaceous earth; Organic layer concentrated under reduced pressure. The obtained crude was purified by combi-flash purifier with 10% ethyl acetate in hexane as eluent to afford the desired product **(5)** as a yellow solid (2.75 g, 70%); MS (ESI) *m/z* 183.1 (M+H)^+^.

**Synthesis of Methyl 7-fluoro-1-methyl-3-oxo-1, 2, 3, 4-tetrahydroquinoxaline-5-carboxylate (6) -**To a stirred solution of methyl 2-amino-5-fluoro-3-(methylamino) benzoate (**5,** 2.0 g, 10.05 mmol) in water (15.0 mL) was added NaOH (0.804 g, 20.10 mmol) and 2-chloroacetic acid (1.43 g, 15.07 mmol), then the reaction mixture was heated to 100 °C until the completion of the reaction [ monitored by TLC, ethyl acetate: hexane (4:6)]. The reaction mixture adjusts P^H^ into 4 by using aqueous HCl solution and extracted into ethyl acetate. The organic layer was washed with water and brine, dried over sodium sulphate, filtered and concentrated under reduced pressure. The obtained crude was purified by combi-flash purifier with 15% ethyl acetate in hexane as eluent to afford the desired product **(6)** as a yellow solid (0.6 g). ^1^H NMR (DMSO-*d*_6,_ 400 MHz) δ 2.82 (s, 3H), 3.84 (s, 2H), 3.85 (s, 3H), 6.84(dd, *J* = 2.0 Hz, *J* = 10.0 Hz,1H), 6.9 8(dd, *J* = 2.0 Hz, *J* = 10.0 Hz, 1H), 10.27 (s, 1H); MS (ESI) *m/z* 239.0 (M+H)^+^.

**Synthesis of 7-fluoro-1-methyl-3-oxo-1, 2, 3, 4-tetrahydroquinoxaline-5-carboxylic acid (7)-** To a stirred solution of methyl 7-fluoro-1-methyl-3-oxo-1, 2, 3, 4-tetrahydroquinoxaline-5-carboxylate (**6,** 0.6 g, 2.5 mmol) in methanol (5.0 mL) was added 5N aqueous NaOH solution (1.5 mL), then the reaction mixture was heated to 65 °C until the completion of the reaction [ monitored by TLC, ethyl acetate: hexane (5:5)]. The reaction mixture was concentrated under reduced pressure to remove all the methanol portions completely then washed with diethyl ether to remove the non-polar impurities, then the viscous portion was diluted with minimum amount of water; adjust P^H^ into 4 by using aqueous HCl solution and extracted into ethyl acetate. Organic layer was washed with water and brine, dried over sodium sulphate, filtered and concentrated under reduced pressure affords the desired product **(7)** as a pale yellow solid (0.5 g, 88 %). ^1^H NMR (DMSO-*d*_6,_ 400 MHz) δ 2.81 (s, 3H), 3.83 (s, 2H), 6.81 (dd, *J* = 2.0 Hz, *J* = 10.0 Hz, 1H), 6.98 (dd, *J* = 2.0 Hz, *J* = 10.0 Hz, 1H), 13.85 (bs, 1H); MS (ESI) *m/z* 225.1 (M+H)^+^.

**Synthesis of 7-fluoro-N, 1-dimethyl-3-oxo-1, 2, 3, 4-tetrahydroquinoxaline-5-carboxamide (8)**- To a solution of 7-fluoro-1-methyl-3-oxo-1, 2, 3, 4-tetrahydroquinoxaline-5-carboxylic acid (**7,** 0.5 g, 2.23 mmol) in dichloromethane (10.0 mL) was added triethylamine (0.68 mL, 6.69 mmol) at 0 °C followed by 2M methylamine solution in THF (2.0 mL, 3.34 mmol) and 1-propane phosphoric acid cyclic anhydride (2.0 mL, 6.69 mmol) and stirred for 12h at room temperature. The reaction mixture was concentrated under reduced pressure, and added water (10 mL), then extracted in to EtOAc, Organic layer was washed with saturated NaHCO_3_ solution and brine, dried over sodium sulphate, filtered and concentrated to afford the desired product **(8)** as a colorless oil (0.35 g, 67 %). ^1^H NMR (DMSO-*d*_6,_ 400 MHz) δ 2.73 (d, *J* = 8.0 Hz, 2H), 2.79 (s, 3H), 3.77 (s, 3H), 6.72 (d, *J* = 10.0 Hz, 1H), 6.95 (dd, *J* = 2.0 Hz, *J* = 10.0 Hz, 1H), 8.65 (s, 1H), 11.16 (s, 1H); MS (ESI) *m/z* 238.1 (M+H)^+^.

**Synthesis of 1-(7-fluoro-1-methyl-1, 2, 3, 4-tetrahydroquinoxalin-5-yl)-N-methyl methanamine (9)-**To a solution of 7-fluoro-N, 1-dimethyl-3-oxo-1, 2, 3, 4-tetrahydroquinoxaline-5-carboxamide (**8,** 0.35, 1.47 mmol g) in tetrahydrofuran (30.0 mL), was added BH_3_.DMS (10.0 mL) at 0 °C and stirred for 48h at 65 °C. The reaction mixture was quenched with methanol at 0 °C and concentrated under reduced pressure to afford the desired product and then 5.0 mL 4M HCl in Dioxan was added to the mixture then stirred for 2h at room temperature. After 2h reaction mixture was concentrated under reduced pressure, then mixture was basified with saturated NaHCO_3_ solution and extracted into ethyl acetate. Organic layer was washed with water and brine, dried over sodium sulphate, filtered and concentrated under reduced pressure. The obtained crude was purified by combi-flash purifier with 8% methanol in dichloromethane as eluent to afford the desired product **(9)** as an off-white solid (0.15 g, 50 %). ^1^H NMR (DMSO-*d*_6,_ 400 MHz) δ 2.27 (s, 3H), 2.75 (s, 3H), 3.12 (t, *J* = 4.8 Hz, 2H), 3.31 (t, *J* = 4.8 Hz, 2H), 3.52 (s, 2H), 5.10 (bs, 2H), 6.62-6.25 (m, 2H); MS (ESI) *m/z* 210.2 (M+H)^+^.

**Synthesis of 9-fluoro-1,6-dimethyl-2,3,6,7-tetrahydro-1H,5H-pyrazino[3,2,1-ij]quinazolin-5-imine, 2,2,2-trifluoroacetate salt hydrobromide (Compound 20)-**To a solution of 1-(7-fluoro-1-methyl-1, 2, 3, 4-tetrahydroquinoxalin-5-yl)-N-methylmethanamine (**9**, 0.15 g, 0.717 mmol) in ethanol (5.0 mL), then cyanogen bromide (0.12 g, 1.07 mmol) was added, and the reaction mixture was stirred at 85 °C for 2 h, after cooled to room temperature reaction mixture was concentrated under reduced pressure. The obtained crude was purified by preparative HPLC by using 0.1% TFA in Water/ CH_3_CNas eluent to afford the desired product **(Compound 20)** as a viscous solid (0.03 g, 18 %). ^1^H NMR (DMSO-*d*_6,_ 400 MHz) δ 2.84 (s, 3H), 3.09 (s, 3H), 3.31 (t, *J* = 4.4 Hz, 2H), 3.86 (t, *J* = 4.8 Hz, 2H), 4.18 (bs, 3H), 4.41 (s, 2H), 6.36-6.38 (m, 1H), 6.95 (dd, *J* = 2.0 Hz, *J* = 10.0 Hz, 1H), 8.01 (bs, 2H); MS (ESI) *m/z* 235.1 (M+H)^+^-HBr;HPLC Purity @ 254nm, 99.50%.

**Compound (21)**

**Synthesis of 7-fluoro-N-(2,2,2-trifluoroethyl)-3,4-dihydro-2H-benzo[b][1,4]oxazine-5-carboxamide (2)-** To a solution of 7-fluoro-3,4-dihydro-2H-benzo[b][1,4]oxazine-5-carboxylic acid (**1**, 0.3 g, 1.52 mmol) in dichloromethane (10 mL) were added 2,2,2-trifluoroethan-1-amine (0.226 g, 2.28 mmol), N-(3-dimethylaminopropyl)-N′-ethylcarbodiimide hydrochloride (0.436 g, 2.28 mmol), n-methylmorpholine (0.475 g, 4.56 mmol) and 1-hydroxy-7-azabenzotriazole. The reaction mixture was stirred at room temperature for 16 h. The reaction mixture was basifying using sodium bicarbonate solution and was extracted with dichloromethane. The combined organic layer washed with brine solution and dried over anhydrous sodium sulphate, concentrated solvent under reduced pressure to give crude residue which was purified by Combi flash purifier using 20% ethyl acetate/hexane as eluent to give the title compound **(2)** as a gummy solid (0.188 g, 42.5 %). MS (ESI) *m/z* 279.1 (M+H)^+.^

**Synthesis of 2,2,2-trifluoro-N-((7-fluoro-3,4-dihydro-2H-benzo[b][1,4]oxazin-5-yl)methyl) ethan-1-amine (3)-** To a solution of 7-fluoro-N-(2,2,2-trifluoroethyl)-3,4-dihydro-2H-benzo[b][1,4]oxazine-5-carboxamide (**2**, 0.18 g, 0.647 mmol) in tetrahydrofuran (5 mL) was cooled to 0 °C, Borane dimethyl sulfide complex (0.61 mL, 6.47 mmol) was added. After the addition complete, the reaction mixture was stirred at 65 °C for 48 h. The reaction mixture cooled to room temperature and slowly quenched with methanol at 0 °C, concentrate completely solvent, 4 M HCl in dioxane was added and stirred for 1 h. Again concentrate solvent, basifying using sodium bicarbonate solution and it was extracted with ethyl acetate. The combined organic layer washed with brine solution and dried over anhydrous sodium sulphate, concentrated solvent under reduced pressure to give crude residue which was purified by Combi flash purifier using 1% ethyl acetate/hexane as eluent to give the title compound **(3)** as an oily mass (0.09 g, 53 %). ^1^H NMR (DMSO-*d_6,_* 400 MHz) δ 3.17-3.22 (m, 2H), 3.27 (s, 2H), 3.65 (s, 2H), 4.07 (t, *J* = 3.6 Hz, 2H), 5.25 (bs, 1H), 6.46 (dd, *J* = 2.0 Hz, *J* = 9.6 Hz, 1H), 6.54-6.56 (m, 1H); MS (ESI) *m/z* 265.1 (M+H) ^+^.

**Synthesis of 9-fluoro-6-(2,2,2-trifluoroethyl)-2,3,6,7-tetrahydro-5H-[1,4]oxazino[2,3,4-ij]quinazolin-5-imine hydrobromide (Compound 21)-** To a solution of 2,2,2-trifluoro-N-((7-fluoro-3,4-dihydro-2H-benzo[b][1,4]oxazin-5-yl)methyl)ethan-1-amine (**3**, 0.09 g, 0.34 mmol) in ethanol (3 mL) was added cyanogen bromide (0.043 g, 0.4 mmol).Then reaction mixture was heated at 80° C for 1 h. The reaction mixture was cooled to room temperature and concentrated solvent completely give crude residue which was triturated using 5 % diethyl ether and n-pentane and dried to give the title **(Compound 21)** as a white solid (0.048 g, 38 %). ^1^H NMR (DMSO-*d_6_*_,_ 400 MHz) δ 3.95 (s, 2H), 4.37 (s, 2H), 4.52-4.59 (m, 2H), 4.63 (s, 2H), 6.80 (d, *J* = 8.4 Hz, 1H), 6.87 (d, *J* = 9.6 Hz, 1H), 8.54 (bs, 2H). MS (ESI) *m/z* 290.1 (M+H) ^+^-HBr. 13C NMR (DMSO-d6) δ 159.06, 151.96, 145.45, 126.1, 122.2,117.54, 104.96, 103.8, 64.19, 49.95, 49.5, 48.77, 43.35. HPLC purity @ 262 nm, 99.04 %.

**Compound (22)**

**Synthesis of (E/Z)-3-(2-fluorophenyl)acrylonitrile (2)** To a suspension of diethyl (cyanomethyl)phosphonate (5.23 mL, 32.2 mmol) in THF (30 mL) was added sodium hydride (1.28 g, 32.2 mmol) portion wise over 10 minutes at 0 °C and the reaction mixture was stirred 1 hour at 0 °C. Then added the solution of 2-fluorobenzaldehyde (1, 4.0 g, 32.2 mmol) at 0 °C and the reaction mixture was stirred for 2 h at room temperature. Reaction mixture was quenched with crushed ice, extracted with ethyl acetate and separated the organic layer. Organic layer was washed with water and brine, dried over sodium sulphate, filtered and concentrated under reduced pressure to get desired crude compound. The obtained crude was purified by combi-flash purifier with 5% ethyl acetate in hexane as eluent to afford the desired product (**2**) as a colorless liquid (4.6 g, 95 %). ^1^H NMR (DMSO-*d*_6,_ 400 MHz) δ 6.48 (d, *J* = 16.8 Hz, 1H), 7.25-7.31 (m, 2H), 7.47-7.53 (m, 1H), 7.65 (d, *J* = 16.8 Hz, 1H), 7.73-7.78 (m, 1H);

**Synthesis of 3-(2-fluorophenyl)propan-1-amine (3)** To a solution of (E/Z)-3-(2-fluorophenyl)acrylonitrile (2, 4.8 g, 32.61 mmol) in ethanol (50 mL) was added NH_3_ solution (20 mL) followed by added Raney Ni (4.0 g) and the reaction mixture was stirred for 12 h under H_2_ atmosphere (20 psi). After completion of the reaction [monitored by TLC, Methanol: DCM (0.5:9)]. The reaction mixture was filtered through diatomaceous earth, organic layer concentrated under reduced pressure to afford the desired product (**3**) as a brown liquid (5 g). ^1^H NMR (DMSO-*d*_6,_ 400 MHz) δ 1.23-1.27 (m, 1H), 1.55-1.62 (m, 2H), 2.50-2.53 (m, 2H, proton underneath with DMSO-*d*6 peak), 2.57-2.61 (m, 2H), 7.07-7.11 (m, 2H), 7.18-7.21 (m, 1H), 7.24-7.28 (m, 1H); MS (ESI) *m/z* 154.2 (M+H)^+^.

**Synthesis of 3-oxo-3,4-dihydro-2H-benzo[b][1,4]oxazine-5-carboxylic acid (4)** Methyl 3-oxo-3,4-dihydro-2H-benzo[b][1,4]oxazine-5-carboxylate was synthesized according to procedure described for compound 12 for **(12)**. To a solution of methyl 3-oxo-3,4-dihydro-2H-benzo[b][1,4]oxazine-5-carboxylate (0.785 g, 3.70 mmol) in methanol (10 mL) was added 5N NaOH aqueous solution (1.5 mL, 7.5 mmol) then the reaction mixture was stirred at 55 °C for 12 h. After completion of the reaction [monitored by TLC, ethyl acetate: hexane (4:6)], the reaction mixture was concentrated under reduced pressure to remove methanol and the residue obtained was acidified with 1M HCl aqueous solution to pH~3. The solid obtained was filtered through diatomaceous earth, dried, under vacuum pressure to afford the desired product (**4**) as a pale brown solid (0.66 g, 91 %). ^1^H NMR (DMSO-*d*_6,_ 400 MHz) δ 4.68 (s, 2H), 7.01(t, *J* = 8 Hz 1H), 7.22 (d, *J* = 8 Hz, 1H) 7.56 (d, *J* = 8 Hz, 1H), 10.44 (s, 1H), 13.78 (s, 1H); MS (ESI) *m/z* 194.1 (M+H)^+^.

**Synthesis of N-(3-(2-fluorophenyl)propyl)-3-oxo-3,4-dihydro-2H-benzo[b][1,4]oxazine-5-carboxamide (5)** To a solution of 3-oxo-3,4-dihydro-2H-benzo[b][1,4]oxazine-5-carboxylic acid (**4**, 0.5 g, 2.60 mmol) in DMF (10 mL) was added triethylamine (1.1 mL, 7.8 mmol) at 0 °C followed by solution of 3-(2-fluorophenyl)propan-1-amine (0.79g, 5.2 mmol) in DMF and EDC.HCl (0.74 g, 1.56 mmol), HOBt (0.19g, 1.3mmol) and stirred for 16 h at room temperature. To the reaction mixture was added water (10 mL), then extracted with ethyl acetate. Organic layer was washed with saturated NaHCO_3_ solution and brine, dried over sodium sulphate, filtered and concentrated under reduced pressure. The obtained crude was purified by combi-flash purifier with 30 % ethyl acetate-hexane as eluent to afford the desired product (**5)** as a pale yellow solid (0.17 g, 19 %); MS (ESI) *m/z* 329.1 (M+H)^+^.

**Synthesis of N-((3,4-dihydro-2H-benzo[b][1,4]oxazin-5-yl)methyl)-3-(2-fluorophenyl)propan-1-amine (6)** To a solution of N-(3-(2-fluorophenyl)propyl)-3-oxo-3,4-dihydro-2H-benzo[b][1,4]oxazine-5-carboxamide (5, 0.12 g, 0.36 mmol) in THF (10 mL) was added 2M LiAlH_4_ solution in THF (1.82 mL, 3.60 mmol) at 0 °C and the reaction mixture was stirred at 65 °C for 48 h. After refluxing, the reaction mixture was quenched with wet Na_2_SO_4_ at 0 °C and diluted ethyl acetate then it was filtered through diatomaceous earth and separated the organic layer. Organic layer was washed with water and brine, dried over sodium sulphate, filtered and concentrated under reduced pressure to get desired crude compound. The obtained crude was purified by combi-flash purifier with 10 % methanol in dichloromethane as eluent to afford the desired product (6) as a brown viscous oil (0.02 g, 18 %). ^1^H NMR (DMSO-*d*_6,_ 400 MHz) δ 1.65-1.75 (m, 2H), 2.50 (m, 2H, proton underneath with DMSO-*d*6 peak) 2.55-2.65 (m, 2H), 3.0-3.31 (m, 2H), 3.58 (s, 2H), 4.06 (t,  *J* = 4 Hz, 2H), 5.84 (bs, 1H), 6.41 (t, *J* = 8 Hz, 1H), 6.55 (d, *J* = 8 Hz, 1H), 6.59 (d, *J* = 7.2 Hz, 1H), 7.06-7.18(m, 3H), 7.21-7.25 (m, 2H); MS (ESI) *m/z* 301.2 (M+H)^+^.

**Synthesis of 6-(3-(2-fluorophenyl)propyl)-2,3,6,7-tetrahydro-5H-[1,4]oxazino[2,3,4-ij]quinazolin-5-imine hydrobromide (Compound 22)** To a solution of N-((3,4-dihydro-2H-benzo[b][1,4]oxazin-5-yl)methyl)-3-(2-fluorophenyl) propan-1-amine (6, 0.02 g, 0.066 mmol) in ethanol (5 mL) was added cyanogen bromide (0.084 g, 0.079 mmol) and the reaction mixture was stirred at 85 °C for 4 h. After cooled to room temperature, reaction mixture was concentrated under reduced pressure. Crude obtained was purified by combi-flash purifier with 10% methanol-DCM as eluent to afford the desired product (Compound **22**) as a brown semisolid (0.008 g, 38 %). ^1^H NMR (DMSO-*d*_6,_ 400 MHz) δ 1.85-2.0 (m, 2H), 2.59-2.67 (m, 2H), 3.49-3.59 (m, 2H), 3.85-3.95 (m, 2H), 4.25-4.35 (m, 2H), 4.54 (s, 2H), 6.79 (d, *J* = 6.8 Hz, 1H), 6.88 (d, *J* = 8 Hz, 1H), 7.04 (t, *J* = 8 Hz, 1H), 7.08-7.31 (m, 5H); MS (ESI) *m/z* 326.1 (M+H)^+^; HPLC Purity @ 290 nm, 94.07 %.

**Compound (23)**

**Synthesis of 1-(1H-indol-7-yl)-N-methylmethanamine (2)-**To a solution of 1H-indole-7-carbaldehyde (**1**, 1 g, 6.89 mmol) in MeOH (20 mL) was added methylamine (40% in water), (1.14 mL, 10.34 mmol) at 0 °C, and stirred for 90 mins at room temperature. Then sodium borohydride (0.39 g, 10.34 mmol) was added by lot’s wise over 15 mins at 0°C. The reaction mixture was stirred for 1 h at rt. The reaction mixture was concentrated under reduced pressure, and added water (10 mL), then extracted in to EtOAc, Organic layer was washed with water and brine, dried over sodium sulphate, filtered and concentrated under reduced pressure to afford the desired product **(2)** as a light green semi solid (1 g crude). MS (ESI) *m/z* 161.1 (M+H)+.

**Synthesis of 2-methyl-1,2,5,6-tetrahydro-3H-pyrrolo[3,2,1-ij]quinazolin-3-imine-(Compound 23)-**To a solution of 1-(1H-indol-7-yl)-N-methylmethanamine (**2**, 0.5 g, 3.12 mmol) in ethanol (20 mL) was added cyanogen bromide (0.364 g, 3.43 mmol). Then the reaction mixture was heated at 85 °C for 16 h. The reaction mixture was cooled to room temperature and concentrated under reduced pressure. The crude was purified by prep HPLC using Inertsil ODS 3V(250mm X 4.6mm X 5mic) with 0.1% TFA in water and ACN as a mobile phases, the compound was dried in a lyophilizer to give the title **(Compound 23)** as a white solid (0.025 g, 4.3 %). ^1^H NMR (DMSO-d_6,_ 400 MHz) δ 2.83 (s, 3H), 4.41 (s, 2H), 6.46 (s, 1H), 7.01 (t, *J* =7.6 Hz, 1H), 7.07 (d, *J* =7.2 Hz, 1H), 7.38 (t, *J* =2.8 Hz, 1H), 7.54 (d, *J* = 8.0 Hz, 1H), 11.15 (bs, 1H). HPLC purity 99.27 %. MS (ESI) m/z 186.1 (M+H)+.

**Compound-(24)**

**Synthesis of Ethyl 8-bromo-1-naphthoate (2)-**To a solution of 8-bromo-1-naphthoic acid (**1**, 1.0 g, 3.98 mmol) in N, N-dimethyl formamide (10.0 mL) was added potassium carbonate (1.37 g, 9.96 mmol) and ethyl iodide 0.8 mL, 9.96 mmol) and stirred for 12 h at room temperature. The reaction mixture was quenched with ice, and then extracted into ethyl acetate, Organic layer was washed with water, saturated NaHCO_3_ solution and brine, dried over sodium sulphate, filtered and concentrated under reduced pressure to afford the desired product **(2)** as a yellow viscous oil (1.0 g, 90 % yield). ^1^H NMR (DMSO-*d*_6,_ 400 MHz) δ 1.30 (t, *J* = 7.2 Hz, 3H), 4.35(q, *J* = 7.2 Hz, 2H), 7.48 (t, *J* = 8.0 Hz, 1H), 7.61 (t, *J* = 8.0 Hz, 1H), 7.67(d, *J* = 6.4 Hz, 1H), 7.95 (d, *J* = 8.0 Hz, 1H), 8.08 (d, *J* = 7.6 Hz, 1H), 8.13 (d, *J* = 8.0 Hz, 1H); MS (ESI) *m/z* 281.0 (M+2H)^+^.

**Synthesis of Ethyl 8-methyl-1-naphthoate (3)-** To a solution of ethyl 8-bromo-1-naphthoate (**2**, 1.3 g, 4.65 mmol) in dioxane (10.0 mL) was added potassium carbonate (0.96 g, 6.97 mmol) and 2,4,6-trimethyl-1,3,5,2,4,6-trioxatriborinane (0.7 mL, 5.59 mmol) and purged with nitrogen for 10 mins, followed by added Tetrakis(triphenylphosphine)palladium(0) (0.53 g, 0.46 mmol), again purged with nitrogen for 10 mins and refluxed for 12 h. After refluxing overnight, the reaction mixture was cooled to room temperature and filtered through diatomaceous earth and filtrate is concentrated under reduced pressure to get crude. The obtained crude was purified on silica column with 4% ethyl acetate in hexane as eluent to afford the desired product **(3)** as a viscous oil (0.51 g, 51 % yield); MS (ESI) *m/z* 215.1 (M+H)^+^.

**Synthesis of 8-methyl-1-naphthoic acid (4)-**To a stirred solution of ethyl 8-methyl-1-naphthoate (**3**, 0.5 g, 2.33 mmol) in ethanol (10.0 mL) was added 5 N NaOH aqueous solution (3.5 mL) and stirred for 2 h at 70 °C. The reaction mixture was concentrated under reduced pressure and diluted with water and adjusts P^H^ into 4 by using aqueous HCl solution and extracted into ethyl acetate. The organic layer was washed with water and brine, dried over sodium sulphate, filtered and concentrated under reduced pressure to afford the desired product **(4)** as a yellow solid (0.4 g, 92% yield). ^1^H NMR (DMSO-*d*_6,_ 400 MHz) δ 2.63 (s, 3H), 7.40-7.64 (m, 4H), 7.83 (d, *J* = 8.0 Hz, 1H), 7.99 (d, *J* = 8.0 Hz, 1H), 13.10 (bs, 1H); MS (ESI) *m/z* 185.1 (M-H)^+^.

**Synthesis of 8-methyl-1-naphthamide (5)-** To a solution of 8-methyl-1-naphthoic acid (**4**, 0.4 g, 2.70 mmol) in dichloromethane (10.0 mL) was added oxalyl chloride (0.3 mL, 3.24 mmol) and dimethyl formamide (0.1 mL, catalytic) at 0 °C and stirred for 4 h at 0 °C, and concentrated under nitrogen atmosphere then it was dissolved into dichloromethane (10 mL) and purged with ammonia gas for 10 mins at 0 °C, and it was stirred for 12 h at room temperature. The reaction mixture was concentrated under reduced pressure, and added water (10 mL), then extracted in to EtOAc, Organic layer was washed with saturated NaHCO_3_ solution and brine, dried over sodium sulphate, filtered and concentrated under reduced pressure to afford the desired product **(5)** as a brown solid (0.18 g, 45 % yield). MS (ESI) *m/z* 186.2 (M-H)^+^.

**Synthesis of 8-methyl-1-naphthonitrile (6)-** To a solution of 8-methyl-1-naphthamide (**5**, 0.175 g, 0.945 mmol) in dichloromethane (10 mL) was added triethylamine (0.429 g, 4.25 mmol) and followed by [phosphoryl chloride](https://www.google.co.in/url?url=https://en.wikipedia.org/wiki/N,N-Diisopropylethylamine&rct=j&frm=1&q=&esrc=s&sa=U&ved=0ahUKEwiG74mpkuHQAhXKyrwKHZwaDGkQFggTMAA&usg=AFQjCNEwgb90yLu_jBQYhYaZRDF_Xh9fBw) (0.57 mL, 3.78 mmol) at 0 °C, and stirred for 3 h at room temperature. The reaction mixture was diluted with dichloromethane and quenched with solid NaHCO_3_, and then extracted in to dichloromethane; Organic layer was washed with water, saturated NaHCO_3_ solution and brine, dried over sodium sulphate, filtered and concentrated under reduced pressure to get crude. The obtained crude was purified on silica column with 2% ethyl acetate in hexane as eluent to afford the desired product **(6)** as an off white solid (0.12 g, 76 % yield). ^1^H NMR (DMSO-*d*_6,_ 400 MHz) δ 2.98 (s,3H), 7.52-7.53 (m, 2H), 7.60-7.64 (m, 1H), 7.92-7.93 (m, 1H), 8.09-8.16(m, 1H), 8.26-8.32 (m, 1H).

**Synthesis of 8-(bromomethyl)-1-naphthonitrile (7)-** To a solution of 8-methyl-1-naphthonitrile (**6**, 0.12 g, 0.718 mmol) in carbon tetrachloride (10.0 mL) was added N-Bromosuccinimide (0.13 g, 0.754 mmol) at 0 °C followed by AIBN (0.01g, 0.071 mmol) and stirred for 3 h at 70 °C. After completion of the reaction, the reaction mixture was filtered through diatomaceous earth; Organic layer concentrated under reduced pressure. The obtained crude product was purified by combi-flash purifier with 3% ethyl acetate in hexane as eluent to afford the desired product **(7)** as an off-white solid (0.11 g, 45 % yield). ^1^H NMR (DMSO-*d*_6,_ 400 MHz) δ 5.48 (s,2H), 7.64 (t, *J* = 8.0 Hz, 1H), 7.70 (t, *J* = 8.0 Hz, 1H), 7.88 (d, *J* = 8.0 Hz, 1H), 8.12 (d, *J* = 8.0 Hz, 1H), 8.23 (d, *J* = 8.0 Hz, 1H), 8.37 (d, *J* = 8.0 Hz, 1H).

**Synthesis of 2-methyl-2, 3-dihydro-1H-benzo [de]isoquinolin-1-imine Hydrobromide (Compound 24)-** To a solution of 8-(bromomethyl)-1-naphthonitrile (0.05 g, 0.203 mmol) in toluene (3.0 mL), then 2M methyl amine solution in tetrahydrofuran (0.1 mL, 0.203 mmol) was added drop wise, and the reaction mixture was stirred at 50 °C for 4 h, after cooled to room temperature solid was formed which was filtered through celite pad and solid was washed with acetonitrile, pentane and diethyl ether dried under vacuum to afford the title **(Compound 24)** (0.044 g, 78% yield) as an off-white solid. ^1^H NMR (DMSO-*d*_6,_ 400 MHz) δ 3.35 (s, 3H),5.21 (s, 2H), 7.56 (d, *J* = 6.4 Hz, 1H), 7.67 (t, *J* = 8.0 Hz, 1H), 7.74 (t, *J* = 8.0 Hz, 1H), 7.98 (d, *J* = 8.0 Hz, 1H), 8.28 (d, *J* = 8.0 Hz, 1H), 8.44 (d, *J* = 8.0 Hz, 1H), 8.80 (bs, 1H), 9.35 (bs, 1H); MS (ESI) *m/z* 197.1 (M+H)^+^; HPLC Purity @ 254m, 99.71%.

**Compound (25)**

**Synthesis of ethyl 1-methyl-1H-pyrazolo [4, 3-b] pyridine-3-carboxylate (2)-** To a solution of NaH (0.35 g, 8.79 mmol) in N, N-Dimethyl formamide (10.0 mL) was added ethyl 1H-pyrazolo [4, 3-b] pyridine-3-carboxylate (**1**, 1.4 g, 7.32 mmol) at 0 °C, then stirred for half an hour at room temperature followed by adding methyl iodide (0.37 mL, 7.32 mmol) at 0 °C and the reaction mixture was stirred at room temperature for 4 h. The reaction mixture was quenched with ice, and then extracted in to EtOAc; Organic layer was washed with water, saturated NaHCO_3_ solution and brine, dried over sodium sulphate, filtered and concentrated under reduced pressure to get desired crude compound to afford the desired product **(2)** as a viscous oil (1.0 g, 66 % yield); MS (ESI) *m/z* 206.1 (M+H)^+^.

**Synthesis of 1-methyl-1H-pyrazolo [4, 3-b] pyridine-3-carboxylic acid (3)-** To a stirred solution of ethyl 1-methyl-1H-pyrazolo [4, 3-b] pyridine-3-carboxylate (**2**, 1.0 g, 4.87 mmol) in MeOH/THF/H_2_O (5.0/5.0/2.0 mL) was added LiOH.H_2_O (0.3 g, 7.31 mmol) then the reaction mixture was stirred at room temperature for 12 h. The reaction mixture was concentrated under reduced pressure to remove all the methanol portions completely, then washed with diethyl ether to remove the non-polar impurities, then the viscous portion was diluted with minimum amount of water; adjust p^H^ into 4 by using aqueous HCl solution and extracted into ethyl acetate. Organic layer was washed with water and brine, dried over sodium sulphate, filtered and concentrated under reduced pressure affords the desired product **(3)** as a pale yellow solid (0.8 g, 86 %); MS (ESI) *m/z* 178.0 (M+H)^+^.

**Synthesis of N, 1-dimethyl-1H-pyrazolo [4, 3-b] pyridine-3-carboxamide (4)-** To a solution of 1-methyl-1H-pyrazolo [4, 3-b] pyridine-3-carboxylic acid (**3**, 0.8 g, 4.51 mmol) in dichloromethane (10.0 mL) was added triethylamine (2.27 mL, 22.55 mmol) at 0 °C followed by 2M methylamine solution in THF (3.3 mL, 6.77 mmol) and 1-propane phosphoric acid cyclic anhydride (4.3 mL, 13.53 mmol) and stirred overnight at room temperature, reaction mixture was concentrated under reduced pressure, and added water (10 mL), then extracted in to EtOAc, Organic layer was washed with saturated NaHCO_3_ solution and brine, dried over sodium sulphate, filtered and concentrated under reduced pressure. The obtained crude was purified by combi-flash purifier with 3% methanol in dichloromethane as eluent to afford the desired product **(4)** as a yellow solid (0.37 g, 43 % yield). ^1^H NMR (DMSO-*d*_6,_ 400 MHz) δ 2.92 (d, *J* = 4.4 Hz, 3H), 4.14 (s, 3H), 7.50-7.53 (m, 1H), 8.28 (dd, *J* = 1.2 Hz, *J* = 8.0 Hz, 1H), 8.52 (bs, 1H), 8.66 (dd, *J* = 1.6 Hz, *J* = 6.4 Hz, 1H); MS (ESI) *m/z* 191.3 (M+H)^+^.

**Synthesis of N, 1-dimethyl-4, 5, 6, 7-tetrahydro-1H-pyrazolo [4, 3-b] pyridine-3-carboxamide (5)-** To a solution of N, 1-dimethyl-1H-pyrazolo [4, 3-b] pyridine-3-carboxamide (**4**, 0.15 g, 0.789 mmol) in ethanol (10.0 mL) was added PtO_2_ (0.1 g) then the mixture was stirred 12 h under H_2_ atmosphere at room temperature. The reaction mixture was mixture was filtered through diatomaceous earth, Organic layer concentrated under reduced pressure to afford the desired product **(5)** as a brown liquid (0.14 g, 93 %); MS (ESI) *m/z* 195.1 (M+H)^+^.

**Synthesis of N-methyl-1-(1-methyl-4, 5, 6, 7-tetrahydro-1H-pyrazolo [4, 3-b] pyridin-3-yl) methanamine (6)-** To a solution of N, 1-dimethyl-4, 5, 6, 7-tetrahydro-1H-pyrazolo [4, 3-b] pyridine-3-carboxamide (**5**, 0.13 g, 0.670 mmol) in tetrahydrofuran (10.0 mL) was added 1M LiAlH_4_ solution in tetrahydrofuran (3.35 mL, 3.35 mmol) at 0 °C and the reaction mixture was stirred at 65 °C for 12 h. After refluxing overnight the reaction mixture was quenched with brine solution (1.0 mL) at 0 °C and diluted ethyl acetate (50.0 mL), then it was filtered through diatomaceous earth and concentrated under reduced pressure to get desired crude compound **(6)** as a yellow oil (0.1 g, crude), MS (ESI) *m/z* 181.1 (M+H)^+^.

**Synthesis of 1, 4-dimethyl-1, 3, 4, 6, 7, 8-hexahydro-5H-1, 2, 4, 5a-tetraazaacenaphthylen-5-imine Hydrobromide (Compound 25)-** To a solution of N-methyl-1-(1-methyl-4, 5, 6, 7-tetrahydro-1H-pyrazolo [4, 3-b] pyridin-3-yl) methanamine (**6**, 0.1 g, 0.55 mmol) in ethanol (5.0 mL), then cyanogen bromide (0.07 g, 0.66 mmol) was added, and the reaction mixture was stirred at 85 °C for 3 h, after cooled to room temperature reaction mixture was concentrated under reduced pressure. The obtained crude was purified by combi-flash purifier with 7% methanol in dichloromethane as eluent to afford the desired product **(Compound 25)** as a yellow solid (0.03 g, 18 % yield). ^1^H NMR (DMSO-*d*_6,_ 400 MHz) δ 2.00-2.04 (m, 2H), 2.66-2.69 (m, 2H), 3.05 (s, 3H), 3.65-3.68 (m, 4H), 4.64 (s, 2H), 7.81 (bs, 2H); MS (ESI) *m/z* 206.3 (M+H)^+^;HPLC Purity @ 254nm, 99.99%.

**Compound (26)**

**Synthesis of ethyl 2-methyl-2H-pyrazolo [4, 3-b] pyridine-3-carboxylate (2)-** To a solution of NaH (0.35 g, 8.79 mmol) in N, N-Dimethyl formamide (10.0 mL) was added ethyl 1H-pyrazolo [4, 3-b] pyridine-3-carboxylate (**1**, 1.4 g, 7.32 mmol) at 0 °C, then stirred for half an hour at room temperature followed by adding methyl iodide (0.37 mL, 7.32 mmol) at 0 °C and the reaction mixture was stirred at room temperature for 4 h. The reaction mixture was quenched with ice, and then extracted in to EtOAc; Organic layer was washed with water, saturated NaHCO_3_ solution and brine, dried over sodium sulphate, filtered and concentrated under reduced pressure to get desired crude compound to afford the desired product **(2)** as a viscous oil (1.0 g, 66 % yield); MS (ESI) *m/z* 206.1 (M+H)^+^.

**Synthesis of 2-methyl-2H-pyrazolo[4, 3-b] pyridine-3-carboxylic acid (3)-** To a solution of ethyl 2-methyl-2H-pyrazolo[4,3-b]pyridine-3-carboxylate(**2**, 1.0 g, 4.87 mmol) in MeOH: THF:H_2_O (5.0:5.0:2.0 mL) was added lithium hydroxide (0.3 g, 7.31 mmol) then the reaction mixture was stirred at room temperature for 12 h. The reaction mixture was concentrated under reduced pressure then washed with diethyl ether to remove the non-polar impurities, then the viscous portion was diluted with minimum amount of water, adjusted P^H^ ~ 4 by using aqueous HCl solution and extracted into ethyl acetate. Organic layer was washed with water and brine, dried over sodium sulphate, filtered and concentrated under reduced pressure affords the desired product **(3)** as a pale yellow solid (0.8 g, 86 % yield); MS (ESI) *m/z* 178.0 (M+H)^+^.

**Synthesis of N, 2-dimethyl-2H-pyrazolo [4, 3-b] pyridine-3-carboxamide (4)-** To a solution of 2-methyl-2H-pyrazolo [4, 3-b] pyridine-3-carboxylic acid (**3**, 0.8 g, 4.51 mmol) in dichloromethane (10.0 mL) was added triethylamine (2.27 mL, 22.55 mmol) at 0 °C followed by 2M methylamine solution in tetrahydrofuran (3.3 mL, 6.77 mmol) and 1-propane phosphoric acid cyclic anhydride (4.3 mL, 13.53 mmol), and stirred for 12 h at room temperature, reaction mixture was concentrated under reduced pressure, and added water (10 mL), then extracted in to EtOAc, Organic layer was washed with saturated NaHCO_3_ solution and brine, dried over sodium sulphate, filtered and concentrated under reduced pressure. The obtained crude was purified by combi-flash purifier with 3% methanol in dichloromethane as eluent to afford the desired product **(4)** as a yellow solid (0.37 g, 43 % yield). ^1^H NMR (DMSO-*d*_6,_ 400 MHz) δ 2.96 (d, *J* = 4.8 Hz, 3H), 4.49 (s, 3H), 7.41-7.44 (m, 1H), 8.25(d, *J* = 8.8 Hz, 1H), 8.69 (d, *J* = 4.0 Hz, 1H),8.78 (bs, 1H); MS (ESI) *m/z* 191.3 (M+H)^+^.

**Synthesis of N, 2-dimethyl-4, 5, 6, 7-tetrahydro-2H-pyrazolo [4, 3-b] pyridine-3-carboxamide (5)-** To a solution of N, 2-dimethyl-2H-pyrazolo [4, 3-b] pyridine-3-carboxamide (**4**, 0.24 g, 1.26 mmol) in ethanol (10.0 mL) was added PtO_2_ (0.1 g), then the mixture was stirred 12 h under H_2_ atmosphere at room temperature. The reaction mixture was mixture was filtered through diatomaceous earth; Organic layer concentrated under reduced pressure to afford the desired product **(5)** as a brown liquid (0.22 g, 89% yield). ^1^H NMR (DMSO-*d*_6,_ 400 MHz) δ 1.71-1.74 (m, 2H), 2.53-2.56 (m, 2H), 2.72 (d, *J* = 4.4 Hz, 3H), 3.00-3.02 (m,2H), 3.83 (s, 3H), 4.62 (s, 1H), 7.28 (s, 1H); MS (ESI) *m/z* 195.3 (M+H)^+^.

**Synthesis of N-methyl-1-(2-methyl-4, 5, 6, 7-tetrahydro-2H-pyrazolo [4, 3-b] pyridin-3-yl) methanamine (6)-** To a solution of N, 2-dimethyl-4, 5, 6, 7-tetrahydro-2H-pyrazolo [4, 3-b] pyridine-3-carboxamide (**5**, 0.21g, 1.08 mmol) in anhydrous tetrahydrofuran (10.0 mL) was added 1M LiAlH_4_ solution in tetrahydrofuran (5.41 mL, 5.41 mmol) at 0 °C and the reaction mixture was stirred at 65 °C for 48 h. After refluxing 48 h the reaction mixture was quenched with brine solution (1.0 mL) at 0 °C and diluted ethyl acetate, then it was filtered through diatomaceous earth and concentrated under reduced pressure to get desired crude compound **(6)** as a yellow oil (0.17 g, crude), MS (ESI) *m/z* 181.1 (M+H)^+^.

**Synthesis of 1, 7-dimethyl-1, 3, 4, 5, 7, 8-hexahydro-6H-1, 2, 5a, 7-tetraazaacenaphthylen-6-imine hydrobromide (Compound 26)-** To a solution of N-methyl-1-(2-methyl-4, 5, 6, 7-tetrahydro-2H-pyrazolo [4, 3-b] pyridin-3-yl) methanamine (**6**, 0.15 g, 0.83 mmol) in ethanol (10.0 mL), then cyanogen bromide (0.07 g, 0.66 mmol) was added, and the reaction mixture was stirred at 85 °C for 3 h, after cooled to room temperature reaction mixture was concentrated under reduced pressure. The obtained crude was purified by combi-flash purifier with 7% methanol in dichloromethane as eluent to afford the desired product **(Compound 26)** as a yellow solid (0.02 g, 10 % yield). ^1^H NMR (DMSO-*d*_6,_ 400 MHz) δ 1.98-2.00 (m, 2H), 2.46-2.50 (m, 2H), 2.552.58 (m, 3H), 3.04 (s,3H), 3.63-3.65 (m, 4H), 7.84 (bs, 2H); MS (ESI) *m/z* 206.3 (M+H)^+^;HPLC Purity @ 220nm, 99.63%.

**Compound (27)**

**Synthesis of tert-butyl 7-formyl-1H-indole-1-carboxylate (2)-** To a solution of 1H-indole-7-carbaldehyde (**1**, 3 g, 20.66 mmol) in tetrahydrofuran (40 mL) were added di-tert-butyl dicarbonate (7.12 mL, 31.0 mmol) and 4-dimethylaminopyridine (0.025 g, 0.2 mmol). The reaction mixture was stirred at room temperature for 16 h. The reaction mixture was concentrated completely under reduced pressure to give the crude residue which was purified by combi flash purifier using 3% ethyl acetate/hexane as eluent to give the title compound **(2)** as a white solid (4.95 g, 97.8 % yield). ^1^H NMR (CDCl_3,_ 400 MHz) δ 1.63 (s, 9H), 6.65 (d, *J* = 9 Hz, 1H), 7.34 (t, *J* = 7.6 Hz, 1H), 7.61 (d, *J* = 4.0 Hz, 1H), 7.74 (d, *J* = 7.6 Hz, 1H), 10.57 (s, 1H); MS (ESI) *m/z* 146.1 (M+H) ^+^-BOC.

**Synthesis of tert-butyl 7-(cyano((ethoxycarbonyl)oxy)methyl)-1H-indole-1-carboxylate (3)-** To a solution of tert-butyl 7-formyl-1H-indole-1-carboxylate (**2**, 3.3 g, 13.46 mmol) in 70 mL of dichloromethane were added ethyl carbonochloridate (3.2 g, 29.61 mmol), tetrabutylammonium chloride (0.037 g, 0.134 mmol), sodium cyanide (3.3 g, 67.3 mmol) and water (15 mL). The reaction mixture was stirred at room temperature for 16 h. The reaction mixture was diluted with water and was extracted with dichloromethane. The combined organic layer washed with sodium bicarbonate solution, brine solution and dried over anhydrous sodium sulphate, concentrated solvent under reduced pressure to give crude residue which was purified by combiflash purifier using 8 % ethyl acetate/hexane as eluent to give the title compound **(3)** as an off-white solid (4.5 g, 97.2 % yield). ^1^H NMR (CDCl_3,_ 400 MHz) δ 1.33 (t, *J* = 7.2 Hz, 3H), 1.63 (s, 9H), 4.25-4.27 (m, 2H), 6.59 (d, *J* = 3.6 Hz, 1H) 7.34 (d, *J* = 8.0 Hz, 1H), 7.58 (d, *J* = 4.0 Hz, 1H), 7.61 (s, 1H), 7.63 (d, *J* = 7.6 Hz, 1H), 7.72 (d, *J* = 7.6 Hz, 1H);

**Synthesis of tert-butyl 7-(cyanomethyl)-1H-indole-1-carboxylate (4)-** To a solution of tert-butyl 7-(cyano((ethoxycarbonyl)oxy)methyl)-1H-indole-1-carboxylate (**3**, 4 g, 11.6 mmol) in ethyl acetat (40 mL) was added Pd/C (10%) under nitrogen atmosphere, then the Parr shaker vessel was stirred at 50 PSI at room temperature. The reaction mixture was filtered through celite washed with ethyl acetate, the filtrate was concentrated under reduced pressure to give crude residue which was purified by column chromatography over silica gel using 3% ethyl acetate/hexane as eluent to give the title compound **(4)** as an off-white solid (0.6 g, 20.2 % yield). ^1^H NMR (CDCl_3,_ 400 MHz) δ 1.65 (s, 9H), 4.32 (s, 2H), 6.57 (d, *J* = 4.0 Hz, 1H), 7.21-7.29 (m, 2H) 7.56 (d, *J* = 3.6 Hz, 1H); MS (ESI) *m/z* 257.3 (M+H) ^+^.

**Synthesis of 2-(1H-indol-7-yl) acetic acid (5)-**To a solution of tert-butyl 7-(cyanomethyl)-1H-indole-1-carboxylate (**4**, 0.4 g, 1.56 mmol) in methanol (8 mL) was added sodium hydroxide (0.187 g, 4.68 mmol) and water (2 mL). The reaction mixture was stirred at 80 °C for 16 h. After completion of the reaction, cooled to room temperature, concentrated the solvent and which was acidified using 1N HCl solution (P^H^-3 to 4), and it was extracted with ethyl acetate. The combined organic layer washed with sodium bicarbonate solution, brine solution and dried over anhydrous sodium sulphate, concentrated solvent under reduced pressure to give crude residue **(5)** as an off-white solid which was used to next step without any purification (0.35 g, 81.5 % yield). MS (ESI) *m/z* 176.1 (M+H) ^+^.

**Synthesis of 2-(1H-indol-7-yl)-N-methylacetamide (6)-** To a solution of 2-(1H-indol-7-yl)acetic acid (**5**, 0.3 g, 1.713 mmol) in dimethylformamide (10 mL) were added 2M methyl amine (1.28 mL, 2.57 mmol), HATU (1.6 g, 4.28 mmol) and *N,N-d*iisopropylethylamine (0.897 mL, 5.14 mmol). The reaction mixture was stirred at room temperature for 16 h. The reaction mixture was diluted with ice water and extracted with ethyl acetate. The combined organic layer washed with brine solution and dried over anhydrous sodium sulphate, concentrated solvent under reduced pressure to give crude residue which was purified by combiflash purifier using 50 % ethyl acetate /hexane as eluent to give the title compound **(6)** as an off white solid (0.19 g, 38.5 % yield). ^1^H NMR (CDCl_3,_ 400 MHz) δ 2.56 (d, *J* = 4.4 Hz, 3H), 3.64 (s, 2H), 6.40 (s, 2H), 6.89-6.92 (m, 2H), 7.31 (s, 1H), 7.38-7.40 (m, 1H), 6.85 (bs, 1H), 10.86 (s, 1H); MS (ESI) *m/z* 189.1 (M+H) ^+^.

**Synthesis of 5,6-difluoroindoline (7)-** To a suspension of 2-(1H-indol-7-yl)-N-methylacetamide (**6**, 0.17 g, 0.90 mmol) in acetic acid (5 mL) was cooled to 10 °C, sodium cyanoborohydride (0.17 g, 2.7 mmol) was added in portion wise. Then reaction mixture was stirred at room temperature for 1 h. Diluted with water and basified using ammonia solution, and was extracted with ethyl acetate. The combined organic layer washed with brine solution and dried over anhydrous sodium sulphate, concentrated solvent under reduced pressure to give crude residue **(7)** as a yellow oily mass which was used to next step without any purification (0.12 g, crude. MS (ESI) *m/z* 191.1 (M+H) ^+^.

# Synthesis of 2-(indolin-7-yl)-N-methylethan-1-amine (8)- To a solution of 2-(indolin-7-yl)-N-methylacetamide (7, 0.12 g, 0.63 mmol) in tetrahydrofuran (15 mL) was cooled to 0 °C, then1M LAH solution in tetrahydrofuran (3.15 mL, 3.15 mmol) was added and the reaction mixture was heated at 65 °C for 16 h. The reaction mixture was cooled to room temperature and slowly quenched with ammonium chloride solution at 0 °C, added ethyl acetate and stirred for 0.5 h. The precipitate was filtered, the filtrate was extracted. The combined organic layer washed with brine solution and dried over anhydrous sodium sulphate, concentrated solvent under reduced pressure to give crude residue (8) as a sticky mass which was used next step without any purification (0.08 g, crude). MS (ESI) *m/z* 177.1 (M+H) ^+^.

**Synthesis of 3-methyl-2,3,6,7-tetrahydro-[1,3]diazepino[6,7,1-hi]indol-4(1H)-imine hydrobromide (Compound 27)-** To a solution of 2-(indolin-7-yl)-N-methylethan-1-amine (**8**, 0.08 g, 0.453 mmol) in ethanol (4 mL) was added cyanogen bromide (0.057 g, 1.76 mmol). Then the reaction mixture was heated at 80 °C for 1 h. The reaction mixture was cooled to room temperature and concentrated solvent completely give crude residue which was purified using which was purified by combi flash purifier using 8 % methanol/dichloromethane as eluent to give the title **(Compound 27)** as a white solid (0.013 g, 10.2 % yield). ^1^H NMR (DMSO-*d_6_*_,_ 400 MHz) δ 2.97-3.00 (m, 2H), 3.13-3.16 (m, 5H), 3.52-3.54 (m, 2H), 4.12 (t, *J* = 8.8 Hz, 2H), 6.99 (t, *J* = 6.8 Hz, 1H), 7.06 (d, *J* = 7.6 Hz, 1H), 7.15 (d, *J* = 7.2 Hz, 1H), 7.86 (bs, 2H); MS (ESI) *m/z* 202.1 (M+H)^+^-HBr. HPLC purity @ 250 nm, 98.61 %.
